# The transcriptional reprograming and functional identification of WRKY family members in pepper’s response to *Phytophthora capsici* infection

**DOI:** 10.1186/s12870-020-02464-7

**Published:** 2020-06-03

**Authors:** Wei Cheng, Yan Jiang, Jiangtao Peng, Jianwen Guo, Menglan Lin, Chengting Jin, Jinfeng Huang, Weiqi Tang, Deyi Guan, Shuilin He

**Affiliations:** 1 National Education Minister Key Laboratory of Plant Genetic Improvement and Comprehensive Utilization, Fujian Agriculture and Forestry University, Fuzhou, 350002 Fujian China; 2 Key Laboratory of Applied Genetics of Universities in Fujian Province, Fujian Agriculture and Forestry University, Fuzhou, 350002 Fujian China; 3 College of Agriculture, Fujian Agriculture and Forestry University, Fuzhou, 350002 Fujian China

**Keywords:** Pepper, *Phytophthora*, WRKY, Transcription factor, Transcriptome, Transcriptional reprograming, Disease resistance

## Abstract

**Background:**

Plant transcription factors (TFs) are key transcriptional regulators to manipulate the regulatory network of host immunity. However, the globally transcriptional reprogramming of plant TF families in response to pathogens, especially between the resistant and susceptible host plants, remains largely unknown.

**Results:**

Here, we performed time-series RNA-seq from a resistant pepper line CM334 and a susceptible pepper line EC01 upon challenged with *Phytophthora capsici*, and enrichment analysis indicated that WRKY family most significantly enriched in both CM334 and EC01. Interestingly, we found that nearly half of the WRKY family members were significantly up-regulated, whereas none of them were down-regulated in the two lines. These induced WRKY genes were greatly overlapped between CM334 and EC01. More strikingly, most of these induced WRKY genes were expressed in time-order patterns, and could be mainly divided into three subgroups: early response (3 h-up), mid response (24 h-up) and mid-late response (ML-up) genes. Moreover, it was found that the responses of these ML-up genes were several hours delayed in EC01. Furthermore, a total of 19 induced WRKY genes were selected for functional identification by virus-induced gene silencing. The result revealed that silencing of *CaWRKY03–6*, *CaWRKY03–7*, *CaWRKY06–5* or *CaWRKY10–4* significantly increase the susceptibility to *P. capsici* both in CM334 and EC01, indicating that they might contribute to pepper’s basal defense against *P. capsici*; while silencing of *CaWRKY08–4* and *CaWRKY01–10* significantly impaired the disease resistance in CM334 but not in EC01, suggesting that these two WRKY genes are prominent modulators specifically in the resistant pepper plants.

**Conclusions:**

These results considerably extend our understanding of WRKY gene family in pepper’s resistance against *P. capsici* and provide potential applications for genetic improvement against phytophthora blight.

## Background

During co-evolution with diverse pathogens, plants have evolved a highly sophisticated and effective innate immune system to protect themselves against the pathogenic invaders. This system consists of two primary layers [1, 2], the first layer of plant immunity is triggered upon perception of highly conserved pathogen-associated molecular patterns (PAMPs) via plant pattern recognition receptors (PRRs) and is termed as PAMP-triggered immunity (PTI). PTI can be attenuated or blocked by effectors that are secreted into host cells by some adapted pathogens. The remaining weakened plant immunity during such compatible interactions is defined as basal defense, which is also activated in susceptible plants; however, it is not sufficient to prevent disease propagation [3]. The second layer of plant immunity is triggered by host receptors encoded by resistance (R) genes upon recognizing pathogen-delivered effectors either directly or indirectly and is termed as effector-triggered immunity (ETI), which bring out a more robust defense response and often accompanied by hypersensitive response [2]. Despite with variations in the magnitude and duration of immune responses, PTI and ETI share some common signaling components such as reactive oxygen species (ROS), MAPK cascades, phytohormones [4–7]. These defense signaling are generally integrated and relayed into appropriate immune outputs by the action of various transcription factors (TFs).

Both PTI and ETI are largely regulated at transcriptional level with the action of various plant TFs constituting transcriptional networks [8]. Over the past few decades, a large number of plant TFs, particularly in the model plants *Arabidopsis* and rice, have been functionally characterized to play important roles in modulating defense response [9, 10]. Accumulating data indicate that some plant TF families such as AP2/ERF, bHLH, bZIP, NAC and WRKY are key regulators in the defense processes [4, 9]. However, previous studies focused primarily on the functional characterization of individual TFs in host immune response, a genome-wide and systematic comparative analysis of certain plant TF families, especially between the resistant and susceptible host plants, will be valuable for elucidating their regulatory relationships during the pathogen infection.

Pepper (*Capsicum annuum*) is an economically important crop worldwide. Phytophthora blight of pepper is a devastating disease caused by the oomycete pathogen *Phytophthora capsici* [11, 12]. This pathogen can infect all parts of the pepper plant, including the roots, leaves and fruits [11, 13]. The disease frequently reaches epidemic levels and causes huge yield losses in pepper production regions. In plant-*Phytophthora* interaction system, recently several WRKY TFs have been identified to play important roles in plant defense against *Phytophthora* species. For example, WRKY TFs from *Nicotiana benthamiana* could be phosphorylated by MAPK and regulate immunity to *P. infestans* mediated by RBOHB-dependent ROS burst [14]. In *Glycine max*, GmWRKY31 and GmWRKY40 were identified in resistance to *P. sojae* [15, 16]. In *Solanum tuberosum*, StWRKY1 and StWRKY8 regulate phenylpropanoid and benzylisoquinoline alkaloid pathway conferring resistance to *P. infestans*, respectively [17, 18]. In *Solanum pimpinellifolium*, eight WRKY TFs were identified to be involved in response to *P. infestans* infection by transcriptome analysis, and SpWRKY3 was found to act a positive modulator in resistance to late blight disease [19]. Overexpression of SpWRKY1 in tobacco and tomato conferred increased resistance to *P. nicotianae* and *P. infestans*, respectively [20–22]. Loss and gain of function analysis also indicated that SpWRKY6 acts as a positive regulator in tomato resistance to *P. infestans* infection [23]. Importantly, these results also indicate that a subset of WRKY TFs might be involved in response to a single pathogen infection. In *Capsicum annuum*, although phytophthora blight caused by *P. capsici* is one of the most important diseases worldwide, the globally transcriptional reprogramming and functional identification of WRKY family members in defense against the pathogen remain largely unknown.

Herein, we performed time-series RNA-seq from a resistant pepper line CM334 and a susceptible pepper line EC01 upon challenged with *P. capsici*. The objective of this study was to identify key TF families and their family members involved in pepper defense against *P. capsici* infection and provide new insights into plant defense signaling regulation.

## Results

### High-throughput RNA sequencing and DEG analysis

**Fig. 1:**
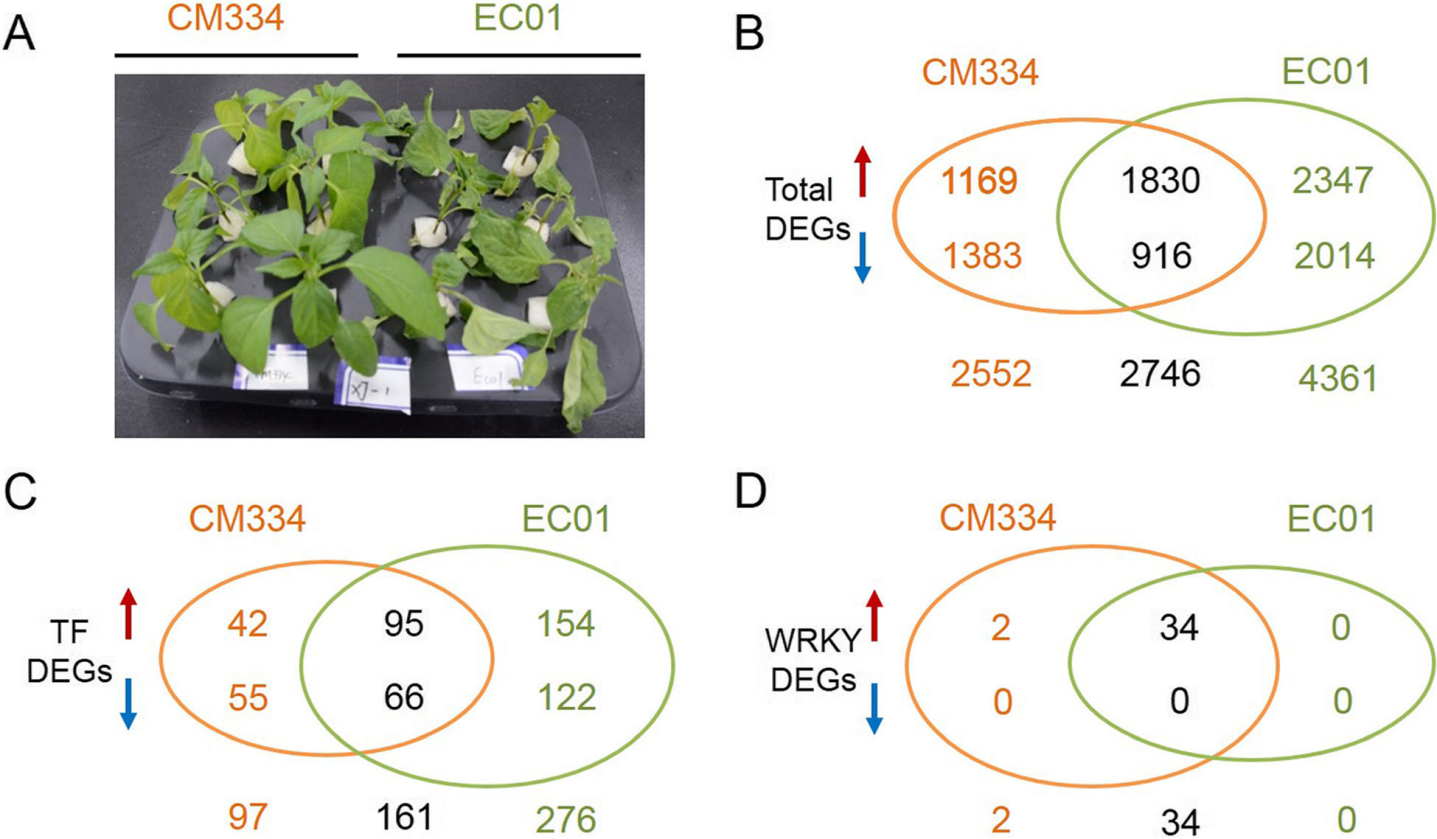
Phenotypic characterization and DEGs identification between pepper lines CM334 and EC01 after *P. capsici* infection. **a** Disease symptoms of the two pepper lines CM334 and EC01 at 3 days post-inoculation with *P. capsici*. **b** In total, 5298 and 7107 differentially expressed genes (DEGs) were identified from CM334 and EC01 respectively and 2746 DEGs were commonly identified in the both lines. **c** Among all the transcription factors (TFs), 258 and 437 TF DEGs were identified from CM334 and EC01 respectively, and 161 TF DEGs were commonly identified in the both lines. **d** Among all WRKY TFs, 36 and 34 WRKY DEGs were identified from CM334 and EC01 respectively, and two CaWRKY genes were up-regulated specifically in CM334. The upward arrow represents the up-regulated expression. The downward arrow represents the down-regulated expression

To genome-wide investigate transcriptional regulation mechanism of pepper in response to *P. capsici* infection, time-series RNA-seq data from the resistant line CM334 and the susceptible line EC01 at 0, 3, 6, 12, 24, 48, and 72 h post inoculation (hpi) were analyzed. As shown in Fig. 1a, serious disease symptoms (wilt phenotype) were observed in the susceptible pepper line EC01 when inoculated with the virulent *P. capsici* stain JX1 but not in the resistant line CM334, which was in agreement with the result of the previous report [24]. Illumina-based next-generation sequencing was performed from pepper roots infected with *P. capsici*. In total, 42 samples (2 genotypes × 7 time points × 3 biological replicates) were collected for library construction. Approximately 80.2 million and 84.6 million raw reads were generated respectively from each sample of CM334 and EC01 (Additional file 1: Dataset S1). After filtered with low-quality reads, approximately 79.6 million and 84.0 million clean reads were obtained from each sample of CM334 and EC01, respectively. On the average, more than 69.9 million (87.8%) and 68.0 million (80.1%) unique mapped reads from each sample of CM334 and EC01 were respectively aligned to the pepper CM334 genome version PEP (v1.6). Following alignment to each gene model, we normalized the number of mapped reads to fragments per kilobase million (FPKM). We identified differentially expressed genes (DEGs, with fold change > 2 and FDR ≤ 0.01) between the inoculated and mock-inoculated samples. Approximately 4075 DEGs with 2530 up-regulated and 1545 down-regulated genes were identified in CM334. In comparison, 5962 DEGs with 3732 up-regulated and 2230 down-regulated genes were identified in EC01 (Fig. 1b).

### Global analysis of TF families in pepper’s response to *P. capsici* infection

As TFs play important roles in plant defense signaling, we investigated the transcriptional changes of all pepper TF families in response to *P. capsici* infection. In *Capsicum annuum* genome, ~ 1665 TFs were identified and classified into 58 families according to PlantTFDB V5.0 [25]. Upon *P. capsici* inoculation, 258 TF genes (with 137 up-regulated and 121 down-regulated TF genes), which account for ~ 15.5% of all identified TF genes, were differentially expressed in the resistant pepper line CM334; while in the susceptible pepper line EC01, 437 TF genes (with 249 up-regulated and 188 down-regulated TF genes), which account for ~ 26.2% of all identified TF genes, were significantly altered during the infection (Fig. 1c). These results indicated that there are dramatic transcriptional changes both in CM334 and EC01 during *P. capsici* infection, while with more DEGs and differential expressed TFs in EC01.

**Fig. 2:**
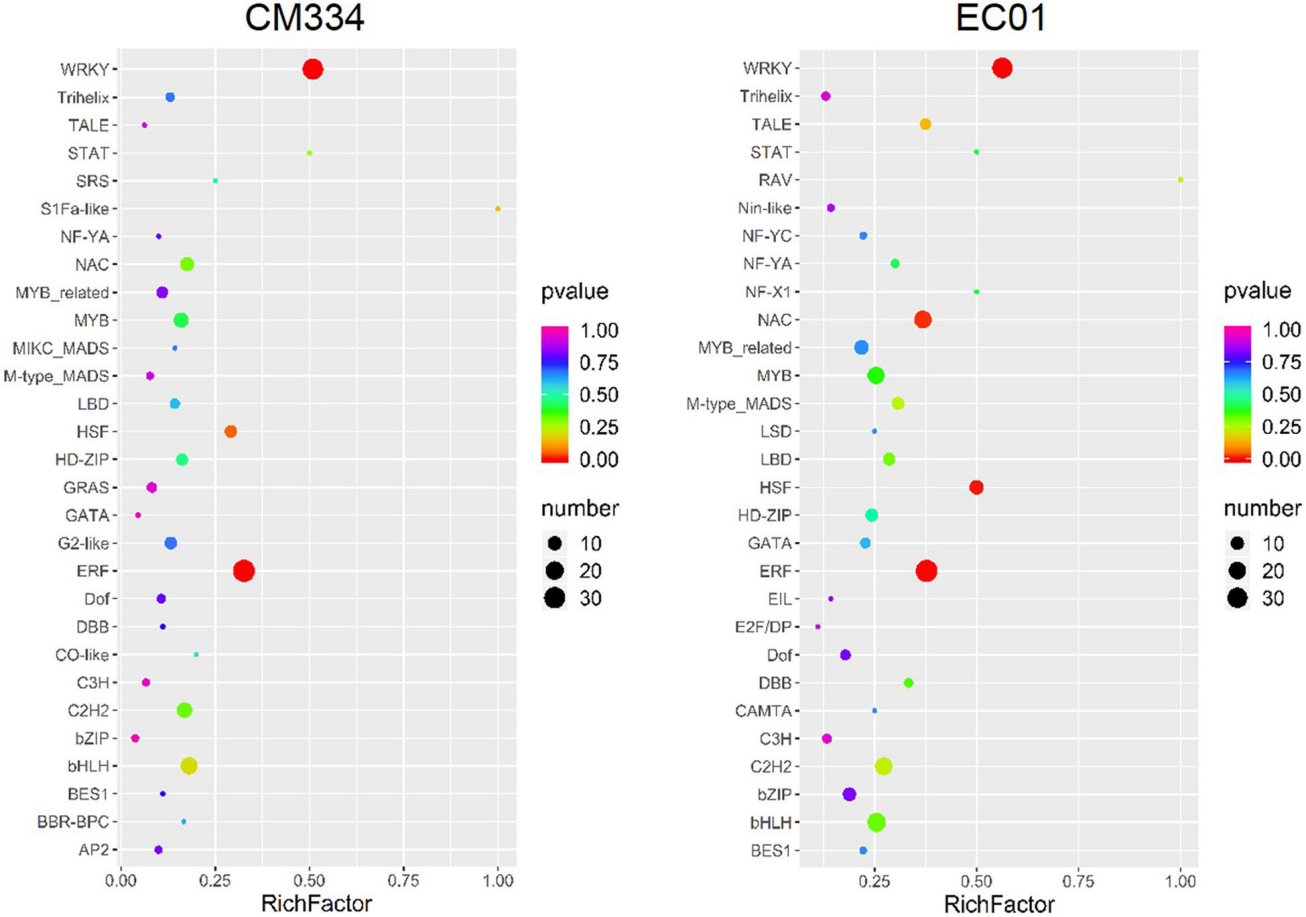
Enrichment analysis of up-regulated TF DEGs between pepper lines CM334 and EC01 after *P. capsici* infection. To gain insights into the 58 TF families in response to *P. capsici* infection, enrichment analysis was performed using all up-regulated TF DEGs in the two lines. Among all these TF families, WRKY family was most significantly enriched both in CM334 and EC01. The enrichment analysis was performed by ggplot2 package (http://had.co.nz/ggplot2/)

Previous reports have suggested that some plant TF families such as AP2/ERF, bHLH, bZIP, NAC and WRKY are key regulators in defense response [4, 9]. To gain insights into the 58 TF families in response to *P. capsici* infection, we performed enrichment analysis using the identified TF DEGs in the two lines. Among all these TF families, it was found that WRKY family was the most significantly enriched both in CM334 and EC01 (Fig. 2 and Additional file 2: Figure S1). In-depth analysis showed that nearly half of the WRKY genes were significantly induced in the resistant line CM334 (36, ~ 50.0%) and the susceptible line EC01 (34, ~ 47.2%). More strikingly, none of the WRKY genes were significantly down-regulated in the both lines during the infection (Table 1 and Fig. 1d). Our comparative transcriptomic analysis suggested that WRKY may play a more critical role in modulating the host transcriptional immune response to *P. capsici*, and thus we then focused primarily on the WRKY family in the subsequent study.

**Table 1:** Global analysis of TF families in pepper’s response to *P. capsici* infection

	WRKY (72)	bZIP (53)	bHLH (129)	NAC (96)	MYB (107)	AP2/ERF (143)	All TFs (1665)
CM334-up	36 (50.0%)	1 (1.9%)	10 (7.8%)	8 (8.3%)	12 (11.2%)	22 (15.4%)	137 (8.2%)
CM334-down	0 (0.0%)	5 (9.4%)	9 (7.0%)	1 (1.0%)	3 (2.8%)	6 (4.2%)	121 (7.3%)
EC01-up	34 (47.2%)	8 (15.1%)	20 (15.5%)	20 (20.8%)	17 (15.9%)	36 (25.2%)	249 (15.0%)
EC01-down	0 (0.0%)	11 (20.8%)	18 (14.0%)	1 (1.0%)	8 (7.5%)	12 (8.4%)	188 (11.3%)

### Transcriptome analysis of WRKY family members in pepper during *P. capsici* infection

**Fig. 3:**
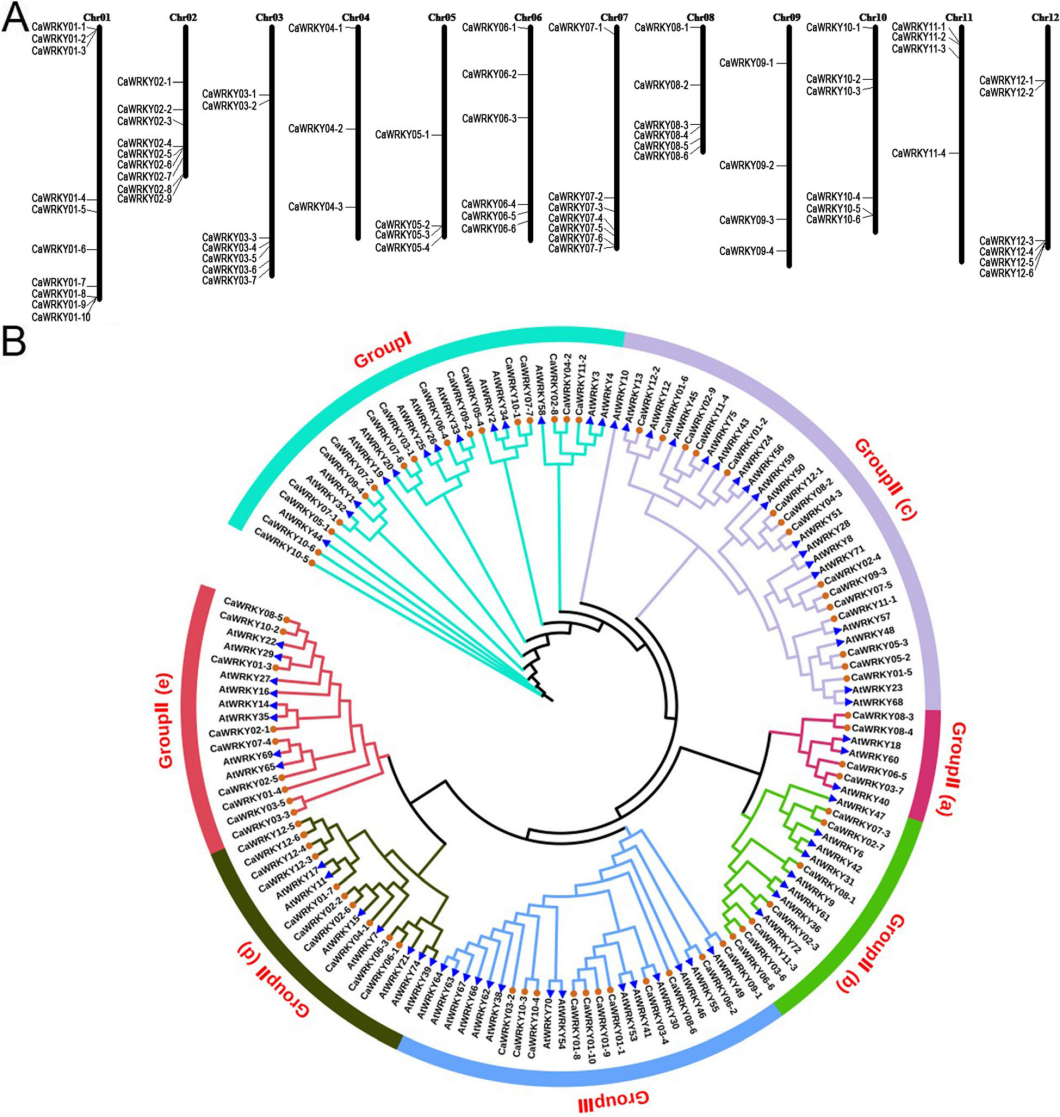
Chromosomal location and phylogenetic tree analysis of CaWRKY family members. **a** All identified 72 WRKYs from *Capsicum annuum* (CaWRKYs) were mapped to the ‘CM334’ chromosomes in the pepper genome database using BLASTn. The MapInspect software (http://mapinspect.software.informer.com/) was used to map the gene locus on chromosomes. **b** A multiple alignment of the 72 CaWRKYs and 71 AtWRKYs from *Arabidopsis thaliana* was performed using ClustalX2 (http://www.clustal.org/clustal2/). The alignment result was used to construct a phylogenetic tree using the neighbor-joining method of PhyML software (http://www.atgc-montpellier.fr/phyml/)

A total of 72 putative CaWRKY genes that contained the conserved WRKY domain were identified according to the pepper CM334 genome version PEP (v1.6). Due to their low homology with AtWRKYs from *Arabidopsis* and also avoid naming confusion [26, 27], we designated all these CaWRKY genes from CaWRKY01–1 to CaWRKY12–6 according to their location of chromosomes. The chromosomal distribution of these CaWRKY genes was shown in Fig. 3. The detailed information about these CaWRKY genes, including gene loci accession number in PEP (v1.6), WRKYGOK heptapeptide stretch, zinc-finger motif type and gene classification, was listed in Additional file 3: Dataset S2. The nucleotide and protein sequences of CaWRKY members were listed in Additional file 4: Dataset S3. The phylogenetic relationship between these 72 CaWRKY and 71 AtWRKYs was analyzed by multiple sequence alignment (Fig. 3). The result indicated that only 15 CaWRKYs exhibit a high similarity with their *Arabidopsis* WRKY orthologs.

**Fig. 4:**
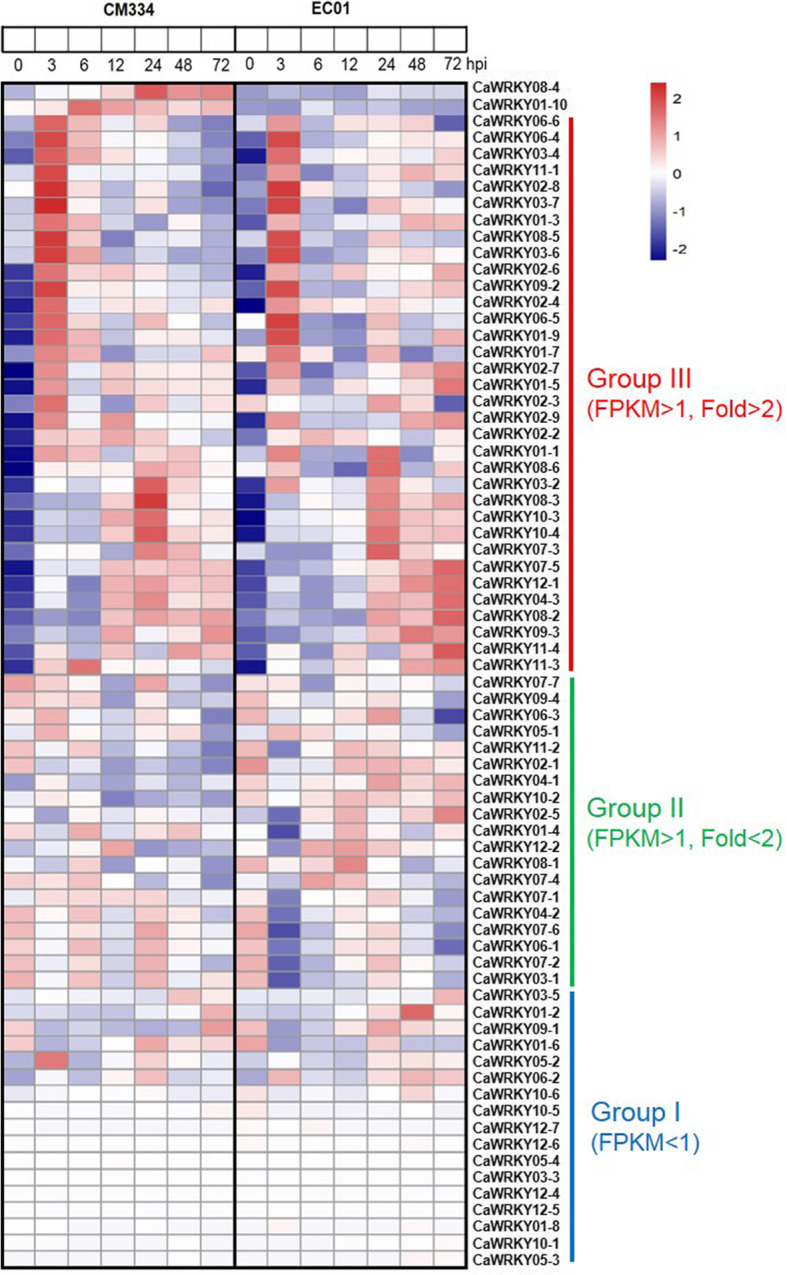
The heat map of WRKY family members in pepper’s response to *P. capsici* infection. Base on the transcript abundance and dynamic changes of gene expression, the 72 CaWRKY family members could be mainly classified into three groups: group I, were not or very low expressed (FPKM < 1); group II, were more-or-less constitutively expressed (FPKM > 1, fold < 2); group III, were significantly up-regulated during the infection (FPKM > 1, fold > 2). The Z-scores of RNA-seq data sets were used for analysis of gene expression patterns using the ggplot2 package (https://cran.r-project.org/web/packages/ggplot2/) and construction of heat maps using the pheatmap package (https://cran.r-project.org/web/packages/pheatmap/)

**Fig. 5:**
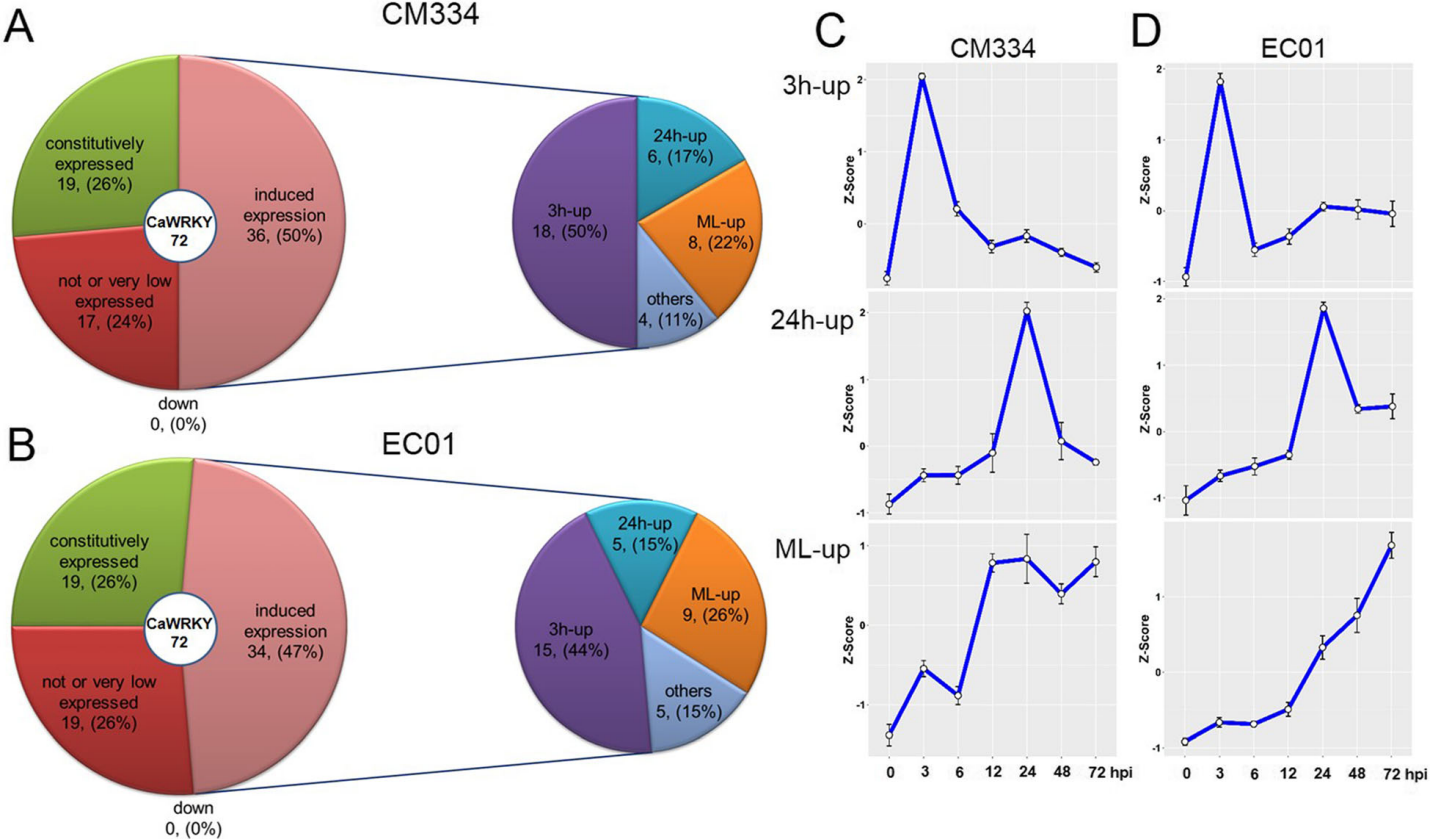
Transcriptional patterns of the induced WRKY family members in pepper during *P. capsici* infection. These up-regulated CaWRKY genes can be mainly separated into three subgroups: early response (3 h-up), mid response (24 h-up), and mid-late response (ML-up) genes in (**a**) CM334 and (**b**) EC01. The transcriptional patterns of the three subgroup WRKY members in CM334 and EC01 are showed in (**c**) and (**d**), respectively. The Z-scores of RNA-seq data sets were used for analysis of gene expression patterns using the ggplot2 package (https://cran.r-project.org/web/packages/ggplot2/)

Base on the transcript abundance and dynamic changes of gene expression, these CaWRKY family members could be classified into three groups (Figs. 4 and 5a, b); group I, which included 17 and 19 CaWRKY genes in CM334 and EC01 respectively, were not or very low expressed (FPKM < 1.0); group II, which included 19 CaWRKY genes in both lines, were more-or-less constitutively expressed with similar transcript levels between inoculated and mock-inoculated plants; group III, which included 36 and 34 CaWRKY genes in CM334 and EC01 respectively, were significantly up-regulated during the infection. It was worth noting that the repertoires of these induced CaWRKY genes were greatly overlapped in CM334 and EC01, excepting that two CaWRKYs (CaWRKY08–4 and CaWRKY01–10) were resistant line specific up-regulated.

More interestingly, most of these induced CaWRKY genes could be mainly separated into three subgroups: early response (3 h-up), mid response (24 h-up), and mid-late response (ML-up) genes (Fig. 5c, d and Additional file 5: Dataset S4). Among the 36 up-regulated CaWRKY genes in CM334, it was found that 18 of them had peaks of transcriptional induction at 3 hpi, 6 of them showed the highest transcripts level at 24 hpi, and 8 of them were mid-late response genes which sustain transcriptional induction at 12 ~ 72 hpi. Among the 34 up-regulated CaWRKY genes in EC01, 15 of them exhibited the highest transcripts level at 3 hpi, 5 of them showed the highest transcripts level at 24 hpi, and 9 of them were ML-up genes but with several hours delay. Our time-resolved transcriptome analysis suggested that 3 and 24 hpi are two of critical time points in the transcriptional reprograming of WRKY family members in pepper’s response to *P. capsici* infection.

### Functional identification of WRKY family members in pepper defense against *P. capsici*

**Fig. 6:**
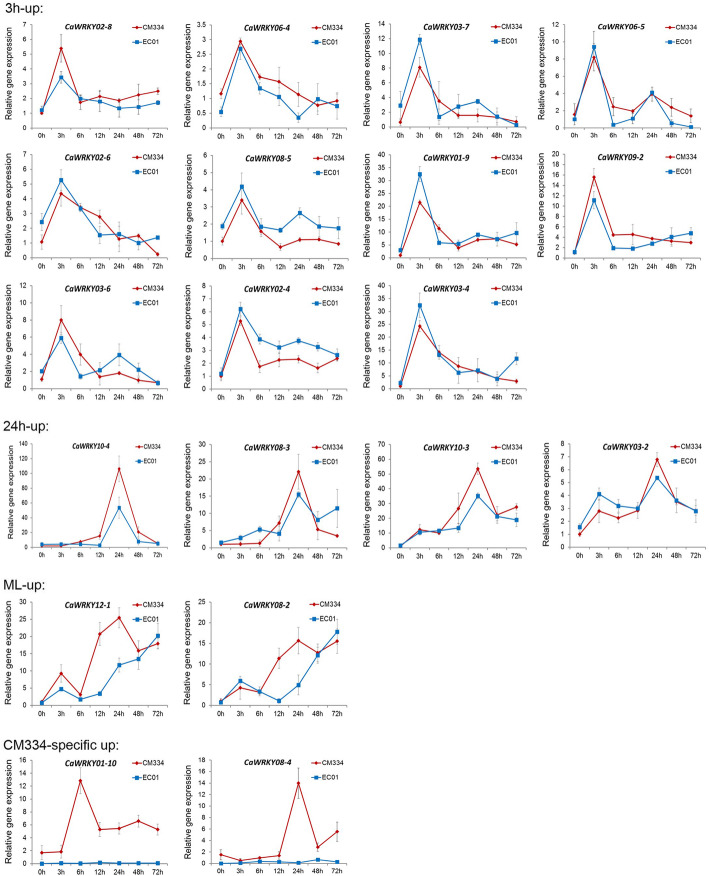
The qRT-PCR verification of the transcriptional profiles of these selected CaWRKY genes in pepper’s response to *P. capsici* infection. Samples were collected at the indicated time points after the pathogen inoculation. Means and standard errors were calculated from three independent biological replicates

**Fig. 7:**
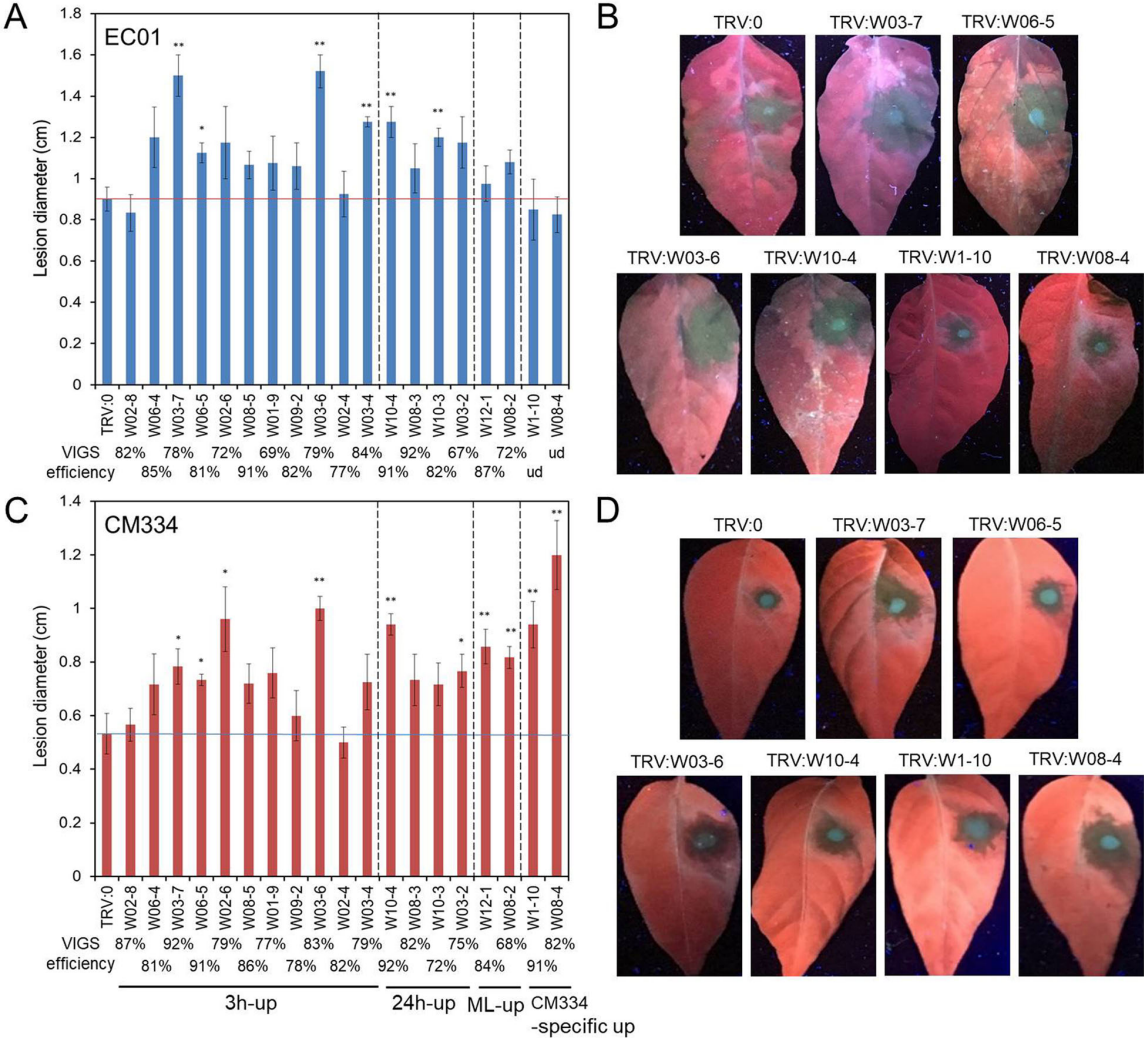
The functional identification of WRKY family members in pepper’s defense against *P. capsici* infection by VIGS system. Nineteen of these induced CaWRKY genes with differential expression patterns were selected for virus-induced gene silencing (VIGS). The VIGS efficiency was determined by qRT-PCR analysis at its highest induction time points after *P. capsici* inoculation in (**a**) EC01 and (**c**) CM334. Expression levels were normalized with *CaActin*, and expressed as mean fold changes relative to TRV:0-treated leaves, which were set as 1. The disease lesions were measured from detached leaves (*n* = 5) at 2.5 days post inoculation from (**b**) EC01 and (**d**) CM334. Asterisks indicate statistically significant differences compared with the TRV:0 empty vector controls by the least significant difference (LSD) test (*P < 0.05; **P < 0.01). This experiment was repeated twice with similar results

To further characterize their contributions in pepper defense against *P. capsici* infection, 19 of these induced CaWRKY genes with differential expression patterns were selected for functional identification. As shown in Fig. 6, all the tested genes validated by qRT-PCR analysis were most consistent with the results of RNA-seq data. We then performed knockdown experiments both in the susceptible line EC01 and the resistant line CM334 using the tobacco rattle virus (TRV) induced gene silencing system [28, 29]. The gene silencing efficiency of each CaWRKY TF was determined by qRT-PCR analysis at its highest induction time points after *P. capsici* inoculation. The results revealed that the silencing efficiency was greater than 65% for all the tested genes in EC01 and CM334, except for *CaWRKY08–4* and *CaWRKY01–10* (Fig. 7a, c and Additional file 6: Figure S2). Owing to up-regulated specifically in CM334 upon *P. capsici* infection, the transcript abundances of *CaWRKY08–4* and *CaWRKY01–10* were too low to detectable in EC01 after gene silencing.

After inoculation with *P. capsici*, the pathogen was restricted to small, localised lesions on TRV:0 treated CM334 leaves (no wilting); whereas, it expanded greatly and displayed much larger lesions on TRV:0 treated EC01 leaves, and most of the inoculated EC01 leaves displayed wilt phenotype within 3 days post inoculation. For these TRV treated leaves targeting the selected CaWRKY genes, we found that most of the overlap induced CaWRKY TFs contribute with various degrees to pepper defense against *P. capsici* (Fig. 7b, d and Additional file 7: Figure S3). Among them, silencing of *CaWRKY03–6*, *CaWRKY03–7*, *CaWRKY06–5* and *CaWRKY10–4* significantly impaired the disease resistance both in CM334 and EC01. While for *CaWRKY08–4* and *CaWRKY01–10*, silencing of them significantly impaired their resistance to the pathogen in CM334 but not in EC01, indicating that these two WRKY genes are prominent modulators specifically in the resistant pepper plants.

## Discussion

It has been suggested that plant defense against pathogen attacks are regulated largely by a complicated transcriptional network [30], and plant TFs might be key modulators in plant immune response [9, 10]. Recently, several WRKY TFs from different plant species have been demonstrated to participate in plant defense against *Phytophthora* spp. [14–23]. However, despite that phytophthora blight frequently cause serious loss in pepper production, our knowledge on the roles of CaWRKY TFs in pepper immunity against *P. capsici* is very limited. In the present study, dynamic profiles of WRKY family genes between the resistant line CM334 and the susceptible line EC01 were comparatively assayed, and these CaWRKY genes up-regulated significantly against *P. capsici* was functionally investigated.

Within the approximately 1665 TFs classified into 58 families in pepper genome [25], a total of 258 and 437 TF DEGs were identified in the present study to be altered in their transcript levels in the resistant line CM334 and susceptible line EC01 upon *P. capsici* infection, respectively (Fig. 1c). The data from enrichment analysis showed that WRKY family was most enriched in both CM334 and EC01 (Fig. 2), implying that WRKY TFs might play more critical roles in modulating the host transcriptional immune against *P. capsici* infection. More in-depth analysis indicated that 36 CaWRKY genes in CM334 (~ 50.0%) and 34 in EC01 (~ 47.2%) were up-regulated, whereas none of them were down-regulated during the defense response (Table 1 and Fig. 1d). Similarly, a previous study showed that 27 of AtWRKY genes from *Arabidopsis* were induced upon flg22 elicitation at 2 h post treatment, while only two AtWRKY genes were down-regulated [31]. Naveed et al. also revealed that most of the TFs from Carrizo citrange, such as ERF, bZIP and DOF, showed mixed trend of up- and down-regulation, whereas most of the WRKY TFs were up-regulated during the *P. parasitica* infection [32]. To our knowledge, there are only two reports about the response of CaWRKYs to *P. capsici* infection so far [24, 26]. Lu et al. selected seven CaWRKY genes (4 of them belong to 3 h-up genes, one is constitutively expressed, one is very low expressed both in CM334 and EC01; and one belong to ML-up genes in EC01 and induced at 6 hpi in CM334 in our RNA-seq data) and identified their expression levels in response to *P. capsici* at 1 day post inoculation by qRT-PCR analysis. This report revealed that six CaWRKY genes were induced after *P. capsici* inoculation, whereas one CaWRKY gene did not display any significant change in CM334. In EC, after *P. capsici* inoculation, one CaWRKY gene was up-regulated, while other six CaWRKY genes remained unchanged or slightly down-regulated. Recently, Zheng et al. indicated that at least 10 CaWRKY genes from pepper cultivar Zunla-1 were induced after *P. capsici* inoculation using the RNA-seq data with four time points (0, 1, 2 and 3 days post inoculation), whereas three CaWRKY genes were down-regulated during the infection. In general, these reports show the similar trend in CaWRKY TFs against *P. capsici* infection with our findings, but also display minor deference and this may be due to use different pepper lines, *P. capsici* strains, and samples collected with different time points. Moreover, our data revealed that the repertoires of these induced CaWRKY genes were highly overlapped between CM334 and EC01. In line with several previous studies [4–6], it indicated that both the resistant and susceptible plants share a large number of WRKY TFs as common signaling components to modulate immune response.

According to their difference in transcription patterns, these induced WRKY genes can be mainly divided into three subgroups: early response (3 h-up), mid response (24 h-up) and mid-late response (ML-up) genes (Fig. 5). This time-order expression patterns suggest that 3 and 24 hpi are two critical time points in the transcriptional reprograming of WRKY family members in pepper’s response to *P. capsici*. In consistent with the pervious researches [11, 33, 34], we speculated that this may be closely related to the pathogenic process of *P. capsici* during the colonization. At 2–4 hpi, the zoospores of *P. capsici* shed their flagella, encyst and adhere to the plant surface; at this time point, PAMPs might be perceived by plant membrane-localized PRRs to trigger PTI or basal defense, leading to the first round of WRKY TFs induction [11, 31]. At ~ 24 hpi, the hyphae can penetrate the plant host cells and form haustorial structure; at this time point, pathogen derived effectors might be delivered into plant host cells and perceived by some lower-evolved intracellular host receptors that trigger defense signaling, leading to the second round of WRKY TFs induction [11, 35, 36]. On further ingress, due to devoid of the corresponding advanced intracellular host receptors (here termed R proteins) in the susceptible host plants, hyphae spread in a large number of susceptible host cells and reached vascular tissue, leading to wilt phenotype. While in the resistant host plants, effectors could be recognized by R proteins, the expansion of hyphae in the epidermal cells is restricted, and the vascular tissue colonization is absent [6, 11, 33, 34], which was in accordance with our observations of the disease phenotypes in CM334 and EC01 inoculated with *P. capsici* (Figs. 1a and 7).

Although a large overlap was found in these induced CaWRKY genes between the resistant and susceptible host plants, there are also some CaWRKY genes with different expression profiles between CM334 and EC01. Comparative transcriptomic analysis indicated that the responses of ML-up CaWRKY genes in the resistant host plants are several hours earlier than those in the susceptible host plants (Fig. 5c and d). In particular, *CaWRKY08–4* and *CaWRKY01–10* were exclusively up-regulated in CM334, while very low expressed in EC01 (Figs. 4 and 6). Our knockdown experiments revealed that most of the overlap induced WRKY TFs contribute to basal defense in the resistant and the susceptible pepper plants against *P. capsici* (Fig. 7). Although some of these induced CaWRKY genes, such as *CaWRKY02–4* and *CaWRKY02–8*, were not significantly contributed to the disease resistance, it could not rule out the possibility of their functional redundancy in the defense signaling. Silencing of *CaWRKY08–4* or *CaWRKY01–10* significantly impaired its resistance to the pathogen in CM334 but not in EC01, indicating that these two CaWRKY genes are prominent modulators specifically in the resistant pepper plants. In fact, some pathogens could deliver effectors to target multiple defense-promoting WRKY TFs, causing loss of WRKY-DNA binding and trans-activating functions needed for defense gene expression and disease resistance [37–39]. Whether *Phytophthora* uses this particular strategy to inhibit the transcripts or functions of WRKY TFs (such as CaWRKY08–4 and CaWRKY01–10) in the susceptible host plants, is required to further investigate. It is noted that the large numbers of TFs including WRKYs in response to pathogen infection might act in a complex regulatory network rather than in a linear manner [31, 40, 41]. Further identification of their upstream signaling components (such as cis-elements and trans-factors) and downstream target genes might provide new insights into the molecular mechanism underlying pepper resistance to *P. capsici*.

## Conclusions

In this study, we performed time-series RNA-seq from the resistant and susceptible pepper plants upon challenged with *P. capsici*, and revealed their transcriptional similarities and differences of WRKY family members in response to the pathogen. We also performed knockdown experiments by VIGS to functionally investigate their roles in disease resistance. Collectively, the data presented here considerably extend our understanding of WRKY family members in plant defense response, and also provide potential applications for genetic improvement against phytophthora blight.

## Methods

### Plant materials, pathogen and culture conditions

The seeds of *P. capsici*-resistant pepper landrace line ‘Criollo de Morelos 334’ (CM334) and susceptible cultivar ‘Early Calwonder 01’ (EC01) were sown in a soil mix [peat moss: perlite, 2:1 (v/v)] in plastic pots, and were placed in a growth room under a condition of 25 °C, 60–70 mmol photons m^− 2^ s^− 1^, a relative humidity of 70%, and a 16-h light/8-h dark photoperiod [29]. A highly virulent *P. capsici* stain JX1 was isolated by our laboratory and cultured as described previously [42]. Briefly, the *P. capsici* stain was cultured on 10% (v/v) V8 agar medium, and then transferred to 10% (v/v) V8 liquid medium for 3 days at 25 °C in the dark. The mycelia of *P. capsici* were washed intermittently with sterilized H_2_O for three times to induce zoospore release [42].

### Transcriptome analysis

To obtain RNA-seq data from *P. capsici*-infected pepper tissues, four-week-old (at 5 true leaf stage) soilless cultivated pepper lines CM334 and EC01 were grown with a Holland solution under a 16-h light/8-h dark cycle at 25 °C prior to inoculation. The zoospores were counted using a hemocytometer and their density was adjusted to approximately 5 × 10^5^ zoospores/mL. Pepper roots were immersed with the zoospore suspension to ensure that the root surface can be adhered by enough zoospores, and then respectively harvested at 0, 3, 6, 12, 24, 48, 72 h after pathogen inoculation. RNA samples extracted from three biological replicates of each treatment were used for library construction. These constructed libraries (PE150) were then sequenced by the Illumina HiSeq2000 (Illumina Inc., San Diego, USA). After filtered with low-quality reads by BBTools (https://jgi.doe.gov/data-and-tools/bbtools), the clean reads were then aligned to the pepper CM334 genome version PEP (v1.6) (http://peppergenome.snu.ac.kr/) using HISAT2 program (https://github.com/DaehwanKimLab/hisat2). Following alignment to each gene model, we normalized the number of mapped reads to FPKM. The Z-scores of RNA-seq data sets were used for analysis of gene expression patterns using the ggplot2 package (https://cran.r-project.org/web/packages/ggplot2/) and construction of heat maps using the pheatmap package (https://cran.r-project.org/web/packages/pheatmap/). Enrichment analysis was performed by ggplot2 package (http://had.co.nz/ggplot2/).

### Identification of CaWRKY family members

The pepper annotated genome and protein sequences were downloaded from the CM334 genome version PEP (v1.6) (http://peppergenome.snu.ac.kr/). The WRKY domain (PF03106) was obtained from PFAM database (http://pfam.sanger.ac.uk/), and was used to identify putative CaWRKY proteins by HMMER 3.0 software program (http://hmmer.janelia.org/) according to the HMMR User’s Guide. The non-redundant CaWRKY protein sequences were further confirmed using SMART program (http://smart.embl-heidelberg.de/).

### Chromosomal location and phylogenetic tree analysis

All identified CaWRKYs were mapped to the ‘CM334’ chromosomes in the pepper genome database using BLASTn. The MapInspect software (http://mapinspect.software.informer.com/) was used to map the gene locus on chromosomes. *Arabidopsis thaliana* WRKY (AtWRKY) protein sequences were downloaded from TAIR (https://www.arabidopsis.org/browse/genefamily/WRKY.jsp). We performed a multiple alignment of the 72 CaWRKY and 71 AtWRKY full-length protein sequences using ClustalX2 (http://www.clustal.org/clustal2/). The alignment result was used to construct a phylogenetic tree using the neighbor-joining method of PhyML software (http://www.atgc-montpellier.fr/phyml/).

### RNA extraction and qRT-PCR analysis

Total RNA was extracted from *P. capsici*-infected pepper tissues at the indicated time points as described previously [42]. In brief, total RNA was extracted by a PureLink RNA mini kit (Invitrogen, Carlsbad, CA, USA) and treated with RNase-free DNase I (Takara Bio, Kusatsu, Japan). Then, the first-strand cDNA was reversely transcribed by Superscript II reverse transcriptase (Invitrogen). To determine the relative transcription levels of selected genes, real-time PCR was performed with specific primers (Additional file 8: Table S1) according to the manufacturer’s instructions for the BIO-RAD Real-time PCR system (Foster City, CA, USA) and the SYBR Premix Ex Taq II system (TaKaRa). Three independent biological replicates of each treatment were performed. The data were analyzed using the Livak method and calculated as a normalized relative expression level (2^-ΔΔCT^) [43]. The pepper housekeeping gene *CaActin* was served as an endogenous control [44].

### The VIGS vectors construction

To construct virus-induced gene silencing (VIGS) vectors of the selected CaWRKY genes, each of the specific silencing fragment was determined by BLAST analysis using the VIGS tool in the Sol Genomic Network (SGN) website [45], and no off-target gene (which share no more than 19 bp matching fragment) was detected in the pepper genome cDNA database. The silencing fragments were amplified by PCR from cDNA of CM334 using PrimeSTAR GXL DNA Polymerase (Takara Bio Inc., Otsu, Japan) with the primers listed in Additional file 8: Table S1. The purified fragments were cloned into the entry vector pDONR207, and then cloned into the TRV silencing vector pTRV2 by Gateway® technology (Invitrogen).

### VIGS of CaWRKY genes in pepper plants

For silencing of each CaWRKY gene in pepper plants, we employed the TRV-based VIGS system according to our previous studies [28, 29]. Briefly, the *A. tumefaciens* strains GV3101 containing pTRV1 and pTRV2: *CaWRKY* were resuspended in the induction medium (OD600 = 0.8) and mixed thoroughly at 1:1 (v/v) ratio, and then infiltrated into the two cotyledons of 2-week-old pepper plants. pTRV2:*0* (empty vector) was severed as a negative control. pTRV2: *CaPDS*, which silences pepper phytoene desaturase (PDS) gene to induce photobleaching phenotype, was used as an indicator control (Additional file 9: Figure S4). The agro-infiltrated pepper plants were kept in an incubator in darkness at 16 °C for 56 h, and then grown in the growth room under normal conditions as described above for 3–4 weeks [28, 29, 46].

### *P. capsici* infection assays

For infection assays, the third and fourth detached leaves from the top of each TRV treated pepper plant were inoculated with the highly virulent *P. capsici* stain JX1 zoospores, respectively [42]. To distinguish the disease phenotypes among different VIGS plants, especially for the susceptible pepper line EC01, each detached leaf was inoculated with ~ 100 *P. capsici* zoospores (a relative low concentration) under low disease-pressure conditions. After *P. capsici* inoculation, the VIGS efficiency was determined by qRT-PCR analysis using the fourth detached leaves at the highest induction time points of each CaWRKY gene, and the third detached leaves were kept at high humidity in the dark at 25 °C for 2–3 days prior to measure disease lesions. This experiment was repeated twice, each time with five replicates.

## Supplementary information

**Additional file 1: Dataset S1.** Summary of RNA-seq libraries of pepper samples infected with *P. capsici*.

**Additional file 2: Figure S1.** Supplementary enrichment analysis of TF DEGs between CM334 and EC01 after *P. capsici* infection**.** (A) Enrichment analysis was performed using all identified TF DEGs in CM334 and EC01. (B) Enrichment analysis was performed using the down-regulated TF DEGs in the two lines. The enrichment analysis was performed by ggplot2 package (http://had.co.nz/ggplot2/).

**Additional file 3 Dataset S2.** The characterization of CaWRKY family members.

**Additional file 4: Dataset S3.** The nucleotide and protein sequences of CaWRKY family members.

**Additional file 5: Dataset S4.** The classification of WRKY family members in pepper’s response to *P. capsici* infection.

**Additional file 6: Figure S2.** Summary of silencing efficiency of the selected CaWRKY genes in VIGS pepper plants. The VIGS efficiency was determined by qRT-PCR analysis at its highest induction time points after *P. capsici* inoculation in (A) EC01 and (B) CM334. The expression levels were normalized with *CaActin*, and expressed as mean fold changes relative to TRV:0-treated leaves, which were set as 1.

**Additional file 7: Figure S3.** Phenotypes of representative detached leaves from other *CaWRKYs*-silenced pepper plants. Disease symptoms of detached leaves in pepper lines (A) EC01 and (B) CM334 at 2.5 days post-inoculation with *P. capsici*.

**Additional file 8: Table S1.** Primers used in this study.

**Additional file 9: Figure S4.** A *PDS*-silenced control in the VIGS experiment.

## Data Availability

All data generated in this study are included in the paper and in the supporting information files. The RNA-Seq data used in the current study are available in the NCBI Sequence Read Archive (SRA) under accession number: PRJNA627546 (https://www.ncbi.nlm.nih.gov/sra/PRJNA627546).

## Abbreviations

DEGs: Differentially expressed genes
FDR: False discovery rate
FPKM: Fragments per kilobase million
hpi: Hours post inoculation
qRT-PCR: Quantitative real-time polymerase chain reaction
TFs: Transcription factors
TRV: Tobacco rattle virus
VIGS: Virus-induced gene silencing

## References

[1] Jones JD, Dangl JL (2006). The plant immune system. Nature..

[2] Dangl JL, Horvath DM, Staskawicz BJ (2013). Pivoting the plant immune system from dissection to deployment. Science..

[3] Bhattarai KK, Atamian HS, Kaloshian I, Eulgem T (2010). WRKY72-type transcription factors contribute to basal immunity in tomato and Arabidopsis as well as gene-for-gene resistance mediated by the tomato R gene Mi-1. Plant J.

[4] Tsuda K, Somssich IE (2015). Transcriptional networks in plant immunity. New Phytol.

[5] Tsuda K, Katagiri F (2010). Comparing signaling mechanisms engaged in pattern-triggered and effector-triggered immunity. Curr Opin Plant Biol.

[6] Buscaill P, Rivas S (2014). Transcriptional control of plant defence responses. Curr Opin Plant Biol.

[7] Peng YJ, van Wersch R, Zhang YL (2018). Convergent and divergent signaling in PAMP-triggered immunity and effector-triggered immunity. Mol Plant Microbe In.

[8] Garner CM, Kim SH, Spears BJ, Gassmann W (2016). Express yourself: transcriptional regulation of plant innate immunity. Semin Cell Dev Biol.

[9] Birkenbihl RP, Liu S, Somssich IE (2017). Transcriptional events defining plant immune responses. Curr Opin Plant Biol.

[10] Chen F, Hu Y, Vannozzi A, Wu KC, Cai HY, Qin Y, Mullis A, Lin ZG, Zhang LS (2017). The WRKY transcription factor family in model plants and crops. Crit Rev Plant Sci.

[11] Lamour KH, Stam R, Jupe J, Huitema E (2012). The oomycete broad-host-range pathogen Phytophthora capsici. Mol Plant Pathol.

[12] Kamoun S, Furzer O, Jones JDG, Judelson HS, Ali GS, Dalio RJD, Roy SG, Schena L, Zambounis A, Panabieres F (2015). The top 10 oomycete pathogens in molecular plant pathology. Mol Plant Pathol.

[13] Babadoost M, Pavon C, Islam SZ, Tian D (2015). Phytophthora blight (Phytophthora capsici) of pepper and its management. Acta Hortic.

[14] Adachi H, Nakano T, Miyagawa N, Ishihama N, Yoshioka M, Katou Y, Yaeno T, Shirasu K, Yoshioka H (2015). WRKY transcription factors phosphorylated by MAPK regulate a plant immune NADPH oxidase in Nicotiana benthamiana. Plant Cell.

[15] Fan S, Dong L, Han D, Zhang F, Wu J, Jiang L, Cheng Q, Li R, Lu W, Meng F (2017). GmWRKY31 and GmHDL56 enhances resistance to Phytophthora sojae by regulating defense-related gene expression in soybean. Front Plant Sci.

[16] Cui X, Yan Q, Gan S, Xue D, Wang H, Xing H, Zhao J, Guo N (2019). GmWRKY40, a member of the WRKY transcription factor genes identified from *Glycine max* L., enhanced the resistance to Phytophthora sojae. BMC Plant Biol.

[17] Yogendra KN, Kumar A, Sarkar K, Li Y, Pushpa D, Mosa KA, Duggavathi R, Kushalappa AC (2015). Transcription factor StWRKY1 regulates phenylpropanoid metabolites conferring late blight resistance in potato. J Exp Bot.

[18] Yogendra KN, Dhokane D, Kushalappa AC, Sarmiento F, Rodriguez E, Mosquera T (2017). StWRKY8 transcription factor regulates benzylisoquinoline alkaloid pathway in potato conferring resistance to late blight. Plant Sci.

[19] Cui J, Xu P, Meng J, Li J, Jiang N, Luan Y (2018). Transcriptome signatures of tomato leaf induced by Phytophthora infestans and functional identification of transcription factor SpWRKY3. Theor Appl Genet.

[20] J-b L, Y-s L, Liu Z (2015). SpWRKY1 mediates resistance to Phytophthora infestans and tolerance to salt and drought stress by modulating reactive oxygen species homeostasis and expression of defense-related genes in tomato. Plant Cell Tiss Org.

[21] J-b L, Y-s L, Liu Z (2015). Overexpression of SpWRKY1 promotes resistance to Phytophthora nicotianae and tolerance to salt and drought stress in transgenic tobacco. Physiol Plantarum.

[22] Cui J, Jiang N, Meng J, Yang G, Liu W, Zhou X, Ma N, Hou X, Luan Y (2019). LncRNA33732-respiratory burst oxidase module associated with WRKY1 in tomato-Phytophthora infestans interactions. Plant J.

[23] Hong Y, Cui J, Liu Z, Luan Y (2018). SpWRKY6 acts as a positive regulator during tomato resistance to Phytophthora infestans infection. Biochem Bioph Res Co.

[24] Lu J, Guo M, Zhai Y, Gong Z, Lu M (2017). Differential responses to the combined stress of heat and Phytophthora capsici infection between resistant and susceptible germplasms of pepper (Capsicum annuum L.). J Plant Growth Regul.

[25] Tian F, Yang DC, Meng YQ, Jin J, Gao G (2020). PlantRegMap: charting functional regulatory maps in plants. Nucleic Acids Res.

[26] Zheng JY, Liu F, Zhu CH, Li XF, Dai XZ, Yang BZ, Zou XX, Ma YQ (2019). Identification, expression, alternative splicing and functional analysis of pepper WRKY gene family in response to biotic and abiotic stresses. PLoS One.

[27] Diao WP, Wang SB, Liu JB, Pan BG, Guo GJ, Ge W (2015). Genome-wide analysis of the WRKY transcription factor family in pepper. Acta Hortic Sin.

[28] Dang FF, Wang YN, Yu L, Eulgem T, Lai Y, Liu ZQ, Wang X, Qiu AL, Zhang TX, Lin J (2013). CaWRKY40, a WRKY protein of pepper, plays an important role in the regulation of tolerance to heat stress and resistance to Ralstonia solanacearum infection. Plant Cell Environ.

[29] Cheng W, Xiao ZL, Cai HY, Wang CQ, Hu Y, Xiao YP, Zheng YX, Shen L, Yang S, Liu ZQ (2017). A novel leucine-rich repeat protein, CaLRR51, acts as a positive regulator in the response of pepper to Ralstonia solanacearum infection. Mol Plant Pathol.

[30] Tsuda K, Sato M, Stoddard T, Glazebrook J, Katagiri F (2009). Network properties of robust immunity in plants. PLoS Genet.

[31] Birkenbihl RP, Kracher B, Ross A, Kramer K, Finkemeier I, Somssich IE (2018). Principles and characteristics of the Arabidopsis WRKY regulatory network during early MAMP-triggered immunity. Plant J.

[32] Naveed ZA, Huguet-Tapia JC, Ali GS (2019). Transcriptome profile of Carrizo citrange roots in response to Phytophthora parasitica infection. J Plant Interact.

[33] Kebdani N, Pieuchot L, Deleury E, Panabieres F, Le Berre JY, Gourgues M (2010). Cellular and molecular characterization of Phytophthora parasitica appressorium-mediated penetration. New Phytol.

[34] Jupe J, Stam R, Howden AJM, Morris JA, Zhang RX, Hedley PE, Huitema E (2013). Phytophthora capsici-tomato interaction features dramatic shifts in gene expression associated with a hemi-biotrophic lifestyle. Genome Biol.

[35] Chen XR, Xing YP, Li YP, Tong YH, Xu JY (2013). RNA-Seq reveals infection-related gene expression changes in Phytophthora capsici. PLoS One.

[36] Stam R, Jupe J, Howden AJM, Morris JA, Boevink PC, Hedley PE, Huitema E (2013). Identification and characterisation CRN effectors in Phytophthora capsici shows modularity and functional diversity. PLoS One.

[37] Shen QH, Saijo Y, Mauch S, Biskup C, Bieri S, Keller B, Seki H, Ulker B, Somssich IE, Schulze-Lefert P (2007). Nuclear activity of MLA immune receptors links isolate-specific and basal disease-resistance responses. Science..

[38] Le Roux C, Huet G, Jauneau A, Camborde L, Tremousaygue D, Kraut A, Zhou B, Levaillant M, Adachi H, Yoshioka H (2015). A receptor pair with an integrated decoy converts pathogen disabling of transcription factors to immunity. Cell..

[39] Sarris PF, Duxbury Z, Huh SU, Ma Y, Segonzac C, Sklenar J, Derbyshire P, Cevik V, Rallapalli G, Saucet SB (2015). A plant immune receptor detects pathogen effectors that target WRKY transcription factors. Cell..

[40] Eulgem T, Somssich IE (2007). Networks of WRKY transcription factors in defense signaling. Curr Opin Plant Biol.

[41] Berri S, Abbruscato P, Faivre-Rampant O, Brasileiro AC, Fumasoni I, Satoh K, Kikuchi S, Mizzi L, Morandini P, Pe ME (2009). Characterization of WRKY co-regulatory networks in rice and Arabidopsis. BMC Plant Biol.

[42] Cheng W, Lin ML, Qiu M, Kong L, Xu YP, Li YN, Wang Y, Ye WW, Dong SM, He SL (2019). Chitin synthase is involved in vegetative growth, asexual reproduction and pathogenesis of Phytophthora capsici and Phytophthora sojae. Environ Microbiol.

[43] Livak KJ, Schmittgen TD (2001). Analysis of relative gene expression data using real-time quantitative PCR and the 2(−Delta Delta C(T)) method. Methods..

[44] Wang SB, Liu KW, Diao WP, Zhi L, Chen JF (2012). Evaluation of appropriate reference genes for gene expression studies in pepper by quantitative real-time PCR. Mol Breeding.

[45] Fernandez-Pozo N, Rosli HG, Martin GB, Mueller LA (2015). The SGN VIGS tool: user-friendly software to design virus-induced gene silencing (VIGS) constructs for functional genomics. Mol Plant.

[46] Senthil-Kumar M, Mysore KS (2014). Tobacco rattle virus-based virus-induced gene silencing in Nicotiana benthamiana. Nat Protoc.

