# Supplementary figures and images for: The transcriptional reprograming and functional identification of WRKY family members in pepper’s response to Phytophthora capsici infection
Source: BMC Plant Biol. 2020 Jun 3;20:256. doi: 10.1186/s12870-020-02464-7 (PMC7271409; doi:10.1186/s12870-020-02464-7)

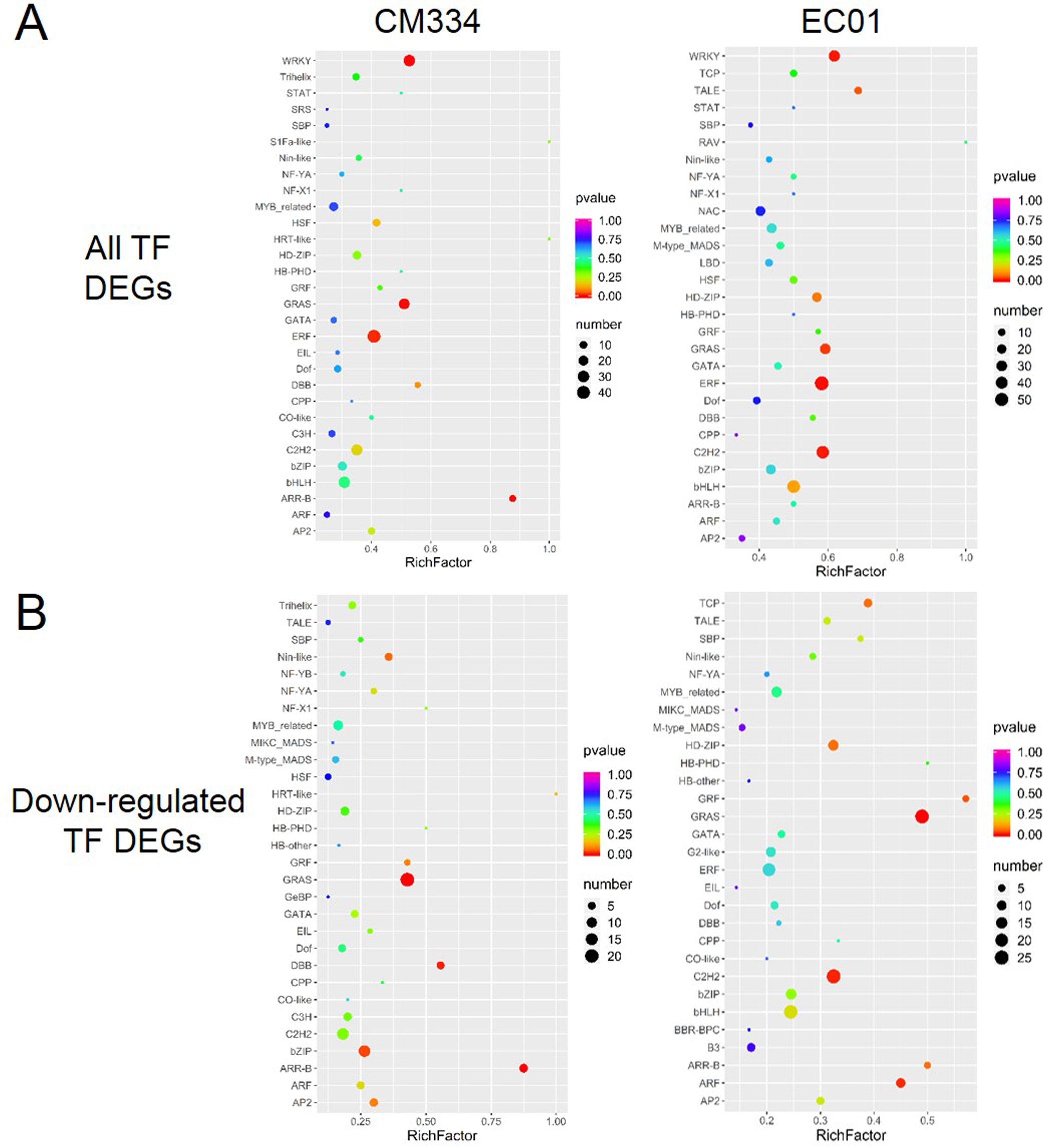

Supplement: Supplementary file 2 — Additional file 2: Figure S1. Supplementary enrichment analysis of TF DEGs between CM334 and EC01 after P. capsici infection. (A) Enrichment analysis was performed using all identified TF DEGs in CM334 and EC01. (B) Enrichment analysis was performed using the down-regulated TF DEGs in the two lines. The enrichment analysis was performed by ggplot2 package (http://had.co.nz/ggplot2/). [file 12870_2020_2464_MOESM2_ESM.tif]

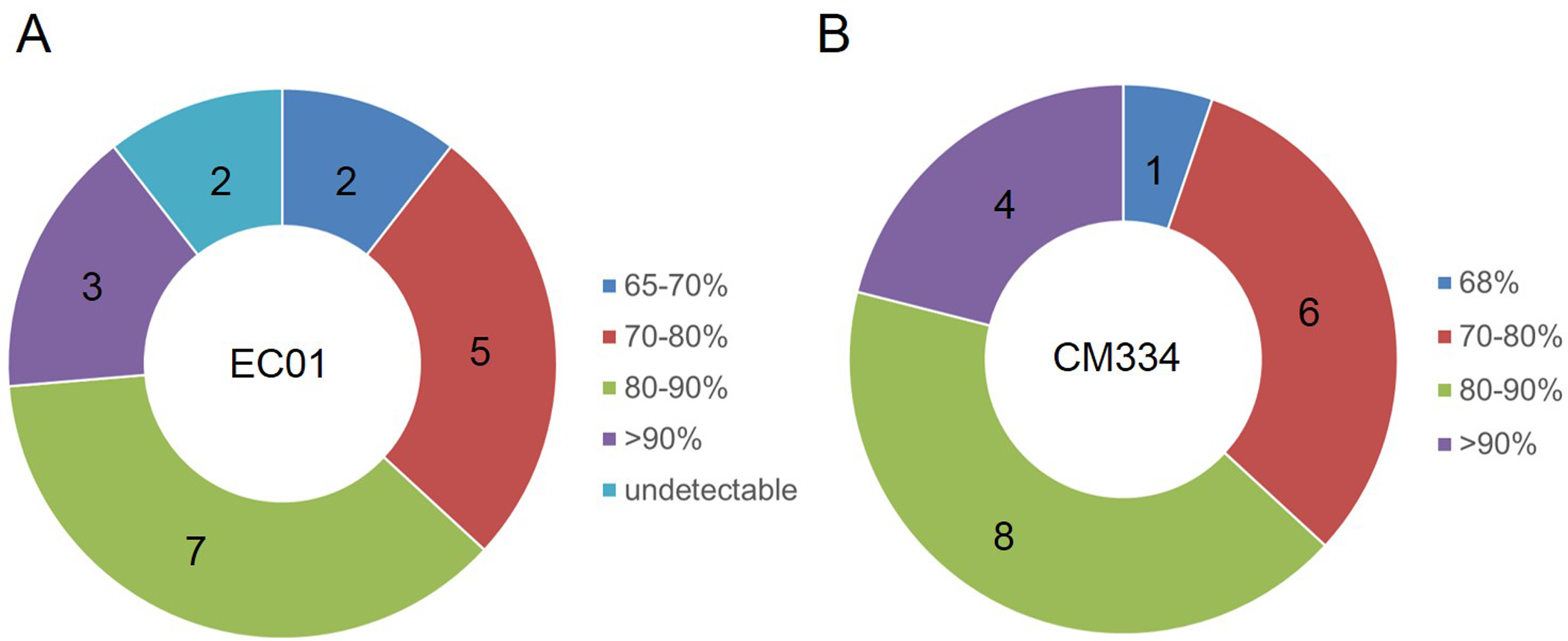

Supplement: Supplementary file 6 — Additional file 6: Figure S2. Summary of silencing efficiency of the selected CaWRKY genes in VIGS pepper plants. The VIGS efficiency was determined by qRT-PCR analysis at its highest induction time points after P. capsici inoculation in (A) EC01 and (B) CM334. The expression levels were normalized with CaActin, and expressed as mean fold changes relative to TRV:0-treated leaves, which were set as 1. [file 12870_2020_2464_MOESM6_ESM.tif]

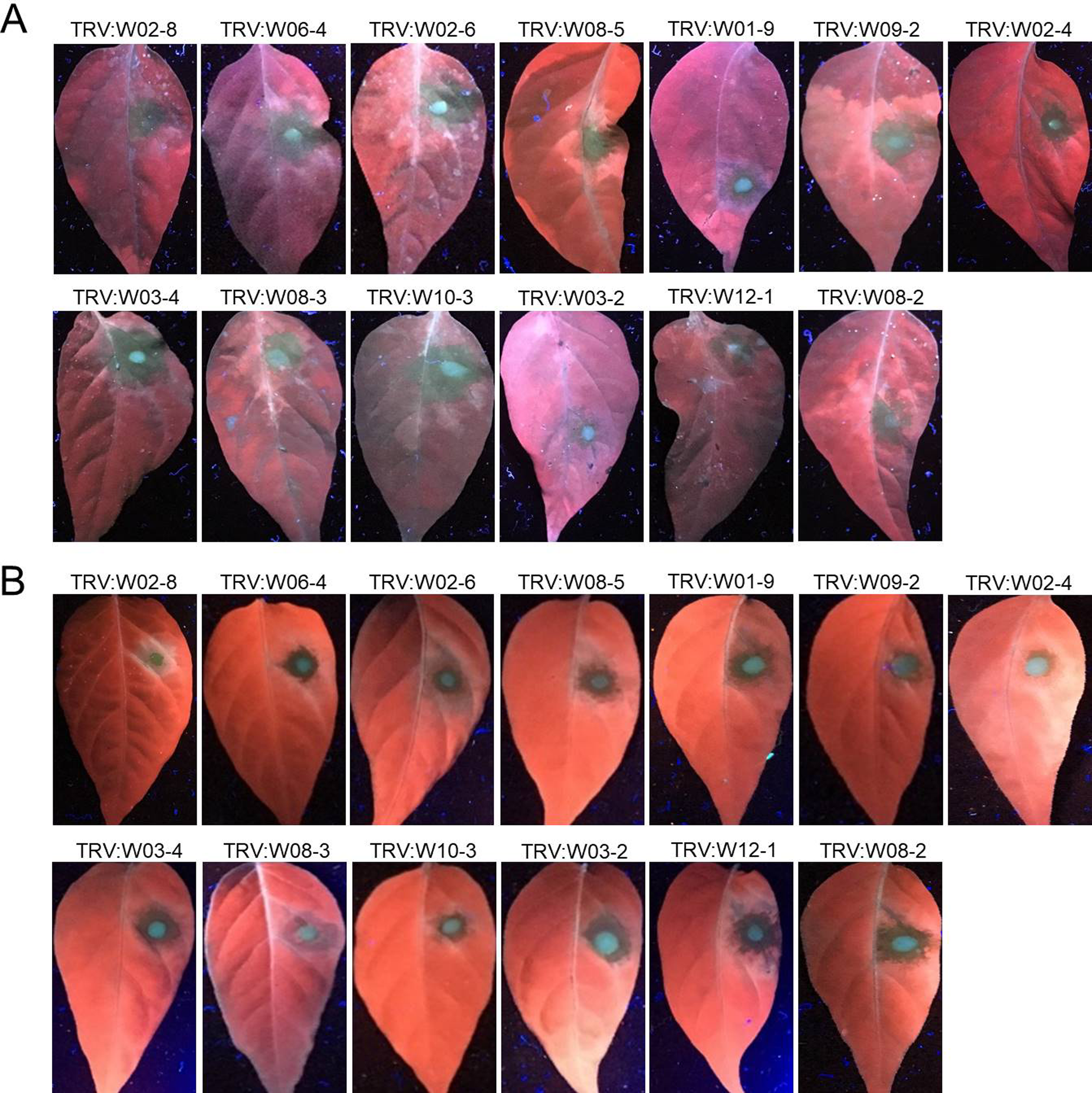

Supplement: Supplementary file 7 — Additional file 7: Figure S3. Phenotypes of representative detached leaves from other CaWRKYs-silenced pepper plants. Disease symptoms of detached leaves in pepper lines (A) EC01 and (B) CM334 at 2.5 days post-inoculation with P. capsici. [file 12870_2020_2464_MOESM7_ESM.tif]

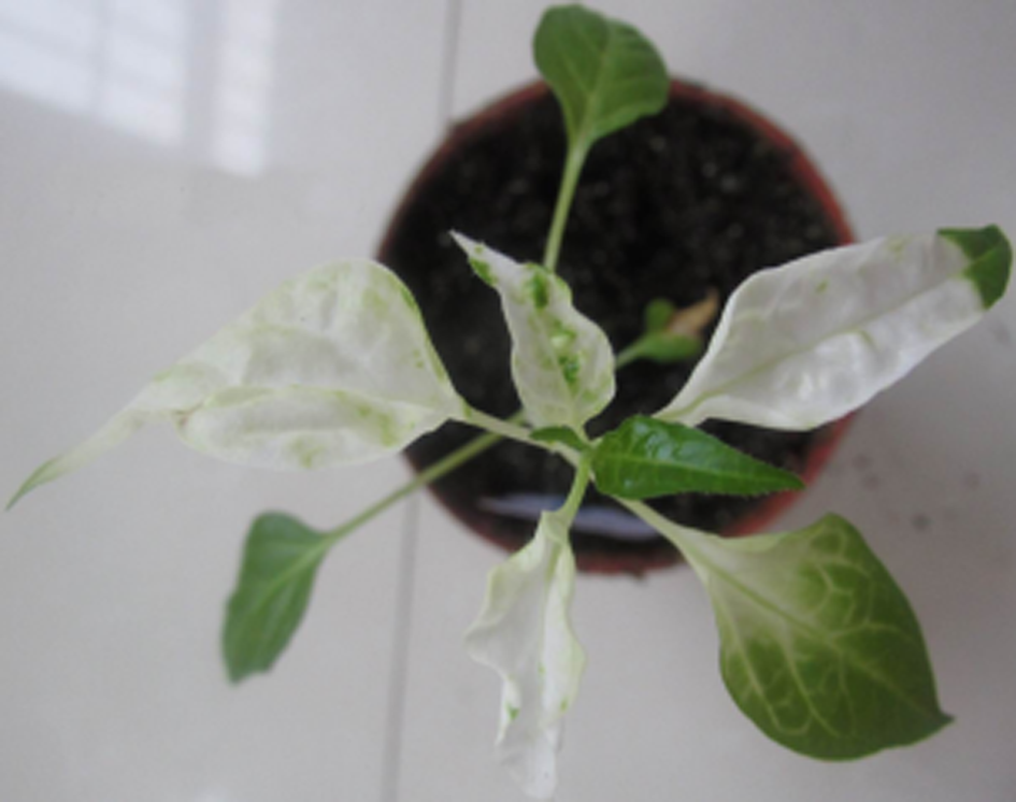

Supplement: Supplementary file 9 — Additional file 9: Figure S4. A PDS-silenced control in the VIGS experiment. [file 12870_2020_2464_MOESM9_ESM.tif]
