# Supplementary material for: The transcriptional reprograming and functional identification of WRKY family members in pepper’s response to Phytophthora capsici infection
Source: BMC Plant Biol. 2020 Jun 3;20:256. doi: 10.1186/s12870-020-02464-7 (PMC7271409; doi:10.1186/s12870-020-02464-7)
Supplement: Supplementary file 4 — Additional file 4: Dataset S3. The nucleotide and protein sequences of CaWRKY family members. [file 12870_2020_2464_MOESM4_ESM.docx]

**The nucleotide and protein sequences of CaWRKY family members**

> CaWRKY01-1

ATGGATTGTGGATTTAATTGGGAGTCTAATTCATTGATAAATGAATTAACACAAGGATTAGAACATGCTAAGCAATTAAGAGCTTCTTTTTCTTATGATAAAATTCAAGAATTGGATTTTCATTTGCAAATGATTTTTTCTTCTTTTGAAAAATCCTTGTCAATTCTCAAATGGAATGATACAGTAACACAAAGTCCATTACTTATAGCACCACTAGTATCAACTGGTGCACCAGAATCTTCAATTTCTGTTGATGACAACAATAACAATAACGTATCTAGTGAAATTCCACACGTGGGGTATGGAGAGGGTACAGTGTACGCTGACGTTACCCCTAAAATGGATGATCAAGATTTCAAATATGTTTCAAAGAAGAGAAAACAGATGCCTACATGGAGTGAACAAGTGAGAGTTAATGCAGAGAATGGATATGAAAGGCCTACTGATGATGGATTTAGTTGGAGAAAATATGGACAAAAAGATATTCTTGGTGCTAAATATCCCAGGAGTTACTACAGATGCACCTATAGACTAATGCAGAATTGTTGGGCAACAAAGCAAGTGCAAAGGTCTGATGATGATCCTGCACTATTTGAGATCACATACAAAGGGTCACATACTTGTAACCAAACTTTTCACTCTGCTACACAACTAAAATCACCAGAGAAACATAAACTCAAGAAACAAGCCAACAATCCAAGAACAATACAATCGAACCAAATGCTCGCAAACTTTCAAGCAAATTTGAGAGTGAATATAAATGATTTGGACAAGAAAGAAGCAACAAGTTCCTTCCAATTTTCACCTACATTCTCTGGGTTCGTAAGCGAAAATCTGCATTTTCAGAGGTCACAAGATGATGATAATTTGGTTGGGGGATATTCACTATCTTTTGTCTCTCCTACAACCCCTGAATCAAGTTACTTCTCATTACCAAGCTCCCATATGAATGATTCGCGGAGAATTCACAATGTGCACCATTCAGAATCAGACCTCCCTGATTTGTTCTCAGCCAACACTTCATCAACAAGTTCTCCAATTATAGGCTTGGAGTTTCCACTTGAACGTGTGGAGCTAGATCCAAATTTTCCATTTCATAACTCAGAATTTTTCAGATAA

> CaWRKY01-2

ATGCAACAACCTAATGATCAAATGCCCATGCAAAATAATTATCACTCACACGTTGGACTTGATTGTGATAATATTGATTGGGCCGGGCTTTTATCGGCGGGCCCTTCAATTAATAATGAGTCGATTGCAACTACGTCAAATGTATCGATAAATCATAGGAATATGAATACAAATATGATGGAAGGTGGTGAACAACATCAACAACAATTATTACCACAACAACAACAACATGAAGTATGTAGAAGAGATAAAGGGAGAATAATAAAGAAGAGAAAATATGTACCACCTAGGATTGCATTTCATACAAGGAGTACTGAGGATATTCTTGATGATGGATTTAAGTGGAGAAAATATGGTCAAAAAGCTGTCAAAAATAGTACACATCCAAGGAGTTACTATCGATGTACACATCACACATGCAATGTGAAAAAACAAATTCAAAGACATTCAAAGGATACAAGTATTGTGGTGACAACTTATGAAGGCATTCACAATCACCCTTGTGAGAAACTAATGGAGACCTTAAGTCCAATTCTAAAGCAACTTCAGTTTCTCTCTAGGTTCTAA

> CaWRKY01-3

ATGATGATAATGGATTGGGGTCTTCAAGCTGTTGTTATTGGATCATCAAGTACTTCATGTAGTGATATTCATGGATCAAATTATGTTGATTTTTCACCTAATTTGGATTTTCAAGAAAGTGAATATTTATCTTTTTCTCATCATCATCATCAACATCATGAGATGAAAAAAGAGGTTTATAGTGATGAATTAGAACAACTTTACAAGCCATTTTATCATGTTGGTGGACAAAATATGTTGATGGGATCTTCAATTTCATTAGTTCCCAAAGAAGTTATTAAAGAAGAGAAAAGAGAAGAAGAGCAACAACAAGTGGTGGCTGCTAGCAATACATATGTACCCAAGTACAAAAAAAGGAAAAATGAACAAAAGAGAGTGGTGCTTCAGTTGAAAGCAGATGATCTTTCTTCTGATAAGTGGGCATGGAGAAAATATGGTCAAAAACCTATTAAAGGCTCCCCTTATCCAAGGAGTTATTATAGGTGCAGTAGCTCAAAGGGGTGTTTAGCAAGAAAACAAGTAGAACAAAGTTGTACTGAACATGGAATTTTTATTGTGACATACACAGCTGAACACAACCATAGTCAACCAACACGTAGAAATTCACTAGCTGGTACTACCAAAAGCAAATTTCCAAATTCAAAAAACTCCATTAATAGTCCCAAGAAGATTGTGAAAGAAGAAAAAATTACAAGTCCACATTGTTCAACATCGAATTTAGGGTTATCTCCAGAAGCAGCACGTATGATCGACGAATTTCCAGAAATAAATCAAGAGAATAATATTATTATAGGTGGTGACAATAATTATTATGATGAAGATGGGATGAGAAATATTTTTGAAGGAAGTGATGAAAATGAATGTGTTTCAATTGAAGAAATGTTTGATGGAGATTTCTTTGCTGGTCTTGAAGATATTCATGATGGATTTAATTCATCTTTTGGATGTAATAATTCAGCATTTCCCTTTTCTAGTACTTGA

> CaWRKY01-4

ATGTTTGTCTATGAATTATTTTCAAGTCTTCAAAAAGTGGCTCATACCAGTTTTTGTGACTTTCACTTACCAACTTCAAATATTTTTCAAGTAAAATGCATATCTAGAAGTTTTAAGTTTCAACTTCAAAATTTATCGGCAAATGGAGCATATATTTGTAGTTTGGTCATTGTTTTTTGTTTCCATACACAGAGATATGTGAGCATGAAAAGGGGACTTTTTTCTGTTTTTTCTGACAGCTCTGAGTGTCCTGATATTAGGAAGATTGGTCAGAAAGTTGTGTTCAAAGTGCAAATGGAAGGAAAAGCTATAAAGCAGAAAAATGAAGGGCCTCCTTCAGATTGTTGGTCCTGGAGAAAATATGGACAGAAACCCATTAAAGGTTCACCTTATCCCAGGGGATACTACAGATGCAGCAGTTCAAAGGGTTGTTCAGCTAAAAAACAAGTAGAAAGGTGCAGTAAAGATGCATCCTTGTTCATCATCACATACACATCCAGCCATAATCATCCAGGTCCAAACTTGCCTAATAATAAAGACACAGTCACACATGATTCCACCGCGACTCTGCCGCAGCAAGATCGAGAACCAACAGTGGATAAAGACGTGCCCCTCAAAGATAATGGGGATAGTACTACTACTACTACTACTGCCACCAGCATCTCCCAAGGCATACCTGAAGAGAATTTGTTCACTGATAGTTTCTTGGGAACAATTTCATATGATGATTTTCTGCCCCTTTCTTACCCTCAACTAATGGAATTCCCAAAATCCGAATTGTCAGAAGAAAATGACTTCTATGATGAACTGGGAGAATTGCAACTACCTCCATCTTCTACGTCCTTCGCAGGCATTTTTGAGGAGGCAATCCTTGTAGATCCCTCTTAG

> CaWRKY01-5

ATGGAAAGCTACAAAGAGATAAAAATTGAAGATCATCCAATGTATTACCTTGATAATAATTTTGCAGTCACTAATAGCCATTCATTTACAGGCCTAATCTCAGATTATTATGGTGTTGAAGGTAGGAATATTATGAATACATCGTCTTCTTTGGGGTTCATGGAGTTATTGGGTTTTCAAGATTTGATGTGTTCATCATCAGCTTCATTCTTTGAGTTACCAAAAGAAGAAAACTCCTGTCCTGCAGTTTGTGTATCTGAAGAAGTGAAGCCAACTGCAGGTGAAAGCCAAAATAAGCTTATAAGTACTGTAGCAGCAGCTAACGTATTCAATACGCCATCTACCCCAAACTGTTCCTCTATTTCATCCGAGACAAATGAGGGCCACACTAATACCACTCATGAGGATGCAGAGGCCGGGGAAGTACTAGATCATCAGGACCAACAACACACCAACACGAAACAACAGTTGAAAGCGAAGAAAACAGTTAGTCAGAAGAAGCAGAGAGAGCCGAGATTTGCATTCATGACAAAGAGTGAGGTTGATTTTCTGGAAGATGGTTACAGATGGAGAAAATATGGTCAAAAAGCTGTCAAAAACAGCCCATTTCCCAGGAACTATTATCGCTGCACAAGCGCAACTTGCAATGTAAAGAAGAGAGTAGAGCGATGCTTCAGTGACCCAAGCATAGTGGTTACTACCTACGAAGGAAAACATACCCATCTAAGTCCCATGAATACGATCATGCCCCGCCCTAGCTGCTATCCAATTACTCCAGTACCCGCTTCACCTGGTGCCTTCCCTTTGCCGATGCAGTTCAATATTAATCAGTCCTTCAACAACTTGACAAGTTCTTTAGCCATGAATAATCAGCTTGATCATGCTGCTTTTGTTGCTCAAGGAAGGCGCTTTTGCACTTCCGAAATGCTGGGAGACGAGGGGCTTCTTCAGGATCTTATGCCCTCCACGTTGATTAAAGAAAATTACAGATGA

> CaWRKY01-6

ATGGAAAGGGGTGGTGCAGAAAGGGATCATCAGTTAAACAACTACAATTTACAAGTTTCTTTCTCATCATCATCAGTAGCAGCTAATAATATCCATGAATTGGGATTTGTACACTTTGCAGATCATAACTTGAGTTTCTTAGCTCCTTCATCACAATCTTCTCAAATATCTCAGCCACTTCAAGCCGCCAGCGTCAGCGTTACGCCACCTACCACTATCAACACAAACGTTGCAGCTGGCGGCTCGAATAACGTTACTGGTGGTCTAGGGTTTAGCCACAATGAACTTGTCATCAATAGATCTTCTTGGAACAGTGACCAGGTGGAAACACTAGATCCCAAGGCTGTTAATGACGAAAATTGCGGCGGTAATGCCAACGAGGGTAACAATTCATGGTGGAAGACTTCATCTTCAGACAAAGGAAAGGTGAAGATAAGGAGAAAGCTAAGAGAACCAAGGTTTTGTTTCCAAACAAGAAGTGACATTGATGTTCTTGATGATGGATATAAATGGAGAAAATATGGTCAGAAAGTTGTCAAGAACAGTCTTCATCCCAGGAGTTACTATCGGTGTACACATAGTAATTGTAGAGTGAAGAAAAGAGTTGAAAGACTATCAGAAGATTGCCGTATGGTAATAACTACCTATGAAGGTAGACATAACCATTCTCCTTGTGATGATTCTAACTCTTCTGATCATGATTGTTTCACCTCTTTCTAG

> CaWRKY01-7

ATGGCTTTAGATTTGTTTGCGATAGAACAAACTGCTTCAGCTGGTTTAAAATCTATGGACCATTTAATCCAATTTGTTTCATCGAACCCTACAGCTAAACCGGATTGTAGAGAGATAACTGAGTATACTGTTTCGAATTTTCGGAATGTTATCTCTATGCTTAACCGGCCTACCTGCCATGCCCGGGTCAGACGTGTTGGTCCGGTTCAGCCGGTTAAGGTTGCTCCACCGGTGGTTTCTTCGCCTGTAGTTGCTCCACCGATGGTTGCTGCTCCCGTGGAGAAGGAAAAGGAGAAAGAGAAAATGTTTAGGTCAACGCCAGCGTTGACTTTTGACTTTACCAAGCGTAAGGTTGCTGTTCCTGCTGCCCCTTCTCCTGCTGCTGGTGTTGGTGTTGTTTCGAAGGATGTGGCGATGGCAAATTCGACGAATTCGTCGTCGTCGTCGTTTGTGTCGACGATTACTGCTGAGGGAAGTGTATCGAATGGGAGGGTATTCCCGTCAATGGATTTGCCTCCGCGCCCGCCGGTGACGGCGCCGGCTGCTTTCTCCGGGAAGCCTCCGATTGCAGGAAAAAGGTGTCGCGATCATGATGTGTCTGATGAGTTTTCCGGCAGAACTTCTAGCGCCGGCAAGTGCCCCTGCAAAAAGAGCAAACCAAAGGTGAAGAAAGTGATCAGAGTACCGGCGATAAGCTCAAAGACTTCTGATATACCGGCAGATGAATTCACATGGAGGAAGTACGGTCAAAAGCCGATCAAAGGTTCACCATACCCAAGGGGTTACTACAGATGCAGCAGCTTGAAAGGATGCCCGGCTAGAAAGCACGTTGAGAGGGCAACTGATGATCCAAGAATGCTGATTGTAACTTATGAAAATGATCATGAACATCATCATAACATTCAAACTGCATTTTCTGGTGCTGCCATTGGATCCAGAGATGGAAGTTCTGGCCAGAGAATGATGGTGTTTGAGTCAATGGGACAAAAGTGA

> CaWRKY01-8

ATGGATTGTTCTTTCAACTGGCAATACAAGACACTCATCAATGAGCTAACTCGAGGAATTGAACACGCTAAGCAGCTTAAAGCTTACTTGAGCTCTGTAGCTTCCACTTCTGAAAATCAAGAATTGCTTCTGCAGAAGATACTTTCTTCTTACGAGCAATCTCTGGTAATTCTCAAACGGACTGGCTCAACAGTCCACTCCTCGAAGCCTCTGCCGCCAATGTGTGGTGCAATTGAATCTTCGGTATCTGTTGACGGAAGTCCTAAGAGTGATGACAAGAAAAGGTGTTTCAAAGAACATCAGGAGCTCCTTGATATTTCGAAGAAGAGAAAATCACAGCTCACACGGACGGAACAAGTCAAAGTCAGTGCGGAGAGTGGATTTGAAGGTCCTACCGATGATGGATATAGCTGGAGAAAGTATGGACAGAAACACATTCTTGGAGCTAAATATCCTAGAAGCTACTACAGATGCACATATCGTCACATGCAAAATTGTTGGGCAACAAAACAAGTGCAAAGGTCAGATGATGATGCTACTGTATATGAGATCACATATAGGGGTTCCCATAATTGTCGTCAAGCAACAAATCGAGCATCACTAGAGAAGCAAGAACTAAAGAAACAAGCGGTTTATCAAACAGGACAGCAATATTCAAATCAAGCGTTGATGAACTCGAGAGCAAACCTGAAAGTCGATACTGATGACTTGGAAAAGAATGAAACAGCGTGTCCTTTCTCCTTTCCTCCGACATTCTCTGGTTTGACAGATGAAAATCAACATTTCCAGATTTCCGATGTTGATGACAATCGAACTACTTCTCAGTCTCAAGCAGCTGCCAGATGA

> CaWRKY01-9

ATGGATTGTGCAGTTAACTGGGAATATAAGACACTTATAAATGAGTTAACTCAAGGTATTGAACACACAAAACAACTTAGAGCTCATTTCAGCTCTGTTGATTCGACTATCCAAAATCAAGAGCTGCTACTTCAGAAGATACTTTCATCTTATGAGCAATCTTTGTTGATTCTCAAATGTAGCGTTGGTGGCTCGATGGTTCAATCATCGTCGGCTATGATGCCGACGTGTGGTGTCATTGAATCATCAGTGGTGTCTGTCTATGGAAGTCCAAAGAGCGATGACAAGAAACGGAGTTTCCAAGATCATCATGAGGTTATCGATATTTCAAAGAAGAGAAAATTGCAGCCCACGTGGACCGAACAAGTCAAAGTCAGCCCAAAGAGCGGATTTGAAGGTCCTACCGATGACGGATATAGCTGGAGAAAGTACGGCCAGAAGGATATTCTTGGAGCTAAATATCCGAGAAGCTACTACAGATGCACGTATCGTCACATGCAAAATTGTTGGGCAACGAAACAAGTGCAGAGGTCAGACGATGATCCGACTGTATTCGATGTCACATACAGAGGCTCTCATAGCTGTCATCACGCTACTTATTACGTACAACAATCAACATCGCCAGAGAAACGAGAATTCAAGAAAGAGGCCGTTTATCAAAACAGGCAGAATTATTCAACTCAAGCGTTGATGAGTTTGAGAGCAAACCTGAGAGTCGATACGAACGACTTGGACAAGAATGAGCAAGCAGCATGTCATTTCTCCTTTCCTCCAACATTTTCTTCTGGTTTGACAGACGAAAATCATCGACGTTTCCAGATTTCCCATGTCGATGAAAATCTGATAGGTAGCGGCTATTCAGCGTCTTTTGTCTCTCCTACAACTCCTGAATCGAACTACTTCTCAGTGTCGAGCAGCAGCCAGATGAATGGTTACGGAATGATTCATAACTTGAACCATTCGGAATCAGACCTCACCGATATATTCTCAGCCAACACTTCCACAACGAGTTCTCCAATTGTTGGCGATTTTTCACTCGACAATTTGGAGCTAGATACAAACTTTCCATTCAACAATCCAAATTTTTTCTCATGA

> CaWRKY01-10

ATGGATTGTGTTTTTACCTGGGATTACAACTCACTGATCAATGAGCTAACACAAGGAATGGAACACACTAAGCAGCTTAAAACATACTTGAGTTCTGTACCTTCCACTTCTGAATCGACTCCGAATTCGCTTCTTCAGAAGATTCTTTCTTCTTACGAGAAATCTCTGTTGATTCTCAAATGGACTGGCTCAACAGTACAATCCTTACAGCCGCTACCACCAACAGGTGGTGCAATTGAACCTCCTCCGGCGGCTTCTGACGATCGAAGCCATAACTGTGATGACAAGAAAAGAAGCTTCAATGATCATACGGAACTCATAGATAGTTCAAACAAGAGAAAATCACAGCCCAGATGGACTAAACAAGTTAATGTCCGCACAGGGAGAGGATTTGAAGGTCCTCTTGATGACGGATATAGCTGGAGAAAGTACGGGCAGACTAAAATTCTAGGTGCTTATTATCCGAGAAGCTATTACAGATGTAGATATTGGCTCCTGAAAGGTTGTGCGGCGACAAAACAAGTACAACGATCAAATGATGATATTACGATATTTGAGATCACATACAAAAACTCCCATACTTGTCGCGAAGTTACTAATTCTGCATTACAACCAAAATCACCAGAAAAACAAGCCAACCACCAAACGCCAACAGTTGATGGCTATTCACCGTCTTTTGCCTCTCCGACAGCCACTGAATCAAACTACTTATCAGTTTCCGGCAGCCAGATGAATAGCTTTGGAAGAGTTCACAACTCGAACCATTCAGAATCATACCTCAGCGATATTGGAATAATTCAGAACTTGTACCATTCAGACTCAGACCGCACTTATACATTATCAGCCAACACTTCCACAACAAGTTCTTCGATTGAAGGGATGGATGTCCAATGGGCAGAATTTCGGCAGGTCAAAGTGGGCTGA

> CaWRKY02-1

ATGTGCAGCCCGCAAAAAGACATGACGAATAATTATCAAGGTGATTTAGCTGATATATTCAGAGGTGGAAATAGTACCACTTCCGGTGATCAGTCATCAACTAGTGTTGTTCCTGTTCCTGACGGATGGCAGTTTCCTAGCATAAACTATTCGGCCTCTGTGATTGAAGAACCGACGGCGTCGTTGGTTCAAGATTTTGGAGATCCATTTTGTAACTTGAGAGATCCACTACTCTTCCATGATCTTGATATGATTCCACAGGCCAGTTCAAGCTTATTCAACCCGACCCAAGAAAATAATAATCATTTTGGACCAAACATTGATTCTCCATCAATGAAAATTAGACGACCTAACATCTTTTCAACGATGCTTCAGATCTCTCCTACCACCAAGTTGGCCATGTCACCATGTGATATAGCCTGCTCCTCCCCCAACTCTAATGTTAATGTTATTGGTGCATTAGTTCCTACTCAAGATGCTATTATCTCTCCTAATTCCTCCAAAACATGCTTAGTGGAAAACTCTGGCTTGCAGATCTCATCTCCGCGAAATACGGGTATCAAAAGAAGAAAGAGTCAGGCAAAAAAGGTGGTGTGTATACCAGCACCAGCACCTGCAAATAGCCGGCAAGGGGGAGAAGTTGTTCCATCTGATTTATGGGCTTGGAGAAAGTACGGTCAGAAGCCAATCAAAGGCTCCCCTTACCCAAGAGGTTACTATAGGTGCAGTAGTTCCAAGGGATGTTCAGCAAGGAAGCAAGTGGAAAGAAGCAGGACAGACCCAAACATGTTAGTCATCACCTACACTTCAGAACATAACCATCCATGGCCAACTCAAAGAAATGCCCTTGCTGGATCAACCAGGTCTCAGCCTAATAATTCCAAACACACTACAACCTCAAAGAACAACACTAATATTATGCCAAATAATTCTCAATACCAAGGCGATACAAGTTTCAATGAAGATGAACAAAATGAGAGAAACATTAATCATGACAATAATGTTGCACAAGCGAACATTTCAACGTACCCAAAAGTGAAGGAAGAGGTGGCCGAAGAAGATCTTCAACAGCAACTAGGGGAAATGCGTAATGTTGAATTTTCTAAAGGGAGTTATCAGCCAATATTACCAGACTCGTCGAATCAGTGTCATGAGGATTTCTTTGCAGATTTGGTGGAACTTGAAGCTGACCCTCTAAACTTTTTGTTTGCCAATACTCTCTCGGGAGATATCAATGAAGTGGGACAGAAGAAGGCCATCGACGCATTCAACTTGTACAATTGGAGCAAAGACCGTAACACCAACATAAACAACAAGGGTACTCAAGCCGACACATAA

> CaWRKY02-2

ATGGAGATTGAAATTGCAGCTGATGCTGCTATCACGAAGTTTAAGAAGGTAAATTCTCTTCTGGATCGATTCAGAACTGGTCATGCTCGGTTCAGAAGAGCACCTATTGATAATCTCAAAAAAGATTATGTTGACCCTGAGGTTTATTGTCTTACTCCAATTCAACAACTCCCTCCAAGTGCTTACGACCTTAATAATAACCAAATCTTCCAAAATCCAAAACAAGAATTGGTCACAAAATCAATTAATTTTTCGCATGCCCCTGAAATTTGGTGCGCAAATTCGTTTAATATGTCAACGTTAACAGGGGAAACAGAGAGTGAGCAAATTTCAAATCTTTCTCAGGTTTCTTCTGCTGTAAAGCCCCCTTTGTCGTCTTCGTCTTCTTTCAAAATTAGAAAGTGCACTTCTTCGGAGAACGGATTTTCTGGAAAGTGTAGTGGCTCATCCGGTCGATGTCATTGCTCAAAGAGAAGGAAATTAAGACCTAAAAGGGTAGTTAGAGTTCCAGCAATAAGCATGAAGTTGTCAGACATCCCACCTGATGATTATTCATGGAGGAAATATGGACAAAAGCCAATTAAAGGATCTCCACATCCAAGGGCATATTACAAATGTAGTAGTGTGAGAGGTTGTCCAGCACGTAAACATGTAGAAAGAGCTTTGGATGAGCCAACGATGCTGGTTGTCACGTACGAAAGCGAGCATAACCATTCCCTCTCTGTTGCTGAAACAAGTAGTCTCATTTTAGAGTCTTCCTAA

> CaWRKY02-3

ATGAGAAAAGGTGATGATGAAAGTGTCAAAAAGAGAGCTGGTGATGATGAAGTTTCTCAGCCTAATGTCAAGAGAGCTAGAGTTTCAGTTCGGACAAAATGCGACTACCCAACAATAAATGATGGTTGTCAGTGGAGGAAATATGGGCAGAAGATATCAAGAGGTAATCCGTGCCCACGATCATATTATCGTTGCTCAGTCGCGCCATTATGCCCTGTGAGAAAACAAGTCCAACGATGTCTCGAAGACATGTCTATATTGATCACAACTTATGAAGGAACACATAATCACTCACTTCCCATTGAGGCCACAGCTATGGCCTCAACTACTGCTGCTGCAGCTTCCATGCTTCTTTCTGGCTCATCGACAAGTTCTCAATCACCTAAAAATTTCACAAATTTGGCCAATTATTCGAAAACAACACCTCTCTATTTATCCAATTCTTCTTCAAATCCTTTTCCCACCATCACTTTAGACTTCACCGCGTTTCCAACTACTTCATCATTTACTAGTTTCAACTTCCCTTCCAATTTCCAACCCGGCTCGGGACTTCTTTCTAACAGCTTAAGCTTTTCCTCGCCTGAGTCGTCCACAATACCCAAAATCTTGGGTAGTGGATGCCTAAATTATGATTCTACAAGTACATTGCCATATCACAAGAACCTCATAAACATTGGATCATCCCAAAAACAATTTGACCAACCATTTATTGGAAAGAACAACACTAGTACTAGTGATAAATTGAAGGAGGATTCTTCTCAGCAAGCACTAACTGAAACATTGACAAAGGCAATTACATCAGATCCTAGCTTTCAATCAGTGCTAGCTGCTGCTATTTCATCAATGGTTGGTGCTACCAAAACTTGA

> CaWRKY02-4

ATGGGTGATGAACTAAGAGATTTGTACTATCACCAGCCATTTCAAGAAGATTCTTCATCATCAGTTGTTCAAAATATACATATGCTTGATCCATCTTTTATGAGCTATACTAATGAGTATTTATATGGATCAAGTAGTGATTATGTTAACAACAATTCACTTGGGAAACCCTTTGGATTTTCATCATCATCTCCATCACCATTTTCTTCAAGTAAAGATGATATGATAAAACAAGATCTTCATCATGTGGATGCTAATATTAATATTAATAATATTAGTGAAACCCCAGTTACACCAAACTCTTCAGTCTCAAATTCTTCTTCTAATGAAGCTGCTGGTGATCATGACGATTCTAACAAGAAAGATAAGCAAGTCAAGGATGAATCCTTAGAAGATGGTGAAGATGCTTCTAAAAAAGAGAATAAAGGAAAAAAGAAGGGAGAGAAGAAGCAAAGGCCACCAAAATTTGCATTCATGACAAAGAGTGAGGTTGATCATCTTGAAGATGGATATAGATGGAGGAAATATGGACAGAAGGCTGTTAAGAATAGCCCTTATCCAAGGAGCTATTACAGGTGCACAAGTCAAAAATGCCAAGTGAAGAAGCGTGTGGAGAGGTCATATCAAGATCCCTCTGTTGTTATCACAACATATGAAGGTCAACACAACCATCATTTGCCAGCTACACTCCGTGGGAGCGTTGCAAGAATGTTGAATCCTTCCATGTTAGCACAACCATCACCATTAATGGCACCACAAGCTGCAGCTTTTCATCAAGAACTCATCATGGCTCAAATGCCTCAACTTTTTGGCCATGGCAATGCTTTTGGAAGCCCCCCTATGTATCGCCAAAATCTAACTCATCCACTTCAGAATCATCAACAGATGCAGCTTCCTCCTCATCATGACTATGGACTTCTCCAAGACATGGTTCCTTCTATGTTTAATCTTAAACAAGAACCATGA

> CaWRKY02-5

ATGGAAGGAAGATTCAACAATTTTTTTGTTTCTGAGCAAGATGATTCCGAGAATTCACCGGAAAACAGCTCCGACTCGCCGCGTTCCGCCATGTTCAATGATAACAAGATGATCACTTCAACTTCATCCCCTAAAAGAAGCAGAAGATCCATAGAAAAGAGAGTGGTGTCAGTGCCAATTAAAGAAGTTGAAGGATCAAAAATGAAAGGTGAGATAAGCATGCCCCCATCTGATTCTTGGGCATGGAGAAAATATGGACAAAAGCCCATCAAAGGCTCTCCCTATCCCAGGGGATATTATAGATGCAGTAGTTCAAAAGGATGTCCAGCAAGAAAACAAGTAGAAAGGAGCCGTGCGGACCCAAACATGTTGATAGTGACATATTCTTGTGAACATAACCATCCTTGGCCGGCTTCTAGAAGTAATCAACACAACCATCGTACAATTACCCCTACATCATGCACCAATAATAACACAAAGACAAAGACGAAAACAATAGCATCACTAACCGCATCACCAGCAACAACAACAATAATAACGACTTCTGATATTCCGATTTTACACTTTGAGCAACAAAAGGCCACGACCGATTTCGCAGTACGCCCATCGGAGCCCAATTCGGACGAAAAATTTGTCAATCTTGGTGAATCATCACTTATTAATGCTGAATTTGGATGGTTTTCTGATTTAGTTGAGTGTAACTCTACTACCATACTAGAAAGTCCAATTTTGACCCAAGTCGAAGTTAATGATTTTGACATGTCATCAACATTGACAATGCAAGAGGAAGATGTGTCACTTTTCGCCGATCTCGGAGAGTTACCGGAATGTTCAAGGGTATTTGGCCGTGGAATGATGGAGAGGGACGAGGAGCGCGACCGACATAGCTTAACACCGTGGTGTGGGACCACAGGCTAA

> CaWRKY02-6

ATGGCTGTGGACTTAATGATGGATTATAGAAACACTAGCAATAGTAGCAGCAATAATTGTATCAACTTCGTAACCAAATTGGAAGAAAAAGCAGTTGTTCAAGAAGCCGCTTCTGGTCTAGAGAGTGTTGAGAAACTCATCAGAATGTTGTCTCGTAATAAATCTCCACAAATTCAGCAGCAAAATAAATCCCCCATGGAGATCGAACTAGTGGCTGATGCAGCAGTAACAAAGTTCAAGAAGGTAATTTCACTTCTAGATCGAAACAGAACTGGTCATGCTAGATTCAGAAGAGCCCCTTTAGCTGCTACCACTTCCCCTTCACCAACAAATTGTAACAAAGATATCGTCGACACTAAAGTTTATTCTCCAACTCCAATCCAACAAGTCCCTTTAGTATCCTATGAACATTATAACCCTCTCGTTCCACCAAAGACGATTAGTTTCTCATATTCACCTGAGATGTCTCGTACAAACTCGTTCAATATATCATCGTTGACAGGGGATACGGAGAGTAAACAACATTCTTCATCTTCAGCAGCTTTCCAGATTACCAATCTTTCCTCTCAAGTCACTAATTCTGCTGGAAAGCCTCCGTTGTCTTCTTCTTCACTGAAAAGAAAGTGTAGTTTATCAGAAAATGCTGTATCTGGCAAGTGCAGTGGACCCTCTGGTAGATGCCATTGTTCCAAGAGAAGGAAGTTAAGGCTGAAGAGGGTAATTAGAGTACCGGCAATAAGCATGAAACTGGCAGATATTCCACCTGATGATTACTCATGGAGAAAATATGGACAGAAGCCAATCAAAGGATCTCCACATCCAAGGGGATACTACAAGTGTAGCAGTGTAAGAGGGTGTCCAGCACGCAAACATGTTGAAAGAGCATCGGATGATCCAACTATGCTCATTGTTACATATGAAGGAGAACATAATCATTCACTTTCTGTTGCAGAAACAAGTAGTCTTATTTTAGAATCTTCTTAA

> CaWRKY02-7

ATGGACAAAGGATGGGGTCTTACCCTTGAAAGTTCTTCTTCTTCTGATAAAGTTGGTTTCTTCATGAACAAGCCTGTTTTTGGTTTTAATTTAAGTCCAAGATTGAACCCTGCTGAAATGTTTCCCAGCTCCGATGATAAACGTGCCATCGTTAATGAAGTTGACTTCTTCTCTGAGAAAAAGCCTATTGTAAAGAAGGAGAATTCTCAGGGTGACAGGACTGATCAATGTGTTGTAAATACTGGATTACAACTTGTGATTGCAAACGCTGGAAGTGATCAATCAACAGTAGATGATGGGATTTCATCGGAATTAGTACTTGAAGATAAACGAGCTAAAATTCAGTTGGCGCAATTGCAAGTTGAGCTTCAGAGGATGAATTCCGAAAATCAGCGTTTAAAAGGGATGCTTACTCAAGTTAATAACAGTTATTCTGCACTTCAGATGCATCTTGTTACACTCATGCAACAGCAGCAGCAACAACAACAGCAGCAACAAATGATTTCAAGAACTGAAAGTACACATGCCCATGAGGTTGTCGAAGCCAAGTTTAATGACGAAAAGAAGCAAGAGAAAGAAGGGACCATAGTTCCAAGACAATTCATGGAGCTGGGCCCAAGTGGTTCCAAAGCTGATCCACTGGATGAGCCATCTAATTCCCATACTTCATCAGAAGAAAGAACGCTTTCTGGATCACCTCGCAACAATATGGAATTATTGTCAAGGGATAAGGCAATTGGCCGCGAAGAGAGTCCAGAATCTGAAAGTTGGGCTCCAAATAAGGTCCCTAAATTAATGAATTCATCAAAACCTGTTGAGCAACCAACCGAAGCAACTATGAGGAAAGCTCGTGTCTCCGTCCGTGCCCGATCAGAAGCTCCTATGATTAGTGATGGCTGCCAGTGGAGAAAATATGGTCAAAAGATGGCTAAAGGCAATCCGTGTCCACGTGCTTATTATCGTTGCACGATGGCTGTTGGTTGTCCAGTGCGCAAACAGGTGCAAAGGTGTGCGGAGGACAGGACAATCTTAATAACAACATACGAAGGTACACATAACCACCCCCTACCACCAGCAGCCATGGCAATGGCATCCACTACATCAGCAGCAGCAAACATGTTGCTCTCTGGTTCCATGCCAAGTGCTGATGGACTTATGAACACAAATTTCCTAGCCAGGGCCATGCTTCCATGTTCATCAAACATGGCAACTATTTCAGCATCTGCTCCATTTCCAACGGTTACATTGGACCTCACAGCCCAAAATTCAAATGCTGCACTGCCTAATTATCACCAAAGAGTTAACCACGCTAATAATGCTCAATTCCAATTCCCTTTACCAGCTGGACTTAATCACCCGAATTTCATCGCTTCAATGTCAGCTCCACAAATGCCTCAGGTTTTAGGCCAGGCTATGTATAACCAGTCAAAATTTTCGGGTTTACAAGTTTCTCAGGACAATATTCACCACCCATCAATTTCTCATGACACACTCTCCGCTGCCACGGCTGCGATCACTGCTGACCCTAACTTCACTGCCGCCCTTGCCGCGGCCATCTCCTCCATCATAGGTTGTGGTTCTCATCCCAACAACAACGGCAACAGTACCATGTCTGGTCCATCAAGCAATAACAACAACACTAGCAGTTTCCCAGGGAACTAA

> CaWRKY02-8

ATGAATTTGGCGTTGGCTCAATCTCCTTTGTTCATGATTCCCTCTGGTTTCAGTCCTTCTGGGTTCCTTAATTCTCCTGGATTTCTTTCCCCACTCCAGAGTCCCTTTGGAATGTCCCATCAGCAGGCTCTAGCACATGTTACTGCACAGGCTGAATGTTCTAGTTCATACATGCAAATGCAAGCCGAAGATCAGTGTTCCGCTCAGGTGGCTTCAGCAGAAGCAGCATTAGGAAATGAGTTGTTGACTGATCCAAAGGAATCTTCTTTGCAGATAAAGGAATGTTTGCAGCCTAGATTGGATAAGAAACCATCAGACAAGCAGGGTAAGCAATTTGAACTGACGGAGGTTCCTCAATTTGAGAACAAGACATCCTTTGGTGCTTTCGACAAGTCAGCTTGTGATGGTTATAACTGGAGAAAATATGGCCAGAAAAAGGTTAAGGCTACTGAATGCCCTCGGAGCTACTATAAGTGCACGCATCTCAAATGTCCAGCGAAGAAGAAGGTTGAGAAATCCGTTGATGGTCACATAACTGAGATCACATACAATGGCCGGCACAACCATGCTCAACCAACCAAACAAAGAAAAGATGGTTCTGCTTTGGATAGTACAGACGGCTCCGGAGTTCAACCAGACATTAGTACACATGATTGGACAGTAATGAATAGCTCGGATGGGTCTTCTCCTTCTCATTCTGAACAGGTTCCAAACCAAATGGCATCTGAACTTGTGAAAAAAGAATGTGATGAAACCAAAAGCAATTTGATAGAAGTAGATGAGGGGCATGATGAACCAGATGCAAAGAGAACGAAGATGGCAGTTGAGACTCTAGCTTCATCACATGGCACAGTAGCTGAATCCAAGATTATTCTGCAGACAAGAAGTGAAGTTGATATCTTAGATGATGGGTACAGATGGCGAAAATATGGGCAGAAGGCGGTAAAGGGGACTCAACATCCAAGGAGTTACTATCGTTGCACATATGCTGGATGCAATGTCCGCAAACAAGTCGAGAGGGCTTCAACTGATCCAAAAGCTGTCATAACAACATATGAAGGCAAGCATAATCATGATATTCCTACTGTTATTAGGAATAGAGGAACGAGGAATACAGCCAAAGATACTTGGCGTTGA

> CaWRKY02-9

ATGGAGAATAACCAGCCCATGCTCCTTCTTGGTTCAGCAAGTAGTTATTACAACTCTATGAATGGAGGATTAAAGACATCGTTCACCCAGATTAGTCGTGATCAGATGGAAGTGGATACATCAGAAAATCATAATAAATATATATCATCATTATCGGTTAAGAAGAAGGGAGATAATAAGAAAATTAAGAAGCCCAGATTTGCTTTCCAAACAAGAAGTCAGGTTGATATACTTGATGATGGCTATCGTTGGAGAAAATATGGACAAAAAGCTGTCAAGAACAACAATTACCCAAGAAGCTACTACAGATGTACACATGAAGGATGCAATGTGAAGAAGCAAGTACAACGCCTTTCCAAAGATGAAGGAGTTGTAGTGACCACTTATGAAGGCATGCACACCCATCCTATTGACAAGCCCAATGATAATTTTGAACAAATCCTCCATCAGATGCAGATTTTCCCTAATCATCCCCTTAATTAA

> CaWRKY03-1

ATGGAAGATTCTCACTCTCACTCTCACTACCCTCGCCCCTATAGCAACTCAGCTCCACTCTCTTCCATCAATGAAACTTCTGAGCAAGTCAAATTTTCAAGCTCCGATGCTGCGCTCTTCTCCAGCTCTGATGCTGCGTTCGTCTACTCCTCCGCCTTTGGATCGAACAGTAGTAGCAGTGCTAAGTACAAACTGATGTCACCTGCTAAGCTCCCGATCTCTCGCTCCCCGTGTATAACTATCCCTCCTGGTCTCAGTCCTTCTTCCTTTCTCGAATCTCCCGTTCTTCTTTCTAACATCAAAGCTGAGCCTTCTCCGACCACAGGTTCCTTCTCCAAGTTTCAACTAATGCAAGGCTCTAGTGGGAGTGCTGCTTTTTCATTGATGAGAAGCTGTTCCAGTGGAAATGCATATGGTGAAACGACCGGCGAATTTGAGTTTGAATTTCCTATTGGATCTAGCTCTACATCAGGATCACTGGCAAAGGAAGCTGTGATTTGTGCAGGTTTCAACCAACAGCAAAGTGAACCACTGATTCAAGTTCAAAATCGATGTCCTTCTCAATCATTAGCACCTCCAGCTCTTGTTAAAAGTGAGATGCCTAATTCAAAAGAGCTGAGTCTACCTACACCTGTTTGTTTAGATGCCTCGTTAATTAGTACTGCTGCTGCTGCAACTGATAATGAGGAGGTAAATCAAAGAGGTCAATCAAATCCAAGCTCGCACAGGTCATCTGCTGATAATAAAAATGTATCATCAGTAACAGCTGATAGATCGTCTGAAGATGGGTATAACTGGAGAAAGTATGGCCAGAAACTTGTCAAAGGAAGTGAATTCCCAAGGAGCTACTACAAATGTACATACCCAAACTGTGAAGTTAAAAAGATATTTGAGCGCCCTCCTGATGGACAAATAACAGAGATTGTGTATAAAGGTTCCCATGATCATCCTAAACCCCAACCCAACCATAGGTTTACTCCTGGTGCTCTTACGTCCGTCCAAGAAGACAGAGGTGAGAGAGAAGCATGCCTCACTGGTCAAGAAGTTATTCCTCTCTCTGGTTTATCTTTTCTTGAAGACAAATTCAACACTAATGCCCAGACTAGTAACACTGAGCCCAGTGGTACTCCACTATCACCTCAACAAGCAGATGATGATGGTCTTGAAGGTACAGTGTCTCAGTTGCACAGCTCTAATGATCAGATGGACGAAGATGATTCATTTGCAAAAAGAAGCAGGAAAATGGATGGTGTCATGGATATTATACCAGTTGTTAAGCCTATCCGTGAACCACGTGTTGTTGTTCAAACTGTGAGTGAAGTTGATATATTGGATGATGGATACAGGTGGCGGAAGTACGGTCAAAAGGTGGTACGTGGTAATCCTAATCCCAGGAGTTATTACAAGTGCACCAATGCTAGATGCCCTGTCAGGAAACATGTGGAGAGGGCATCCCATGATCCCAAAGCTGTAATTACTACATATGAGGGGAAACACAATCATGATGTACCAACAGCCAGGACTAATAGCCATGAAATGGCAGGATCAGCGCCTGTAACTGGGAGTTCTAGGGTCAGAGTGGAAGAGAATGGTGCAATTAGCTTGGATCTAGGTGTTGGTATAGGACATGGCATGGAGAATAGACGCAATGGACAACTTCACACACTGCCTGCAGAAACTGTACGTAGCCAAGGTCAGGTTTCAAGTTCTATTGTGATGGTAGTACAGCCAGCTGCAGTTGCGGCATGCTACAGCATTGTAAATGGTGGCATGAACCGTTTTGGAACTATAGAAAATCGTGTTCAGGGCACTGGCTTTGAGACTCTGCCCTTGCAATCTTCTGCTCAATATCCTCAAAACTATGGAATGATACTTTTGGGCCCATGA

> CaWRKY03-2

ATGGAGGAGCTTGTTGATGAAACCCCCCGTAAAAGATTGATCAAAGAACTTGTTGAAGGAAAAAGCTTTGCAAAGCAACTTCAAAGTCTACTTCAACAACCTAATATTGAACATTATGATGGATCAGTCTTAGCTGATGAACTTGTTCTCAAAATCTGGAGATCTTTTACTCAGGCTATTACTGAGTTAAATACGTTGGTTGATTCCAACAGTATTTTAGTCCAGACCCAGATGGAGGTGGAAAAGACCGAAGAGGTGGATCAAGCCGATACCGGCGACCGGTCTAATTCTGAATTGAAGAAGAAGGGCAAACAAGGGGGAAAAGACCGGAGAGGTTGCTACAAGAGAAGAAACAACTCAGGTTCATGGATGAGAGAATCCGAAACAATGAATGATGGTTGTGCATGGAGGAAATACGGGCAGAAGAGTATACTCAACTCAAAATATCCTAGGTGCTACTACAGGTGCACCCACAAGTACGATCAAGATTGCCGGGCCACAAAACAAGTTCATATAATGCAAGAAAATCCAAAACTAATGTACCACACCACATACTTTGGCAACCACACTTGCAATCCAGCAAAGATTCGGAAGCATATAAATAATGCACAATTCAATCATTCTATGCTGGAATGTCCACCTTTTGAAGTAAAACCAAAAATCCCTAGCAGTGTCACTCATGATTCAACAGAAGAAGAAGAAGAATCATTAAAGGGACAAAGTGATAATGTATCATCAACTATGGATTCTTACTTATGGGAAGATTTTATGCCTTCTTCTCCCTCAGCTCATGATTCCACCTTGGCTTCTCATAATTCTTCTTATTTTCAAGGACTGATTAGTAGTGAGATGGGAGATCTTGTTAAATTTAGTGACTTTGAGGCCATAGAGTTTTTTTGA

> CaWRKY03-3

ATGGATGACAACAATTGGGATTTAGGCGCAGTTATAAGAAATTGTGGAATTAATAGACCTAGTAATGATATAACTCCTAACTTAGGCTCCGAATCATTAAATTTTGATGATGATGATTTGAATTTTCTTGACAGAATTTTTGGTGTTGACAACAATAATTTTGATTACATAGCTCCTACAAATTTTTCAATTTCTCGTCAGGAAAAATCATATGATCAATTTGATGACATCATAAACCCCACAACTCCTGTTTCTATAATTGCTAATCCATTTAAAATCACTAACCAAAATGATAACCAACAAATATACTTACCACCCATTCAACCAGCTCAAGTTAGCCAACAAGTTTTTCTTTCTTCACCATCAGGTGTTGAAGCACGAGAGTGTGTGCCAACAACAACAACTACTACAATATCACATGAATGTATAAATCTGCAACAACAACTCTTGATGTGGGATTCCACCTTGACCATGAGGAATCCTCCGATTCAAATTCGTAAAAGTAAAAATCAGTCGATATGGACTACATATGAATTGTTTCAAGAGGAACTCACAGATGATATATGGTCATGGCGCAAGTATGGTCAGAAGTTTATCAAAGGTTCTCCATTTCCAAGGAACTACTTTAAGTGTAATACATCAGAGCTCTGTCAAGCAAGGAAACAAATTGAAAAAAGTTCAAAGAATGATTGTTTTTTCTTGGTAGCCTATTCCGGTATGCATAACCATGATCCACCCATAATTCGTAGATCTTTTTCTGATTGGAACCATAGTTCCAAATATAAGCTTCCAAAAGGCATAAATATTATTCCCAAAGCATTAAAATTGAATGCATCACCTTTCTCATCAAAGAGTGGTAAACGTTATAGAGCTTCATCTACACTTGAAACTGAGAGCACATCTCGCAAAAAAAATAAAATGATTGTTGAAACCATGAAGAACAATGTTGATGATAAGGAGGAGGAGAATATTAATGAAGATGTTCCCAAGGGATTTGAAGAACTCAACTAA

> CaWRKY03-4

ATGGTTCAGCAACTAACTTTTAAATCTTCTTTACACCGTTTTTGTCTGGTATTCCACGTAAACACACACTTCCCAATCGAGTTAATTTCTGTAATGGACAATTACGGAGCTGACAATACTAATGTTGAGTTTAATAGGATCATAAATGAATTAACACAAGGTCGGGACCTTGTTCAACAACTCCAGCTCCATCTCAATGCTCCTAACTATAATTCCTCTGCTTCATTCGAAAACACCCGGGAAATCTTGCTTCACAATATCCAGTCCAAATTTGACAAGGCTCTGTCCATACTCCAATACAATAGTACTACCGGAGACAATTCTAATTCACCACTAACACATTCTACTTCCCCTGCCATTCCTGTTTTTGGAGTGTCTGATTCTCCACGATCGAGCCCTCCTCATAGTGAAGATTCTGATCGTGACCTCGAATCCAAAGATCCTCATGCTACCCGCAAGAGAAAGAGCACCACTCCACGGTGGACAAAGCAAGTTCAAATTCATCCAGGAGCACCACTTGAAGGGACTCTTGATGATGGTTTTAGTTGGAGAAAATATGGGCAGAAAGATATTCTTGGTGCCAAACATCCAAGAGGTTACTACAGATGCACGCTTCGACATGTCCAAGGTTGTTTGGCCACGAAACAAGTTCAAAGATCCGATGAAGATCCCACCATTTTCGAAGTCACTTATCGTGGAAGGCACACTTGCAGTCAAGGTGGTGGTGGTGGTGCTAGTGCCAGTAATGTGCATCCAGCACCACTACCTTTGGTTGTAACAATACCTCAAAATCAAGAACCAAACTTAGGAAATCATGAACAATATCAACTTATCCCCGCACCACATCAAAATTCACCTGAAATTCTCTTGGATTTTCAGAAAAACCTCAGTATCTCCAAAGATGACTTCAACTTCAATACTCACCATGATCATCCTAACAATGTGCCATATATTCCCCCGTATAGTAATTTCCCTTCATCATCGTCGTCCCACGTTAACACTGATCATCAAGATTACACCTTTGTGGCTAATTTCTCTACAATTCCAATCAACAACTTTGTGGAGAATTTTCCTCCTTCCTCTAATAATATGTCTGCAGGAACTTCTCAAATGAACAATGCAGACTACCAATTTAACTCAATGGGTTTTGAATCAAACTTCCCGTATAATTATCAAGGATTCTCTTCCTAA

> CaWRKY03-5

ATGGCTGAAAACCAAAACGACTGGGATTTATGGGCAATTGTGAGAAGTTGCTGCAACATGAACAACTCTGTTCATGATGATGTGATCAGTTTCGACAACGTTAATAGTACCTCTGTTCTTGTCGATCATGGTGTTCATGAGGATCCTACTCATGGTAACTCCGCTAATAATGCGAGAACATCAACTTCTTTTCAAGAACAAAGTGGTTGCGCTGGTGATTTCAGTGACTCGTTCGCCACAGAAAACAAGCACTATTTTGGGTTAGATGAAGTTCTCGGACTTTCCAAGAACGTTAATACCAACTCAAGAATTGAACCCCACAACCCAGAGAACCAAACTGAATCTATTACAGACACCCCACTTGTAGAACACGAGAAGAAGAAGAACAAGAACAAGAAGACAAGATATTCATTGTCGAGTTCAAGTATTGAGGCAGGCAAGGCGTTTCCTTACTGCAGGAAGAGCACTGAAAGATATGAAGTATTGGCTGAGAAATTGAGTGAGGCGGATCAATGGAGATGGAGAAAGTATGGGATGAAGCGAACTGGCGGTTCACCTTTTCTCAAGAGCTATTATAGGTGTAATCAGGGTGAAGATTGTCCGGCAAGGAGACATGTGCAGCAAAGCTCAACAGATTCAAACAAGGTGATTGTAACTTATAGAGGCCAACACAGTCACCCTCCTCCTAACCAACACATTGCAACAGTACAGGGGAACCATAATGCTGCTGCACCAGTAGAAGACCCGCCATTTCCCTCTTCTCCCTCTACTTTGCTGTTCAACTAA

> CaWRKY03-6

ATGGAGGCTTCTTTCAAAAAATCAAATCTTCATGGACATGTATTCAAAGTGGAGAAGATCAACGCTGATGACAAAGGTTTTGTTGAAGATACTAAAGTTCTTAAGTTTAGCAAGAAAAGAGAACTCCATGAGGACCATAAGTCGAAGTCATCTCAGCTTCAAAAGGATTATCTCACCAGTGACAAGGAGGATGATCAGCTCGAATCAGCCAAAGCCGATATGGAAGAGGTAATGGAAGAAAATCAAAGGCTGAAGAAGCATTTAGATCGAGTTATGAAGGATTATCAGAACCTTCAAATGCAATTCCAAGAAATTTCTCAAAGAGGTGTCGAAAAATCCAACAACGTTAAACATGATGAAGCTGAACTTGTGTCCCTTAGCCTAGGAAGAACTTCAAGCGACACAAAAATAGAGTTATCCAAAATCTTGAACAAAAAAGAGAATGTAGAGGAAGAAGATAACCTAACCCTAGGATTGGATTGCAAGTTTCAATCGTCTGCGAATGCGCCTACAAAATCTTCACCTTCAAATCTCAGCCCGGAGAATAGCTTAGGTGAAGTTAAGGATGAAAAAGGAACCGAAACATGGCCAGCTCACAAAGGTCTCAAGACGATCAGGGATGAAGAAGATGATGTGGTGCAACAAAACCCTACTAAAAGAGCTAAGGTTTCTGTCAGAATTAGATGTGATACCCCAACGATGAACGATGGATGCCAATGGAGAAAATACGGACAAAAAATTGCAAAGGGGAACCCATGTCCTAGAGCTTACTATCGTTGCACAGTAGCACCATCTTGCCCAGTTAGAAAACAGGTTCAAAGATGCATTCAGGACATGTCAATCTTGATCATCACATATGAAGGAACACATAACCATCCACTTCCTCTTTCAGCCACATCAATGGCTTTCACCACTTCAGCTGCCGCTTCCATGCTATTGTCCGGTTCATCCACCTCCGAATCAGGCTCTACTAGTACCTCAACCTCTGCCATTTCCAACGCGCTCAACTACTATCTCTCCAATACCTCAAAACCAAACCCATTTTACCTTCCAAATTCATCTATTTCATCTTCATTGCATTCTCAACACCCTACAATCACTCTTGATTTAACATCAAACTCGTCCACTTCCTCATTTCCCAACAATCACAGAATGCCGCGATATAATAATAATTCTTCCACAAATATTCTCAACTTCAGTTCCTTTGAATCTAATCCTGTTCTTCCTATGTCCTGGAGCAATGCAAATAACCAAGCCTATAATAACAAGAACCAAGAAATTCCTTCCCAATCTTACCTACAAAACACTATTAGCGCGGCGCCTACACAAACTTTATTACAACAAGACACAATTTCAGCTGCAACAAAAGCCATAACATCCGACCCAAAGTTTCAATCTGCATTGGCTGTTGCTCTCACATCTATCATTGGCTCGCGTGGAGCAAATCGTCATATTGATGAGAAATCTGGCAAGGATTTAAAGGTTAACGAACCATTTCCAGTGCTTTGCAGCTTCCCATCAACCGCTACAAGTCCAATTAAATGTTCTCCAAACAATACACAGCCTGAAAAGTCATTGTTTATGAGGGAATCGACCAGTTCAGTGCAATTTACAGCCTCCAAGACTAAATGTCACAAAGATTACACACTCTGA

> CaWRKY03-7

ATGCCTGACAAAGATGATCAGGCAGGTGATTTGTTGGAGGAACTAAATAGAGTAAGTGCTGAAAACAAGAAACTGACTGAGATGTTAACAGTTATGTGCCAGAATTACAATGCATTGAGAAACCAATTGACTGAATATTTGAACAAGCAAAACAGTACTACTAGTACTGCAGCTGATAATAATCATGATCATCATAGCGATGGATCGAAAAAAAGAAAAGTCGAAAACAACAACAATGAAATTGTGAAATCAGTTCAAGGATTACACTCAGAGAGCAGCTCAAGTGATGAAGATTCATCTAACAAGAAACCAAGAGAACAACACATTAAAACTAACACTTGTAGAGTTTATGTCAAAACCGAAGCATCTGATACTTCTCTTATTGTGAAGGATGGATATCAGTGGAGGAAATATGGTCAGAAAGTAACAAGAGATAACCCATCTCCCAGAGCTTATTTCAAATGCTCTTTTGCTCCTACCTGCCCAGTCAAAAAAAAGGTGCAAAGAAGTGTGGAAGACCAATCGATTCTAGTAGCGACATATGAAGGAGAACACAACCATTCCAAAATGGATGGTTCAGGCCCCGTTACAACTTCCCCGTCTAGCCGATTAAACCCGAAAAATACTCTTGTGGGTGCTAATACTACTACTGTCATGCCATGCTCCAGTACTAGTATCATCAACACACCATCAGGACCAACCTTAACATTGGATCTTACACAACCAAAAAAATTACAAAATGACCAAAAGAAAGTGAACAGCAATACTAGTACTAGTAATGCAAGTGGTCAAAAAAGCAAATCACCAGGAGGACATGATCATCATCAGCAAAATAGACCAGAGTTTCAACAGTTGTTTATAGATCAAATGGCTTCTTCATTGACTAAAGATCCAAGTTTTCAAGCAGCCTTAGCCGCTGCCATATCGGGAAAATTCTTACAAAATAATCATACAGACAAATAA

> CaWRKY04-1

ATGTCCACTGGTTATAGACCCAATAATTTCTCATCAAAAATGGAAGAAAATTCCGTGCAAGAAGCTGCTGCTGCCGGCCTTCAAAGCGTTGAGAAATTAATCCGGTTGCTTTCTCAATCCCAACAACAACAACAACAAAATCATCAGCAACAAACAAATTTTCAGAATTCATCGTCGAATGATTATCAGGTTGTTGCTGATGTTGCTGTTAACAAATTCAAAAAGTTCATTTCGTTACTTGACAAAAATCGTACTGGACATGCCAGATTTCGTCGTGCTAAACCCCAGCAGAACAAGCAACAAATGGAGGAATCTGAAAAGCAACAATCTAGTGCTACAAAAATTTACTGTCCAACTCCCATACAAAGATTACCACCGTTACCACACAACCACCATCAACAAATACTAATCAAAAATGGATCGATCGAGAGAAAAGAAGCAGCGTCATCTACAACTATTAATTTTGCTTCTCCGTCACCGGCAACTTCCTTCATGTCGTCGTTAACAGGAGAAACGGAGAGTTTACAGAACTCTTTGTCTTCCGGATTTCAAATTACGAATCTTTCTCAGAGAAAGTGTAGTTCTATGGACGATATTGCCCTCAAGTGTAACAGCGCCGGTGGATCCTCCGGCCGTTGCCACTGCCCTAAGAAAAGGAAATCAAGAGTCAAAAGGGTAGTGAGAGTTCCTGCAATTAGCATGAAAATGGCTGATATTCCACCTGATGATTATTCATGGAGAAAGTATGGTCAAAAACCCATCAAGGGTTCTCCTCATCCCAGGGGATATTACAAATGTAGCAGTGTACGAGGATGTCCTGCACGTAAACATGTAGAAAGAGCATTAGATGATCCAGCTATGTTGATTGTTACATATGAAGGAGAACACAATCATTCCCATTCCATTACAGAAACACCAGCAACTCATGTTCTTGAATCATCTTAA

> CaWRKY04-2

ATGGGGGAAAAACTGAAAGTTCCAGCAGTTTCAGCACTTCCTGCACTAACAATTCCACCTAGGGAAACATTTTTTGGTGGTGGAAATATGTCTTATTTTAGCCCAGGTCCAATTACCCTTGTGTCCAGCTACTTCTCTGAATCTGAGCACCCTTCTTTCTCTCAGCTCCTTGCTGGGGCCATGGCTTCTCCACTAGCAAAACCCCTTTTGACTAAAGAAGAAGAAGCTAATTGTAAAGAGGGGAATTTAGGGTATAAGCAGAATCGACCGATGAGTTTAATGGTGGCTCACTCTCCTTTCTTTACCCCTTTTAGTCCTTCTGGATTGCTTAATTCACCTGCCTTTCTTTCCCCACTTCAGAGTCCGTTTGGGATGTCACACCAGCAAGCCCTAGCACATGTTACAGCGCAGGCAGCATTATCCCAGTCTTACCTACAGGGGACATCAGCACAAGTATTAGGGACATCTGACCTAGACGAATCTTCTTTACAACCTCAATTAGATACTATGCCGTCAGACCAGCAGATTAAGAAGTTTGAACTGCCACAGATTTCTCAATCTGAGGAAAAGCCATACCTTAATTCAGTCGATAAGCCAGCTTCTGATGGTTACAATTGGAGGAAGTATGGCCAGAAGATGGTTAAGGCAAGTGAATGCCCTCGGAGCTACTATAAATGCACACATGTCAAATGTCCTGTCAGGAAGAAGGTTGAGCGATCCGTTGATGGTCACGTAACTGAGATAACATATAAAGGCCATCACAATCACGAGCTTCCTCAACCAAACAAACGCAGAAGAGATAGTGGTGCTCAGGATGGTTCAGACTGCTCCAAAGCTAACCCTGAAATTGAAACACATACTGAGATAGAAACCAGCGGCTTAAATGGGGCGCATCTTGCTCATTCTGAGCAGGTGTCTACTGAAAGGGCATCTGAACCTCCAGTTTTGAAGGATTATGATGAAATTGTGGACACTGCAACAGCAACAGGCAAGGAACAGGATGATGAATCAAATGTGAAGAGAATGAAGACAACAGTCGAGACTCCTATTCTTTTTTCATCACATAAAGCGGAATCAGAATCCAAGATTGTTGTGCAGACAAGGAGTGAAGTAGACATTTTGGATGACGGGTTCAAGTGGCGTAAGTATGGGCAAAAAGTGGTGAAGGGGAATCATCATCCGAGGAGTTATTACCGTTGCACATATCCTGGATGCAATGTTCGCAAACATGTTGAGAGGGCTTCAACAGACCCAAAAGCTGTCATAACAACATATGAAGGCAAACACAATCATGAAAGTCCCATTGCTAGAAACAGAAGCCATAGCGCAGCCCAAGATAGTACTTGTCAGTTGAACGAGCAAGAGATTGCAACTTGGAGGCCTTCACTTCATGAAAAAGTTGCTCTTCATGCCAATGAAATACCAGTGTGTAGGCAGCTGAAAGATGAACATATGGCAGCATAA

> CaWRKY04-3

ATGTTTGGCTCTTCAACTTTCCAAGAAACAAGCAATGTCACTTCTCATCATTACCAAACTATAAACCCTAATTTTGCTTTTCATGATCCACTCATCAACATGAATCAAGATCATGGTCATAACAATAATAAGTATCAAGATTTTGATACTTCATTTCTTGATATGTTGCTTGATGGTGGTGATCAAGAATATTATTCCAATTACTTAAATAATAGCTACAGTAATAATGTTTCATTTTATTCAGAAAATCCTTTCACGCAGCAAGAGATAAGCAGTAGTACCTATAGTACTAGTGGAAACTCATCAACAGCTAGTTCATTTGATGCCACACTAACAAATATTCACATGAATCATGAAAACTCAAGCATGGGGATAGAAAAAGAAAAGAAGGGTGAAAAGCATGCAATTGCTTTTAGAACAAAGACAGAGCTTGAGATCTTGGATGATGGATACAAATGGAGGAAATATGGGAAAAAGAAGGTCAAAAGCAACACAAATCTAAGGAATTACTACAAGTGTTCAAGTGGAGATTGCAAAGTAAAGAAGAGAGTAGAAAGAGATGGAAATGATTCAAGCTATTTGATAACTACGTATGAAGGACGACACAACCATGAAAGCCCCTTTGTCATTTATTGCCATGATGAAATGCCAACATCCAAGTATTTGAGCTGA

> CaWRKY05-1

ATGATTGAACCTTGGTCAAGAAGATGCTGGCACGAAAGACAAGAGTACATACAAGCTCGATCTGGAGAAGAGGTTCCACCTGAAGATGGATACAGTTGGAGGAAATATGGACAAAAACTTATCGTAGGTGCAAAATATCCCAGAGAACATTATCGATGTGATTGTCGTCGTTTATCTTATCGTGAGGCTACTAAGATGGTCCAACGAAGTGAGGCAGAGCCATTATCTTTTGAAGTTACCTATGGAGGAAGTAACAGTTGTGGTCAAGAAAACAAAAACCAAAACGGAGAACATGTTGTGCTAACAAAAGAAACACAGCGTGATGAGGTTGGAAGAGCAGCCGGGGAAACACCTGAATGCTATACTCCGGAAATGGTTTCAACACCAAATACTTCATTCAACAATTCGTCCGCAGGGGTTGTGTTCTCAAACTCCAATCCGGTATTTAACATCACCAATTCCGATTTAATCCCCACACCGACTTCTTCACCTTATCCAGACACGGATATTTCACTTGAGGATGATAGTCTCACTGTCTTGTTTGATGATGTGCCTGAAAATGCTAGAAGCACCTATAAGTGCAATGCAAACTAG

> CaWRKY05-2

ATGGAGAAGGAAAGCAATCTACAATCAGAGTACCCAATTGAAGACGGAAATGGAAATTTGAGTACATTTGTCATGAACAATCCAAACATGATGAGCAACAACTTTGATATTGAAAAAGATTATTCATTAACCTTTTTATTAGAAAATATGTTTGGTGGTACTCATGTTGATCATCAAGATTATGATTTTATTACAACAACAACTAGTAATTCCATTTTCGATTTGCTAATGTTGCCACCTCATCAGCCTATTATTACTACTTCTACAGTTCAAGATTCAACAATATCTGATCAGTTGATTAATGCTCCAGTGACTCCAAATGTATCATCGATTTCTTCAACTTCAACTGAATTACCTGCTGATGATTACCAACAAGAAAAAAAAGTGAACCAGCAGGATGGAGAGCAAGATCAAGACAAGTATAAGAAACAGTTAAAACCTAAAAGGAAGAACCAAAAGGGTAAAAGAGAGCCAAGATTTGCATTCATGACTAAGAGTGAAATTGATCACTTGGATGATGGTTATAAATGGAGGAAATATGGCCAAAAAGCAGTTAAAAACAGCCCCTTTCCAAGGAGCTACTATCGTTGCACAACAACATCATGTGGTGTGAAGAAGAGAGTGGAAAGGTCAATTCAAGATACTTCAATAGTTGTTACAACTTATGAAGGTACACACACGCATTCCTGCCCTGTAATGCCTCGGGGGTATGCTGGCGTTCATCCGGTAACTATCAACTACGGTGTCTCCATTGGCGCTGGTGATAGTGGGGGAAGAACTTATTATGGCTATTACACAATAGAGAGTTTATCCAGTGCGCACAAAGTGCTCACCACAGAGTGCTCACCCGAAGGACGGAGGCTGCGTCAAATATTATAG

> CaWRKY05-3

ATGAACAATCCAAACATGATGAGCAACAACTTTGATATTGAAAAAGATTATTCATTAACCTTTTTATTAGAAAATATGTTTGGTGGTACTCATGTTGATCATCAAGATTATGATTTTATTACAACAACAACTAGTAATTCCATTTTCGATTTGCTAATGTTGCCACCTCATCAGCCTATTATTACTACTTCTACAGTTCAAGATTCAACAATATCTGATCAGTTGATTAATGCTCCAGTGACTCCAAATGTATCATCGATTTCTTCAACTTCAACTGAATTACCTGCTGATGATTACCAACAAGAAAAAAAAGTGAACCAGCAGGATGGAGAGCAAGATCAAGACAAGTATAAGAAACAGTTAAAACCTAAAAGGAAGAACCAAAAGGGTAAAAGAGAGCCAAGATTTGCATTCATGACTAAGAGTGAAATTGATCACTTGGATGATGGTTATAAATGGAGGAAATATGGCCAAAAAGCAGTTAAAAACAGCCCCTTTCCAAGGAGCTACTATCGTTGCACAACAACATCATGTGGTGTGAAGAAGAGAGTGGAAAGGTCAATTCAAGATACTTCAATAGTTGTTACAACTTATGAAGGTACACACACGCATTCCTGCCCTGTAATGCCTCGGGGGTATGCTGGCGTTCATCCGGTAACTATCAACTACGGTGTCTCCATTGGCGCTGGTGATAGTGGGGGAAGAACTTATTATGGTGATTCCTCCTTATTTTCAACTAATAATTTACTACAAGAAAGAAGGTTT

> CaWRKY05-4

ATGTCGTCGCTGCCACCTTGCCGGACGGATTTCCTGACGATACCGCCCGGCATTAGTCCGGCGGCATTGCTTGATTCTACCTTCATCCTCCCAAGTTCAATGACACCTACTGATAATGTTGCACAAATTTTGCATTGCATTGAGTCAACAAACAATCAAATCCCTCAGCAACAACAAGAGTTTACTAAGAAACAAAACCAATTTGAGCAAAGAGGCATATTCAGTAGTAGCATTAGTCCTAACAATTCATCAGATGATGGATACACATGGAGAAAATATGGACAAAAGCATGTAAAAGGGAGTAATTTTCCCAGAAGTTACTACAAATGCACTCAACAAACATGTCCTGTGAGGAAAAAAGTTGAGTGTGCACCAAATGGACAAGTCATAGAAATTGTCTACAATGGTCCTCATAATCACCCGAAAACTCAACATCTTCGACGAAAAGCTATGGATGCAGATTCCTATGTTGTTGGACAAGAAAATGGATCATCATCATCATCATTGATATGGAGAAACGATCAGCAACTTGAGTACAATAAGGATGTTAATAGTTGTTGTAATGAACTAGAAAGAAAACCTTCTGCATCTGTCTTGTCTGATGTCTCATCCGATCCAATGTTGTCGAACAATCTGAAATCGATGAATGTGTTTGAATCAGATGCAACTCATGAGCTTTCTTCTACGCTAAATAGTTTTGATGATGAAGATGAAGATTTGGCCACTCAAGAAGGTAATTTTCTTGGGGATGGTATCAATGAGTTTGAATTTGAGCCAAAAAGAAGGAAGAAAGAAAGTTATTCAGTTGAACCAAGTTTACTGTCGAGAACAGTGAGAGAACCGAAAGTAGTCCTCCAGGTGGAGAGTGAGACCGATATTCTTGAAGATGGCTATCGCTGGAGAAAATATGGCCAGAAAGTTGTCAAAGGAAACCCTAATCCCAGGAGCTACTACAAATGTACGAGCGCTGGATGCTTAGTGAGGAAACATGTCGAAAGGGCATCAGATGACTTAAAATCAGTAATCACAACGTATGAGGGGAAACACAACCATGAAGTGCCATCGGCAAACAAGACGAATGGTGTTGCAGGACATTTTCGATCAGCATCTATGTTGAACAACGGGCAGCAGCAGCCGGCTTGCACAGCATCTCGTAAGAGTCTTAAAGATTCAAATATCAGAGTGCAATTTCAAGATCTACCTATTCCGTTCGAAAGAAAGTTTTTCATGGGAAGTGAATATTTAAGGCCAAATTTTGGTGCAAGTTACCTCAGTGACTTGAGTTTCGGAGGTGGTTCTTTGCAGCTCCCGGACTTTGCAATACCATTACCATTGCCATCGCGAATGAGTTTTCCTGCAAGACAGAACGAGCCTCGTCTTGGTGACTTTCAGATGAATAATCATCTCTTGCTGCCAAATGGTGCATCGACATTTTTAGCAGCTGGGAATACACGACATATTAACGATGACAACAATAACTCGAGACTACTTAAAGCCAAAGACGAGGTCCAGTACTGGACCTAG

> CaWRKY06-1

ATGGAGGAAATAGAAGAAGCGAATAATGCTGCCGTTGAGAATTGTCATAGAGTGATGAGTCTTTTATCGTCTGGGACACACGATCGGAATCAGTATATGAATTTAGTTAGAGAAACTGGAGAAGCTGTAAACAAGTTCAAGAAAGTTGTCACTCTTTTGAATTCAACTTTAGGTCATGCAAGAGTGAGGAAATCGAACAAATTCAAGACCCCTTTACCTCATAACATCCTTGTAGAAAACCCGAATTGCAAAATCGATGATCAGGCGAAAGCTCTTAGGTCACGTCCTATCGATGAAAATCGAGTCCTAGAGATGGGGGGTACTAATGTGAAATGCAATCTTACTTTAGGAAGCCCTTCTTTGGAATTAAGTTCAAATAGTAGAAATCCCCTTAATTTTGGCCAACAAACGCATTTGCCGAGCTACAACTATCTTCAACAGCAGCAGCAGCAACAACGGCTGTTTCTACTTCAGCAGCAGTCTTTTATCTCCTCATTGAGTGTGGACGGTAGCGTTGCTAATGGTAGTAACTTTCATTTAGTTGGGGCTTCACAATCTTTGGATCAGAGCTCGTTCCAACACAAGAGAAGGTGCTCGGAAAGGGGAGACGAAGGAAGCGTGAAATGTGGAAGCAGTGGGAAATGCCACTGTTCAAAGAAGAGGAAACACAGAGTGAAAAGATCAATCAAAGTACCTGCTGTAAGTAACAAGCTAGCTGATATTCCTTCCGACGAGTATTCTTGGAGAAAGTATGGACAAAAGCCGATCAAAGGTTCTCCGCATCCTAGGGGATACTATAAATGTAGCAGCATGAGAGGCTGCCCTGCCCGGAAACATGTTGAGAGATGCTTGGAAGACCCTTCAATGCTTATCGTGACTTACGAAGGTGAACATAATCATCCCAGAATGGCATCACAATCAGCTAATACCTGA

> CaWRKY06-2

ATGACGAATCTAGGAGCAGCAAAGAAGATTCTTGGCATGAAAATTATGAGGGATAGAGAGAAGTGTAAGTTATACTTGAGTCAGAAAAATTATATTGAGAAAGTGCTTCACAAGTTCAATATGCAAAATGCCAAACCTATTTATCTTATGCCGCCCGTGCAGTTAGCAGATATATGGCAAATTTTAGCAAAGAACATTGGAAAGCAGTTCAGAGATCATGATAAAAGGAGATCCCTTACGGCTATGTTTTCACCATTGGTGGTTGTGCTATTAGTTGGAAAGCTACCTTACAGACTACGATTGCTTTGTCAACTACTGAGAATGGGCGATACAGACAGAAGAACAGTGAGAGTGGCTGCTCCAAGAATGGGAAATCTCGAGCTCCCACCTGAGGACGGTTATACTTGGAGAAAATATGGTCAGAAAGAGATTCTTGGATCTAGGTTCCCTAGATGCACCCATCAAAAGCTATACCATTGTCCAGCCAAGAAGCAAGTTCAGCGTCTTGATAATGATCCTTACGTATTTGAAGTAACATATCGATCTCAACACATTTGCTATATGTCCGCCACAGCTCCCACCGTGCCTCCACCATCTGTGGAAGAGATAACTCATCAAACCACCACAACTCCTCCACCGCTGCTGCCACTGCCGCCACCAACTTCAGCCTCCTTAAGCGGGCATTGGCTCTCCATGGATATTAAGCCACAAGTAGAAGCTGGCACAAGCTACAGTACCGCTCAATTTGACATACAAAGGGATTTTGGACATGCTAGTGGTGGTTCACTAACTAGCATATGCAATGTTGTGACCGCCTGCGTGGACGGTGGTGCGGGCCCTTCCGGTAGTAGATTTGGAAGAGAAGTTGACTACCAGCCAGTGGTGGATATGGCGGACGCCATGTTTAATTCTGGGAGTAGTAGCAATACTAGCATGGACATCATCTTTTCTTCTATTGATGATAAATGGGACACGACACAAAAGAAAGAATAG

> CaWRKY06-3

ATGGAGGAGATTGAGGAAGCTAACAGGGTGGCAGTTGAGAGTTGTCATAGAGTTATTACTATGTTATCACAACCCCATGATCAAAAACAGTTTGGAAATGTAGCAAGAGAGACTGGAGAGGCTGTACACAAGTTCAAGAAAGTGGCGACACTTCTAAATTCCAATTTAGGTCATGCAAGAGTAAGAAAGGCCAAGAAAATTATAACCCCTTTACCTCAAAACCTCTTGTTAGAGAGCCCAAGTTGCAAAACTTATGATCAGCCTAAATCCCTACAGTTACTGCCTATCACAGAAATTGGTTCTAATGTGAAAAGCACTCTTACTTTAGCCAACCCTTCACTAGAATTAAGCTCACATAGTAAAAATCCTCTTCAGTTAGCTCAACAAACACCGTTGTCAAGCTATCACTTTCTCCAACAACAGCAACAGAGGAGGTATCAACTTCAGCAGCAACAGTTAAAGCAGCAGACAGATATGATGTACCGGCGAAGCAATAGTGGGATTAGCCTAAATTTTGATAGCTCTACTTGTACGCCAACTATGTCATCCACTAGATCGTTTATTTCCTCTTTGAGTATTGACGGAAGTGTTGCTAACTTGGATGGTAATGCAAATGCCTTCCATTTTATTGGGGCATCACGCTCTGCGGATCAGAGCTCATTTCAACACAGGAAAAGGTGCTCAGGGAGGGGAGAGGAGGGAAGTGTGAAATGTGGAAGCAGTGGTAGATGTCACTGTTCCAAGAAGAGGAAGCACAGGGTGAAGAGGTCAATCAAGGTGCCTGCTATAAGTAACAAGCTAGCTGATATTCCCCCGGATGAGTATTCATGGAGAAAGTATGGACAGAAGCCAATCAAAGGTTCTCCGCACCCTAGGGGATACTACAAGTGTAGCAGCATGAGAGGCTGTCCTGCCAGGAAACACGTTGAGAGATGCTTGGAAGAACCTTCGATGCTTATTGTCACTTACGAAGGAGAACATAACCATTCTAGGTTGCCATCTCAATCGGCAAATGCATGA

> CaWRKY06-4

ATGGCTGCTTCAAGTTTCTCATTTCCCACTTCTTCTTCATTCATGACCACTTCTTTCACCGACCTTCTTGCTTCTTCAGATGATTATCCTATTACCAAAGGACTTGGTGATAGAATTGCTGAGAGAACTGGTTCTGGAGTTCCTAAGTTCAAGTCTCTACCACCTCCTTCACTTCCTTTATCTCCTCCTCCTTTTTCGCCTTCCTCTTACTTTGCTATTCCTCCTGGTTTAAGTCCTACTGAACTCTTGGACTCACCTGTTCTCTTGTCATCTTCAAACGTTCTTCCGTCTCCCACAACAGGGAGTTTTCCAGCTCAGGCTTTTAATTGGAAGAGCAGCAGCAACAATCAGGATGTTAAACAGGAAGAAAAAAACTGCTCTGATTTTTCCTTCCAGACACAAGTAGGGACAGCTGCATCAATATCTCAATCCCAAACTAGCCATGTCTCTTTGGGGCAGCAAGCATGGAATTATCAAGAGCCCACAAAGCAGGATGGTCTATCATCCGATCAAAATGCTAATGGAAGATCTGAATTCAACACTATGCAGAGTTTTATGCAGAATAATGATCATAGCAATAGCGGGAACGGATACAACCAGAGTATAAGGGAGCAGAAAAGATCAGATGATGGGTACAATTGGAGAAAATATGGGCAGAAACAAGTAAAAGGTAGTGAAAATCCAAGAAGTTACTACAAGTGTACATACCCAAATTGTCCCACCAAGAAGAAGGTTGAAAGATCATTAGATGGCCAAATTACTGAGATTGTGTATAAGGGTAATCACAACCACCCAAAGCCTCAGGCTACCAGAAGATCGTCGTCATCCACAGCTTCATCTGCAATCCAATCTTACAATACACAAACCAACGAAATCCCAGATCATCAATCCTATGGTTCAAATGGTACAGGACAAATCGATTCAGTTGCAACACCTGAGAATTCTTCTATTTCATTTGGGGATGATGATCATGAGCACACTTCTCAAAAGAGCAGGTCAAGAGGAGATGATCTTGATGAAGAGGAACCAGACTCGAAAAGATGGAAAAGAGAAAGCGAAAGTGAAGGTCTATCTGCACTTGGGAGTAGGACAGTTAGAGAACCTAGAGTTGTAGTTCAAACTACAAGTGATATCGATATCCTAGATGATGGTTATAGATGGAGGAAGTATGGTCAAAAAGTAGTGAAAGGAAATCCTAATCCCAGGAGCTACTACAAATGCACAAGTCCAGGATGTCCAGTAAGAAAACATGTGGAAAGGGCATCACAAGATATAAAGTCAGTGATAACAACCTATGAAGGGAAGCACAACCATGACGTTCCGGCAGCTAGGGGCAGTGGAAACCACTCAATTAATCGACCTATTGCCCCGACCATTACGAACAACAATAGTGCCATGGCCATAAGACCCTCCGTGACATCTCATCAGTCCAACTATCAAGTTCCAATGCAAAGTATAAGGCCACAACAGTTTGAAATGCGAGCACCCTTTACGCTAGAGATGTTGCAGAAGCCTAATAATTATGGTTTCTCAGGATATGCCAATTCAGAGGATTCATACGAAAACCAACTTCAGGACAATAATGGGTTTTCTAGAGCTAAGAACGAACCTCGAGATGACATGTTTATGGAGTCATTGCTTTGCTGA

> CaWRKY06-5

ATGGAATTTACCAGTTTGGTTGATACTTCATTGGATTTGAGCTTTAGGCCTCGTCCAGTTCTTGATAAATTGCCGAAACAAGAAGTTCAGAGTGATTTCACTGGATTGAGGGGAGACAATATGGGGGTGAAAAATGAGACAGTGGATTTGTTAGAGGAACTAAATAGAGTGAGCAGTGAAAACAAGAAGCTTACTGAGATGCTCACAGTGGTTTGTGAAAATTACAATGTTTTAAGAAACCAAATGATGGAGTATATGAGCACACAAAATGGTGTGGCAGATGATAGTGCAGGGTCAAGGAAGAGAAAAGCTGAAAGTATCTCCAATCCCAACAACAGCAACAGCAACGTCAACATCAACAACAACAACAACAACTTGGATGTTGTGCCTGGACGTTCATCAGAAAGTAGCTCAAGTGATGAAGAGTCTTCTTGCAAGAAACTTAGAGAAGAGCACATAAAAGCCAAGGTTACAGTTGTTTCTATGAAGACTGATGCATCTGATACCTCTCTTATTGTAAAGGATGGTTATCAGTGGAGGAAGTATGGTCAGAAAGTAACAAGAGACAACCCTTGTCCAAGAGCTTACTTTAGATGCTCATTTGCACCTACCTGTCCTGTCAAGAAGAAGGTTCAGAGAAGCATAGAAGATCAGTCTATTGTGGTGGCAACATATGAAGGAGAACATAACCATCCAATGACCTCAAAACCAGAAGCAGGAGGTGCAAATACTACTAGTACTTCCACTGGCAGCCGGTTAAATGTGACGACTATCGCGGGTACTACTGCTTCAGTACCTTGCTCTACAACTCTCAATCCTTCAGGACCAACCATTACTCTCGATCTTACTGCACCGAAAACAGTAGAAAAACGCGATATGAAGATGAATCAGAGTGCTAGTCCTACCGGTGGCAATAGCATTCATACATCAACAGGAGTTGAATATCAAAATAGGCCAGAGTTCCAACAGTTCTTGATAGAGCAAATGGCTACTTCCTTGACCAAAGATCCAAGCTTCAAAGCAGCACTTGCTGCCGCCATATCAGGAAAAATCCTCCAACATAATAATCAGACGGGCAGATGGTAA

> CaWRKY06-6

ATGGAGAACAAGAACAAAGCTGAATATTATAGTCCTGATGATGAAGATCAAGAAAATATTATCCATAAGTTTGGAAAGGGAAGAAAAGAACGTGAGGATGATAAGTCAAAGCCATCCTCCCCTCACCACAAGGATTTCATGGCAATTGACAATAATATTAAGGGAGTTGCTGTTAATGTTATGGTCAAGAGAGAAAGATCACCCCCAGAACTCAACTCAATGGCTTCTTCATCTGCTCACAAAGAAAAGGATGATCAGCTTGCATTAGCCAAAGTTGAAATGCGAGAAGTAATGGAAGAAAATCAAAGGCTTCGATTTCACTTGGATCGAATCATGAAGGAATATAGAAATCTGCAGAATCAATTCCATGATATCGTTCAAAGAGAAGTTGATCAAAAATCAAGCAGTACAGTGAACACTACTCAACATGAATCTGATCACGAAACAAATGAACTTGTTTCCCTTAGCCTAGGAAGGGCAACGAGTGATATGAAAAAAGAAGAATTATCAAAAATCTTGAAGAAAGATAAAGGTCGTGATGATGAAGATGTTAATAAAAGTCTTGATTTAGGTTTGGATTGCAAGTTTGAAGAATGTTCGCCTGTGAAGAATCGTAGTCCAGAGAATAGCTTAGATGATCATCAAGCTAATAAGGACGAAAATGGAGAGACGTCGACGACCACGTGGCCTCCTAACAAAAATCTGAAGACGATGAGGAATGATGGAGACAATGGTGATGATGTTTCACAACAAAACCCTACTAAAAGAGCTAGGGTTTCTGTTAGAGTCAGATGTGATGCACCTACGATGAATGATGGATGCCAATGGAGAAAATATGGTCAAAAAATTGCAAAAGGAAATCCATGCCCTCGAGCTTATTATCGTTGCACTGTAGCGCCAAATTGCCCAGTTAGAAAGCAGGTTCAAAGATGTGCTGAGGACATGTCAATATTGATCACCACATATGAAGGAACACACAACCACACACTCCCTCTTTCCGCCACCGCAATGGCTTCCACCACCTCTGCTGCCGCTAACATGTTGTTATCCGGTTCGTCGAGTTCATCAGACCCAAGTCCACAAATAACTGCCACCACTACCAACACCGCCACCGCTACTACTTCTGCCAATATCAACGGACTCAACTTCTATATCTCCGATACCTCAAAACATAAGTCGCCTTTTTACTTTCCTAATTCATCCATCTCAGCATCCACACTTAATAATTCGCACCCTACAATCACTCTCGATTTGACTTCTACGTCCTCCTCCTCATCCTCGTCCTTATCTCATCTTAACAGGATGAGTAACAATCTCCACCCTAGATATAATTACAATAACTCCTCCACAAACCTCAATTTTAGTTCAGTGCTAGAATCCAATTCCCTTCCCATTTCTTGGACAAATTATCAAAACCAAACCTGCAACAAGAACAACCAAAATTTTGGTTCACTTAACTTCTCATCAAGACCAAATCAAGAAAATATTTTCCAGTCTTATTTACAAAAAAACAACAACATTATCCCTACACAATCTTCTTTTCCACCAGATACAATTGCAGCTGCAACGAAAGCAATAACGTCAGACCCTAATTTCCACTCAGCATTAGCTGCTGCTCTCACCTCAATCATTGGAAATACTGGAATCGAAAATAAGCCTGGCCATAATTTCAATGTTTCTGAGCCATTTCCAGTTTTGTCCAGTCTCCCATCAAGCTCAAATCCGAATAAATGCTCGTCAAGTTTTTTAAATAAACCAACTTCTTCTTCCGCGAATAATAGTTCACAGCAGCCTGGAAATAATAACAACTTGGTGTTCTTCGCGCAATCTTCTTCTTCTTCATTGCCATTTTCTACGTCCAATAAGGGTAAATCTACCTCTCCTAGTGATAGCTAG

> CaWRKY07-1

ATGTTTAGCATTGGGTTTACTACCAATCATGATCCTTTGGTTTTGGATAAATTGAAATCAGAGGTCAACTTTAACAGGCGTGATATGGATAGAATTAGTGTTACCAGGCCTGGAACTATGAATGCTCAAGCACGAACAAAACATCAACAACGTGTGCCAGATGAATCATCCACATTGGAGCTGTCCTCTACATCTGTTGCACAGTCCATTTCATCAGTGCCAAGTCCAACTCTAGCAGAAAGCCGGTTGTCAGCAGTAGTAAATTGTGGTACAGGAGAAGTGGCTAAGCAGAGCTCCGATGCCAAGGTTCAACCTCTTGTACCAGTAAAGACATCAAACCGTGATGGGTACAACTGGCGGAAGTATGGTCAAAAGCAAGTTAAAAGTCCTCAGGGTACTCGAAGTTATTACCGATGCACTCATTTCGAGTGTTGTGCCAAGAAGATTGAGTGCTCTGGTCACACTAATCGTGTAATGGAGATTATTTATAGAAGTGAACACAATCATGATCCATCCCCGAGCGTAACTTGCTCAAGGGAAAGCAAGTCTGCAATATTGTCTGCATCTACCAATGGCAAAAGTTTAATAGATCATCCAAATAGAAATTCTAATGAGACTGTGGCATCCTCTTTCAAAGAAAATTTACAAGAAAGTTTACCAATCGCCGAGACAGCAAATTTGGATTCCGGTGGATCCGACACTGACACTGAAATCAATATTAAAGAGGAGCATTGTGACGAACCTGAACAAAAGAAGAGGTCAAGAAAAAGTGACGCGAGTTGTTACGAATCTGTTTCTAAACCTGGAAAGAAACCCAAACTTGTGGTGCATGCTGCTTGTGATGTAGGAATCTCAAGTGATGGCTACAGGTGGCGAAAGTACGGACAAAAAATGGTGAAGGGAAATCCCCATCCAAGGAACTATTATCGTTGTTCATCAGCTGGATGTCCTGTTCGAAAGCACATTGAGAGGGCTGTAGATAGCACAATCGCGCTAACAATAACCTACAAGGGAGTACACGATCATGACATGCCCGTACCAAAAAGGCGTCATGGTCCACCAAGTGCACCTCTTATTGCTGCTGCTGCCGCCCCAGCTTCCATAACCGATATGAAGAAACCTGAACCACTACAACATCAAAAATCGACCACACAATGGTCCGTTGATAAACAAGGTGAGTTGACAAGTGAGAAATTGGATCTTGGAGGAGGTAAGGCAATGGGATCGGCTCGAACTCTGTTGAGTATTGGATTTGAAATAAAGCCTTGCTGA

> CaWRKY07-2

ATGGGCATATCTGAGGATGATATGACGGATGAAATTTCCTCCAGAAAATTAAAGCAGAAACAGGACCCTGACACTGTCATCAACGGATCAGAGTCGAAAGAGAAAGGAACATGTGAATCAATGTCAGCTGAAGTTGTTTCTAATGAACTACATAAAAGACCGAACCCTGATGCTTTGGCAAAGGTATCACAAAGTAATCGAGATGAAAGTGCACATCCTACGACATGTCAAGGAGTATTGAATGAAGGCCAGCCAAGGAGGAATGTCGATAAAGAGATCGATGTGTCACAATCCAATCAAAAAGATAGTTCCCTTTCTAATGTACCAGAGGAAAATTCAGAAAACGTGCATCAGGAAAAGGGTCCAGAGAGTGAGGGTGGTGCATCAGAATCTAGTCGAGTTTCTGTCCTACCAAAGGAGGAACCATACATAAAATCTTGTAAATCAGATTCTCCTGTAAAAGAGGGGAACATCTCCCTGGTAGTTGGAACGGCTTCAGATTGTTCACCACAAATACAAAGTAAAAAAATGGAAGAAGTTGTATCTCAATCTCATCAAGAACGAGTAACTTATTCAACAATGGCTGAGAATGCTATGTATAAATTGCGACCAAGGCGGAACCCTGATACGAGTGTCCAGGATTTGCCATCTGATGAAGGAGTCAGGGATTTGCCATCTGATCAAGGAGTCACTCCCTTCAGTGAACCTGAAAAACCATTTGAAGATGGATATAACTGGAGAAAATATGGTCAGAAACTTGTTAGAGGAAATATGTTTACTCGGAGTTATTACAAGTGCACACACTCCAATTGTCTAGCAAAAAAGCAAGTGGAGAGATCACATGATGGGCATATTACCAATATTCAGTATATAACAAACCATGAACATCCAAAACCTCTAAATAGTCCCCAAATCTCCCCTGAGGTTGTAGTGCCTTCGGAAATGAGACGACCAGACATGCTAATGGGCACACCACAAGCTGAAGGTGAGAAATCTACTGCACTTGGTCAAGCATGTGAATCTATCGAACCATTGGAGAGCCTCATTTCAGCGGCTGTTGAATCGGCTGGTGGTAGCGCACGAGATACTGTCCCAAAGTCACTTAAATCAGGAGATGAGGGTGATAGTAATGGTGGTCGGAACTCAAAGAGACGGAAGAAAGAAGTACCTAGGAGCGATGACATGACTCCACCTATGAAGTCTCATAGTGAACCACGACACATCGTTCAAACTAGGAGTGAAGTGGATATACTCAATGATGGTTACCGATGGCGTAAATATGGGCAAAAATTTGTGAAAGGAAATCCAAATCCTAGGAGTTACTACCGATGCTCGAGTGCTGGTTGCCCTGCAAAGAAGCATGTGGAGAGGGCATCCCATGATCCAAAATTAGTGATTACAACATATGAAGGGCAGCATGAGCATGACATCCCACTTTCCAGGACTGTTGCGCAAAATTCGGATTCTAATACAACCAGAATAAGTGGAGAGTCCACAGCTGAATCAGGTGGAAACAAACATGTTGACAATTGA

> CaWRKY07-3

ATGGCCAAAGGTAGTGGACTCTCTTTTGATCCAGATCCCATCAAACACTTCCTTCCTATTCCTACTGTCCTCAATTCTTTTCTTGAACCCCACCACCAACAACAACAAGAATTTCCACATAACAAGTTTTTATTCAAGATTGAACCTTTGTCATCCTCAATGGAGTCATCAACATTCAAGAATAGGTCACCACCTTCTACTATTCAGTTCCCAGTGAACCTTAACTGCTCTACTACTGCTATTCATCATGATCACCAAGAAGAAGAAGAAGAACATAATAACAGACCAGTTATTGATGAAATGGACTTCTTTGCTGATAAAAAAAATGGTAATAATTCTGAGGAGGCTGATGTTACAACAACAACCAATAATACTATCAATCACTCTGATAGAAAAGATTCCAACACCCCTCCTCCTGAATTGGATTTTAACATTAATACTGGTTTGCATCTTCTCACGGCCAATACTTACAGTGATCAGTCCATAGTGGATGATGGTTTATCCCCTAATTCTGAAGATAAAAGAACCAAGAGTGAGTTAGCAGTTCTTCGGGCTGAATTGGAAAGGATGAACGGTGAAAATCGACGTTTAAGGGACATGTTAAATCAGGTGACAAGCAATTACAGTACCCTGCAGATGCATATGATGACAATGATGCAACAACAACAGCAACAAAATCAAGAAAATGGTCAACGTGATGGGAAAAGTACTCGCGAAGAAGTGAAACAGCAACACCATAGCCATAATAGTCATGGAGGAGGAGGAGGACAAATGGTGCCTAGGCAATTTATGGATCTTGGCTTAGCTGCTGCTGGTGCTACTGGTTCTGAGGCTGAAGAGGCTTCCCAGTCTTCGTCAGAGGGACGAAGTGGCAGGGAAAAATCGCGATCACCAATGAATAACATGGAATCACGTTCCACATGTGGAATTGGAAGAGAAGATAGTCCTGAAAAAGGGTCACCTGGTTGGGGTCCTAATAAAATTCCAAGACTTGGCAATGCCTCTACTAATAAACCTGCTGATCAAGCTACCGAAGCTACCATGAGAAAGGCTCGAGTCTCGGTCAGGGCTCGATCAGAGGCTCCCATGATCACAGATGGTTGCCAATGGCGAAAGTACGGACAGAAAATGGCGAAGGGAAATCCGTGTCCTCGGGCTTATTACCGGTGCACCATGGCAGCTGGTTGTCCAGTTCGGAAGCAAGTTCAAAGATGTGCAGAGGATAGAACAATCTTGATCACAACCTATGAAGGGACACACAACCATCCGTTGCCTCCGGCAGCGATGGCAATGGCCTCAACAACTTCCTCAGCAGCACGAATGTTGCTGTCGGGTTCTATGCCAAGTGCAGATGGGCTAATGAATTCCAATTTCTTCGCGAGAACTCTCCTCCCTTGCTCTTCCAGCATGGCCACAATTTCAGCCTCGGCCCCATTCCCTACTGTTACATTGGACCTAACTCAATCCCCAAACCCGTTGCAATTCCCAAGACCCCCTAACCAATTCCAAGTCCCATTTTCCAATCCACCTCACACTAACATCCTAGCAAATCCAGCTGCACTTTTGCCTCAGATATTTGGCCAGGCTTTGTATAACCAGTCCAAATTCTCTGGCCTCCAATTGTCCCAAGATTTGGAGGGACAACAACATCCTTCAACGATGTCATCATCGATTCATCCATCCAACCACAACCCTCTGGCTGACACGGTGAACGCCCTCACCAATGATCCTAATTTCACCGCAGCATTAGCAGCAGCCATCACTTCACTTATTGGAAATCCCGGGCAATCAAATAATACCCCTGCCACCACAACGACCGCGAACAACAATAATGGCAGTGTTACAAGCAATGGCAATAACAGCAACAATGGCAATAATAAAGTGGCTAATTCAGGTTTTCCAGCAAATTGA

> CaWRKY07-4

ATGGAAGATAGGCTATACAAAAGTTCATTTTTTCATAAGCAAGAAGATTCCACCGGAACTCCGCCGGATAATGCTGCTGATTCTTGTTTTTCCGGTGATGAAGCGGCTGAAGTTAACATGCCATCACCTAGAAAAAGTAGGAGAGGAGCAAAAAGGAAAGTAATATCAGTGCCAATAATTGAAGCTGATGGATCAAGGAGTAAAGGAGAAGTTTATCCACCACCAGATTCTTGGTCTTGGAGGAAATATGGACAGAAGCCAATTAAAGGTTCACCTTATCCCAGGGGATATTATCGATGCAGTAGTTCCAAAGGCTGTCCCGCCAGAAAACAAGTCGAACGTAGCTGCCTCGACCCCACCATGCTCCTCATCACCTACTGCTCCGATCACAACCACCAACTCCCCGCCGCCGCCGCCACCGCCACCAAACACCACCATACCGCCGCCGCCGGCGCTACTTCACCATCCACCTCCACCGGCACCGCCGTAGACATCAACCCTTCCGCCGCCTCCGACACGGCCAAAAAATCGTCACCGGAAGAGCAAGAGACGAATATCTTTGCTGGATTCTCGGAGTTCGCCGGTGAATTAGGTTGGTTTTCAGATATGGGAACCAGCACGTTAATGGAGAGTGCTTCGACTTCGGCAACATCCATAGTGGGGTCCACATGGAATGATAGTGACGTGGCATTAATGTTGGCGATTCGGGAAGAGGACCAGTCATTGTACGGTGATCTCGGTGAGTTGCCGGAATGTTCGTTTGTTTTCCGGCGGTATAGTGTCGAAACTACCTGTTGTGGCGGTACAGGATAA

> CaWRKY07-5

ATGTCTGATAATAACCCATTTCATCATGATTATGCATTTCCTTTCTTTGGTGAAAATCCCTCAATTTATGATCATCAAGTAGAAAACACACAAAACCCTCATCAAGATTTTGATTATCCTTCATCTTATATGAGTAGTCTCACAGAGTGTTTACATGGGGGTTCAATGGATCATTACAACTCTTTATCAAGTGCTTTTGGCATGAATCATCGTACATCATCTTCTGAAGTTGTTTGTCCACCACCAATAGATCATCATCATCATCAAGAACTTTCTAGAAAAAATAGTGTTGATCATCATCATCAAATTCCATTGACACCTAACTCTTTAATCTCTTCATCATCTAATAGTGAGCCTGGATGTCATGAAGAAGATTCTTCAAAAATCAAGAAAGATGATCAGTGTGAAGATGGAGGTGATGATGACAAGTCTAAGAAAGTGAACAAAGCAAAGAAGAAAGGAGAAAAGAAGCAAAAGGAACCAAGATTTGCATTTATGACCAAGAGTGAGATTGATAATCTTGAAGATGGCTATAGATGGAGAAAATATGGACAAAAGGCAGTGAAGAATAGCCCTTTTCCAAGGAGTTATTATAGGTGCACAAGTCAAAAGTGCAGTGTGAAGAAACGTGTGGAAAGATCATATCAAGACCCATCAATCGTGATCACTACATATGAAGGCCAACATAACCATCATTGTCCAGCAACCCTTAGAGGCAATGCTGCTGCTGCTTTGTTATCACCAGCTTCATTCTTATCCTCCTCACAACAACAATTATTTCACAATCCAAGTGAACAACAACTTTTCTATAATCCAAATCTCCCTATTAATAATTCTTTCTACAATAATTATCATCAACATCAACACCAAATGCAGCCACAATTAGGTCCTGATAATTATCAGTATGGAGTATTTCAAGATATGGTTGCATCATTGATCCACAAAAGAGAGCCATGA

> CaWRKY07-6

ATGGCAAACTCCCACGCCGAGCAGCTCACTAATGGCAGAACCTCCGCTTCCTCCGCCGCAGCTCCCGGCGGATCGAACGGAGGCGGAGCCGTAGCAAAGTACAAGCTGATGACTCCAGCTATGTTACCGATCTCTAGGTCCACATGTATCACTATACCTCCCGGTCTCAGTCCTTCTTCCTTCCTCGAATCTCCTCTTCTCCTTTCTAACATCAAAGCTGAGCCATCTCCGACTACAGGTTCCTTCTCCAAATTTCAAACAGTGCAAGGCTCTGGTGGGGCTGCTGCATTCTTATTGACGAGAGGTTATTCTAGTAGCAATTCATACATTGAAAGAAAATCCAGCTGCTTTGAGTTCAAAAATGCCAGTGGATCTAGTTCTACATCAGGATCGTTTGCAACTGAACATGTGATTTCTACAGGTTTCAACCAACAACAAAATGATCCACTGAAAGAAGTTCAAGATCAAAGTCATCGTCAATTGTTGGTACCTTCATCTCTAGCTAAACTCGAGATGGAATCATCAAAAGAACTGAGTATATCTGCGCCTGTTAATGTTGATGCTTCATCAAAAGAAGAGAGTCTATGTCAACCTATTAATGTTGATGCCATGAATCCGGGAGGTCCATCTAATGCAAGCATGCAAGGTTCACATGCCGATCATAAAGATGTATCATCAGTAACATCTGAGAGATCATCAGATGATGGGTATAACTGGCGAAAGTATGGCCAGAAACTTGTTAAAGGAAGTGAATTTCCAAGAAGCTATTACAAATGTACATACCCAAACTGTGAAGTGAAAAAGATATTTGAGCGGTCACCTGAAGGACAAATAACAGAGATTGTTTATAAAGGTTCCCATGATCATCCTAAACCCCAACTGTGTCGCCGATTTTCTCCTGGTTCTCTCGTGTCTATCCAAGAAGATAAATGCGAGAAAGAAGCGTGTTTCAGGGGTCAAGAAGTCTATGTTGAAGACAAGTTCAACACCAATGTCCAAACTAATAAAATTGAGCCTGGTAGTACCCCTGTATCACCTCAAACGGACACTGATGCTCTTGAAGGAGCAGCGTCACAGATGCAAGGCACTAATGATGACATGGATGAGGATGATCAATTTGCGAAAAGAAGGAAAATGGATGGTGGGATGGATGTAACACCAGTGATAAAGCCTATCCGTGAACCACGCGTGGTTGTTCAAACAGTCAGTGAAGTTGATATATTGGATGATGGGTACAAGTGGCGCAAATATGGACAGAAGGTGGTCCGAGGCAATCCTAATCCCAGGAGCTATTACAAGTGCACCAATGCTGGATGCCCTGTCAGGAAACACGTGGAGAGGGCTTCTCACGATCCCAAAGCTGTTATCACCACATATGAAGGAAAACATAATCACGATGTACCTACTGCGAGGAATAATAACCATGAAATGACAGGATCCACGCCTGTAACTGGCGGTTCAAGGATCAGGGCAGAACAGACCAACTCACTTAGTCTGGATCTAGGTGTCGGTACTGGATATCATCTGGACAATGGGAACAACGGGCAACTTCACACCCTCCATAACCAAGTTCAAGTTTCACGTTCTGGTATGATGCTAGTACAACCAGGTGCAGTTGTAGCGCGATATGGTATTGTACATAATGGCATGAGCCGATTTGGAGCTATAGATAATCGTGTTCAAGGACCTAGTTTCGAAACTTTACCTTTACAACCTTCTACCCAGTCTCTCCAAAGCTACGGAAAGATACTCCTCGGCCCGTGA

> CaWRKY07-7

ATGGGTGGATTTGATGATCATGTTGCCATTATGGGAGATTGGATGCCTCCAAGTCCAAGTCCAAGAACTTTTTTCTCGTCGCTGCTAGGTGATGATGTTGGGTCAAGATCAACTTTTCAGTGTACCAATGAAACTAAAAGTGGAAACTTAGCTTCTGGGCCTCAAGAAAACGTGGGAACTTTTGATGGAAATGATGAAGCACAAGCTGCAGTCAGTGAACAACAACCGGTGTCTGATCAGAAAATGAACCCTCGTGGAGGACTCTTGGAAAGAATGGCAGCTAGAGCTGGATTTAATGCTCCAAAGCTGAACACGGAGAGCCTTAGACCTGCTGATATGAGGCAGAATCAAGGAGTTCAGAATCAAGGAGTTCGGTCTCCTTATTTAACTATTCCTCCTGGTCTTAGTCCAACAACCTTGCTAGAGTCTCCTGTTTTCCTCTCAAATTCACTGGTGCAACCATCCCCAACCACTGGAAAATTTCCATTTTCCTCGGGCATCGAGAGTAGAAACTCAACATTGATGATGGAGGATCCAGATAATAGGAAAGAGAATGCTTTTGAGAGTATCAATGCGTCGTCCTTTTCTTTCAAGCCAGTTCCAGAGACTGCTCCATCGCTTTTTCCTGGCACGAACAGCAGATCTTGGTTGCAGGTGAACCCGCCCAATTTTTCTCAGCAAGGATTTCCAAACATTGAAGTTTCAGTTCATTCACAGAACTCGCTTCAATCTCACCGTATGGAGGCTACACAAAATCTGGTTCAGAATGGAACGCTTAATCAAGCATCTGATTTCCCTAGATTTTCTGCTGAGATGGATGTCAAGGGTAGTAATGTCACACCAGAGTCAAGGACCTTTCAGACGGTTGGTAGTACTGTGGAGCATTCTCCACCTCTCGATGAGCCGCAAGATGAGGACATTGATCAAAGAGGAGGTGGAGATCCAAATGTTGTTGGTGCCCCAGCAGAAGATGGGTATAACTGGCGAAAATACGGGCAGAAACAAGTTAAAGGGAGCGAGTATCCCCGGAGCTACTATAAGTGCACACATCCAAACTGTCCAGTGAAGAAGAAAGTTGAGCGATCTCAAGAGGGTCATATTACTGAGATTATATACAAGGGAGCCCACAATCACCCAAAACCGCCACCTAACCGTAGATCAGCCCTTGGATCCACAAATTCACTTGGTGACCTACGGCTGGACGGTGTAGAACAAGGTGCAAGTGGTGTTAATGGTGATCTGGGTCGGGCAAACATCCAGAAAGCACCTGGTTCTGGAGGAGGTTTTGATTGGAGGAGCAACAACCTTGACGCAACATCATCAGTGAACTTGGGCTCCGAGTACTGCAACAGATCTGCCCCTTTCCCTGCTCAAAACGACAGTCAGTTGGAATCAGGGGATGCAGTAGATGTGTCGTCGACTTTTTCAAATGATGAAGATGAAGATGATCGTGGAACTCATGGCAGCATATCACAAGGTTATGATGGTGAAGGAGATGAGTCCGAGTCTAAAAGAAGGAAGCTCGAGACTTACTCTGCAGATATGACTGGTGCCACTAGAGCCATCAGAGAGCCAAGAGTTGTGGTTCAAACTACAAGCGAAGTGGACATCCTTGATGATGGATATCGCTGGCGCAAGTATGGGCAAAAGGTTGTTAAAGGGAATCCAAATCCAAGGAGTTACTACAAGTGCACAAGTGCTGGCTGCAATGTCAGGAAGCATGTTGAGAGGGCCTCACATGACCTGAAGTCAGTGATTACCACCTATGAAGGGAAGCACAACCACGATGTTCCTGCAGCTCGCAACAGTAGTCACGTTAATTCAGGAGCCTCCAACACTCATCCAACTGCAGTAACTGCCCCTGCTCAAAACCATTTACATAGGCCTGAGCCTGCACAACTTCAGAATGCCATGGCGCGGTTCGACAGGCAACCTTCACTTGGCTCCTTTGGTTTGGCAGGGAGGCCCCAGCTTGGACCTACCCCAGGATTTAGCTACGGAATGAACCCACAAGGCCTATCCAGTCTATCAATGGCTGGATTTCACCCTAACCAAAACAAGCCAGGGGAGGTTCCTATTCATCCATATCTAGGACAGCCGCGACCCATGCATGACATGGGATTTATGTTCCCGAAGGAAGAACCAAAGGTGGAACCTATGTCTGATCCTGGATTGAACCTCTCCAATGGCTCAAACGTTTATCAGCAATTCATGAACAGGTTGCCGCTCGGACCTCAGATGTAA

> CaWRKY08-1

ATGGAAAACAATAACAAATCAGAAAGTGATAATGAGATGGAAATTGATCTTAGGTTGAAGCTTGATGCTAGAGAGGAAGAAAATGAAGAAAACAAAATTGGAGAACCATCACAATTGACTGAGAAAACACAAGCAAAAGATCAAGAAATACCAAAAAATGACCAAGAGTTATCGATGCTAGAAAAGGAGATGAAGAGGATGAAAGAGGAGAACAAAGTATTGAGGATGGCAGTGGAGCAAACAATGAAAGATTACTATGATCTACAAGCAAAATTCTCAGCTATTCATCAAAATAATCACAAGGATCACAAAAATTTTCTTTCATTGAGTGGAAATGATGATAGTACTACTAGTGAAGGACTAACTACAAGGGTTCCAAAAATCTTGGATATTATAAATACTACTAATAGAACTTCATCACCAACATCTCATGAAGATGATACTATGGATGGTGATCAATTAGGGTTATCTTTGACTTTGGTTAGTAGTAACAGCACCACATCAAGCAAATTATTGGAAATGCTAGAAGAAGATCAAAGAAAGGAGAAAAAAGAAGACCATCCTACAATTACTCATCAAATTCAAAATAATAAATCACAAAATTTGGGAGGATTAACAAGTCATCATGTCACTACTGCTTCACCACCAAACAGAAAATCTAGGGTTTCCGTCCGGGCAAGATGTGAATCGGCTACAATGAATGATGGCTGCCAATGGAGGAAGTATGGTCAGAAAATTGCTAAAGGAAGTCCTAATTGTCCTAGAGCTTACTACCGTTGCACGGTGGCGCCTGGTTGTCCCGTCAGAAAGCAGGTGCAAAGATGTTTGGAGGACATGTCAATATTGATCACAACATATGAAGGAACACATAATCATCCACTTCCAGTAGGTGCAACAGCAATGGCATCAACAGCATCAGCAGCAGCTTCATTCATGTTAGTGGATTCAAGTATTAGCCCTCTTCTTAACAATCCCAATTCCAGCTTAAACCAACCACTTAATTTCCCTAATTACCATCATAATTTAGCACCTAATTACCATCATAATATTCCAAACTCATCCTCTTCTTCTTCACTAATACCTTATAACCTTTCCATGATAAGAAACAACATCCTTAATTCTAGTGATCCTAATTCACAAGGGAACATAGTACTTGATCTCACCAAGAATATTTCTAATAACCATCAATTCCCCTTTGCAAGTTCTTCTTCAAATTCTCATGAAATGGGCCATTCTAATTGGATGCCTAAATTGCCTAATTATGAAGGGAACAGCCTTTTAGCTGGTCCTAAATTACAAGGTGAACATCATTATAGTCATAATAATAATAATATTCCTCCTACGTTGGCTCATGATGAAAATATGAGTGCAATTGCTGCTGATCCTAAGTTTAGGGTTGCTGTGGCCGCTGCTATTTCTTCACTCATAAATAAAGATCAAAGCCATTCAACTGGAGAGAGTAATGGTGGCTCTATTAATAGGCACACAGATTCTTGA

> CaWRKY08-2

ATGGCTGCAAATAATCCCAGTGCAAACATGCTTGATGGGAGTTTTAGATCATTGGACTCACCTGACAGTGATGATTTCTCAAACCACCTAATTAACTTTGAGCTTTCTGATATTCTCGAAATAGATAATTGGCCCATTCAACAAGATCCGACACTCATACCCCAGTACTCAAATTATGCAGCAAACCAAGTGGTTAACACCAGCAGTTACCAGGAAGAACCTAGCAACAACATTGGAAGCAGCAGCAGCAAGAGGAAAGAAGTAAAGGACAAGGTTGCTTTCAGAATGCTATCACAGATTGAAATACTAGACGATGGCTATAAGTGGAGAAAGTATGGAAAGAAGATGGTGAAAAATAGTCCCAACCCGAGGAATTACTATAGGTGCTCCGTAGAAGGTTGTCCAGTGAAGAAGAGAGTTGAACGAGACAAAGAGGACTCTCGGTATGTGATAACCACCTACGAGGGTGTCCACAACCATCAAGGTCTATCCCCATTCTGA

> CaWRKY08-3

ATGGATACCAATTTGGGAGACAAAACATTTTCTATTGACCTCAACACAAACCCCTCATTGCACAACACCAGTAGAAGTCCGCATGACACGTTGGATGAAAAGTTGGTTAGGATGAGAGAAGAGAACAAAAAACTTGTAACAATGCTAACTACTTTGTGCGAAAACTACAATTCATTGCACTCTCACCTAATTGAGTTGCTGCAGAAATACTTCAGTCATAATGAAGAGGACAATTTCAAATTTTTTTTAAGGAAAAGAAAGGCTGAAGGAGAATGTTGTGAGAATAATTCAGACATCCATTTTGAAGAAGCATCACCAAAGAGGCCAAGAGAAATCACAACCAATGTTTCAACTGTTTGCGTTAAAACCAATCCCTCCGATCAAACCTCGTTGGTGAAGGATGGATATAACTGGAGAAAATATGGTCAAAAAGTGACAAGAGATAATCCTTATCCAAGAGCCTACTACAAGTGTTCATTTGCACCAACATGTCCAGTCAAGAAGAAGGTACAAAGAAGTATTGAAGATCCATCAATTTTAGTAGCTGTATATGAAGGGGAGCACAACCACCCTCACCCATCCCAAGCTGAAATAACAGTGCCATTACTCAACCAAGGTGTTACAACAGATCCAACATTTTTGAACAAATTAATGGAAGAGATTGACACAAATTCACTGCAGCAACATTTAGTCGAACAAATGGCGTCTTCCTTGACTAGCAGCCCTAGTTTCACTGCTGCAGTTGCTGCGGCCATCTCTGGAAAGATTTTCGAATATGATTTACCTTTCAAATAA

> CaWRKY08-4

ATGAAAGAGGAGAACAAGAAGCTAGCAACTATGCTAACAACTTTGGGTGAAAACTACAATTCCTTGAGAACTAATCTAATTGAGTTGCAGCAAAAACATTCCACTCATGAAGAGGACAATAATTCTAAATTATTGTCAAGGAAAAGAAAGGCTGAAGATGTATGTTGTGTAAATAATTCAGACATCAACTTTGAAGAAGCATCACCAAAGAGGCCAAGGGAAATCATCACAACCAGTATTTCAACTGTTAGTGTTAAAACCACTCTCTCTGATCAAACCTCATTGGTAAAAGATGGATATAACTGGAGAAAATATGGTCAAAAGGTGACAAGAGATAACCCTTCTCCAAGAGCCTACTACAAGTGTTCATTTGCACCAACATGCCCAGTCAAGAAGAAGGTACAAAGAAGTGTTAAAGATCCATCAGTTTTAGTAGCTACATATGAAGGGGAGCACAACCACCCCCACCCATCCCAAGCTGAAACAACAGCGCCATTAGTTAACCAAGGTGTTATAACAAATCCAACATTTTTTAACAAATTCATGGAAGACATCAACACAAGTTCACTGCAGAAAGATTTAGTCGCTAAAATGGTGCCTTCCTTGTCGAAGAACCCTAGTTTTGCTGCTACAGTTGCTCAGCCATCTCTGGAATATTTTCTCGAATATGATTTGCAGTTACTCAAATGGGTGTTGAGGGGGAATATAAGCAAAGTCATGCCACCAGGAGTACGTATATATAAACAAAAGACACTTACTGGAGAAACTATTTCAGCTACTCAATCTATGGATGAGACAAACAAAAGTACTCCTACAACTATTACAGATAATACCATACATGCAAGATTGATTAAACTAAGTTATTCGGAGAAGTTTGAAATTAATTTGGATGAACATTACATCAAAGATAGTTGCGAAGATATCCTCAAAAATAGGAGTCGCCAGTGGCGTCATAAATTAAAAAAGATATTTGAAAGTGCACGTTCTAAGGAAGCTGCTCGTAAAATTGAAGTACCAGAACTGACACCCAAAAATTGGAATAAACTTTGTGACATGTGGTCAGATCTGGAGCATAAGAAGCGATGTCAAGCAAAAAAGATCAATCGATCAAAGCTAAAATGTAATCATATAATGGATTCGAAAGCATTTGTAGTTGCTCATGCTGAAATTGGTGAAGAACATGAAGGGTGGAATCAGACAAAATTAACTTCTACAAAAGCACTCATTACTCGACTGAAAAAGGCTGGTCATCTGAACAAGTTGAAGCTAACTATAGAGTCAACCATGACTATTGATGAAATTGTGGATGCTGTACTTGGTAAAAAGTCACGATACATAAAAGGTCTTGATTATGGTTCGAAACCTGACATTACTAGAGCAACACAAAGAAGAGCGGCAGAGTTAGAAGACTCCATTAAAAAGGTGAAGGAGAAAGCTGCTACTGTCCAACATGATCCACAGAAACATTAG

> CaWRKY08-5

ATGGAGGAAGATTGGGATCTACATGCAGTGGTCAGAGGCTGCACAGCTAGTTCCACCACCACAACCACCAGCACCACCACCACTGCAACTTCATGTTGTAGCTTCCAACCAAGACAAGATGGCAACTTTTTCAGCTTTCAAGATCCATTTGTGCCAAGATTTGACAACCCCACAAGTGATTTTGAAGAGTTGCATAATCTTTACAAGCCTTTCTTTCCTAAATCACAAGTACAACCACAACAACAAGTACCTCGTTCTCCTCAAAATAATATAATACCCATTTCACCCCTCTCTGTTCTTGGTGGACTACAAGATCTATCAGCACCTCAACCAACACTAAAACAACAGCAGCAGCAGCAGCAACAACAGCAACAACATATTCATCAGTTTTTTAACAGTACAAGACTAACACAACCCAAACAATCTCTGTCTGTAAATGGTTCAACAAATAGTACAATTACTGCTTCTTCTTCACTTGGTGTCTCACATACTCAAAGCCCAAGACCTAAAAGAAGGAAAAATCAATTAAAGAAGGTATGCCAAGTACCTGCTGAAGGTTTATCTTCTGACATGTGGTCTTGGAGAAAATATGGACAAAAACCCATCAAAGGCTCTCCATATCCAAGGGGATATTACAGGTGTAGCACGTCAAAGGGTTGTTTAGCCCGAAAACAAGTGGAGCGAAATAGATCCGACCCGAATATGTTCATTGTCACCTATACAGCTGAGCACAACCACCCCATGCCAACTCACCGGAATTCCCTCGCCGGAAGTACCCGTCAGAAGCCGGCGAATTCCGAAGCTGGCACCACTGCAAGCGACTCTAACAAACCTACCAGCTCATCGCCGGTATCTTCGCCGGCGTGCCATTCTACGGCGACGGAGAAGCAAGAAAGCAGCAGGGAAGAAAAAGAAGACATTTTCGAAGACGAGGACGAAGAATTTGGCAGTTCCAATATGGGGTTAGATAACATGGAGCCTGCAGATGATGATTTCTTTGAAGGGTTAGATGAACTCGCTGCTCAGGCCACCGGGGATTGCTTTTCCGATAACTTTCAGGGGTCTATGCAGCTGCCATGGCTGTCAAATAATGCCACAACCACCGCAGCTGGCGGTGTTTGA

> CaWRKY08-6

ATGGAGAAAGTTAAAGGATTGGAGAAAAAGAAATTGATTAGTGAGCTAACACAAGGGAAGGAGTTCGTAAAGCAACTGAAAAAACAGATTGGTCCATTGGCTTCACCTGAAGAATGTGATTTACTACTTGGGAAAATATTGTCATCACTAGAGAAATCATTGTCAATTCTTAATTTGAAAGCACTTCTTCTTGAAGGTGGAATTAATGCTAATAATTCAACATCTTCATGTTCATCAATTTCATTTCTTGGTAATAATAATAGTCCCATGAGTGAAGTTTTTGATTCTCCAAGTCATCATTTGGACAAAAATATGGTCTCCAAGAAGAGAAAGAAATCACAAGAGACTAATCAAATAACCATTTCTGGGACGGGGCTTGAAGGTTCACATGAAGATGGATTTAGTTGGAGAAAATATGGCCAGAAAGATATTTTAGGGGCTAATCATCCAAGGGCTTATTATCGGTGCACACACAGGCATACACAGGGGTGTTTGGCAACAAAACAAGTCCAAAGATCAGATGGAAACTCAACAATCTTTGAGGTAACATACAAAGGAAGGCACAGTTGCAAAGTTGCACAATCAGATATCTTTTCACTTAATAATCAAAAACGCCAGAAACACAACAAAAAACAAGAACAAGAGATGGTAATATTCAACTCTACACCAAACCATGACGCAGAAAACTTCAACATCACAACAAAAGAAGAAGTTTTCACACCATTTTCATTTCCACCTACACCCCTAAACCTAGAGAATATTGAAGAGACAAAATTCTTTTGTGATTCCATGGTGCCATTGTTAACGTCCCAAAATCAAGAATTTGGAATGGACTACATGACTCTGCACTGCTCAAATTCAGATCTCACTGAATTGATCTCGACCCCGACCCCAACCTCGATTTCAAATTCCTCATTTGTCGGAGATTGGGATTTGTCTGAGGATTTTGAACCTAATGTCATATTTGACATTGAAGAGTTCTTTAGTTAA

> CaWRKY09-1

ATGCTAATAATGGAGGAAGAAGGACTTATCAAGAATTCATGGTCTTATGAAGATGAGTTGATAAAAGAGCTTCTTGATGATGAATCACCATTCTTGCTAGCTCCTCATGAAGAATATTATTCAACTTCTTCAAGTGAGACTAGTTATTCACTTGATGTAACAAAGAGCTCTATTTCTTCTCTTTCCAAAGGGTCATTTATTGATGATATAGAGAGTGATTTGTCCATGACAAGAAATGGTGTTCAATCTCATGATGTTTCACATGATGCTAGGAATATAGGGTTGGAAAGAGGGTTGAACTTGATGATGAATAAGCAAGAGGCTCTTGAGAATAAATATACTTTGAGAATAAAGACTTGTGGTAATGCAATGGCTGATGATGGTTATAAGTGGAGAAAATATGGCCAAAAATCCATCAAGAATAGCCCATATCCCAGGAGTTACTACAAATGCACTAATCCAAGGTGTGGAGCCAAAAAACAAGTTGAGAGGTCCAGCAATGAGCCAGACACTTTCATAATCACTTATGAAGGACTTCATCTACATTTTGCTTACCCATTTATCACTCTCAATCCACCTCAATTTCTTGACCAGCCCACTAAAAAGCCCAAATTAACAAACCCCAAAGCCCAAAACAATGAAGAAAATGCAAGTGAAATTGATGAAAGCCCAAAATTTGTCAATCCAAGCCCAATTGTGGACCTTGAAGATGGATTGGGTTTTGGTGAAATGGGCTCACAAGGGTTACTTGAAGATATGGTGCCATTAATGATTAGAAACCCATTTATTAAGCCCACAAATTCCTATTCTTCATCTTGCTCTTATTCATCCCCACCAACTTCTCCTTCATTTTCTTGGTCAAATAATTAG

> CaWRKY09-2

ATGGCTTCTTCAGGTGGAAATACGAACACTTTTATGAATTCTTTCAACAGCAACTATTCATTTTCATCTTCCCAATTCATGACTTCTTCTTTTAGTGACCTTCTTTCTGATAATAATGATGATAATAACAACAACAGGAACTGGGGATTTAGTTATCAGAGAATTATGAATTCAATTAACAAAGATGAGGTTCCAAAGTTCAAGTCTTTTCCACCTTCTTCTTTGCCTATGATCTCTTCTTCATCACCAGCTTCTCCTTCTTCTTATCTTGCTTTTCCTCATTCTTTAAGTCCATCGGTTCTTTTGGACTCACCAGTTTTGTTTAACAATTCCAATACTCTTCCATCACCAACAACAGGGAGTTTTGGTAGTTTGAATTCCAAGGAGGATAATTCAAGGACTTCTGATTTCTCTTTCCATAGTAGGCCTGCTACTTCATCATCAATATTTCACTCTTCTGCTCCAAGAAACTCATTGGACGACTTAATAACAAGGCAACAACAGACTACTGAATTCTCCACAGCAAAAATTGGGGTGAAATCAGAAGTAGCTCCAATTCAAAGTTTCTCCCAAGAGAACATGCAGAATAATCCTGCCCCAATGCATTACTGTCAACCATCTCAATATGTTAGAGAACAGAAGGCAGAAGATGGTTATAATTGGAGGAAATATGGGCAAAAGCAAGTGAAAGGAAGTGAGAATCCGCGAAGCTATTACAAGTGTACGTTTCCTAATTGTCCTACAAAGAAGAAGGTTGAAAGGAACTTGGATGGACACGTTACTGAGATAGTCTATAAGGGGAGCCATAATCATCCAAAGCCTCAATCCACCAGAAGATCATCCGCACAATCGATTCAGAACCTTGCTTACTCCAACTTGGATATAACAAATCAGCCAAATGCTTTTCTTGAAAATGCTCAAAGGGATTCCTTGGCCGTAACAGACAATTCTTCAGCTTCTTTTGGAGATGAGGATGTTGATCAAGGGTCTCCTATCAGTAAGTCAGGAGAAAATGATGAAAATGAACCTGAGGCAAAGAGATGGAAGGGAGACAATGAAAACGAGGTCATATCATCTGCAAGTAGAACAGTACGTGAACCTAGAATCGTAGTACAAACCACAAGTGACATTGATATTCTTGATGATGGTTATAGATGGAGAAAATATGGACAAAAAGTTGTCAAAGGCAATCCAAACCCAAGAAGTTACTACAAATGCACATTTATTGGCTGTCCAGTTAGGAAGCATGTAGAGCGAGCATCGCATGATCTAAGAGCAGTGATCACAACTTATGAAGGAAAACATAACCATGATGTTCCTGCAGCACGTGGTAGCGGTAGTTACTCCATGAATAAACCTCCATCTGGAAGCAACAATAACATGCCAGTAGTTCCAAGGCCTTCACTGTTGGCTAACAATTCTAATCAAGGAATGAATGTTAGTAACACTTTATTTAACACAGCACAGGTTGAACCACCAATCACCTTGCAGATGCTACAAAGCTCTGGAAGTTCAAGTTATTCAGGATTTGGAACCTCATCAGGATCTTATATGAATCAAATGCAGCCCACGAACAATTCCAAGCTGATAAGCAAAGAAGAACCTAAAGATGATTTATTCTTCAGCTCTTTCCTTAACTGA

> CaWRKY09-3

ATGTCTGATAACCCTTTTTATCATGATTACATGGGAACTAGTGGAGGGATCAATACATTTCCTTTTTTTGGTGAAAATCCCTCAAATTATCATGACCAACCAATTATTCCAAATATTCAAAATCCAAATCATGAGCATCAGTTCGTACCTTCTTCTTATATGACTCTCACTGAGTGTTTACATGGCTCTATGGACTACAATACTCTATCAAGTGTTTTTGGCATGTCTTGCTCATCATCATCCGAAGTTGTTTGTCCACATATCGATAATCAAGGCTCTACTAGAAAAAGTAGCGTCTCTGCTACTGCTGAACCCATCTTAGATTCGCCTATGGGAGATCAAACGAGCGTTGAAGTCCCACCAACGCCAAATTCTTCGATATCTTCTACTTCTAATGAGGCTGGAGGGCAAGAAGATTCTTCCAAAATCAAGAAACACATGCAGAATAAAGATGGTCAAGAAGGAAGAGATGACAAATCAAAGAAAGAGTGCAAAGCAACAAAGAAAGGAGAAAAAAAGGTAAAAGAACCAAGATTTGCCTTCATGACAAAAAGTGAGATTGACAATCTTGAAGATGGTTATAGATGGAGAAAATATGGACAAAAAGCAGTGAAGAACAGCCCTTTCCCCAGGAACTATTACAGATGCACAACTCAAAAGTGCAGTGTGAAGAAACGTGTGGAAAGGTCATATGAAGATGCATCAATTGTGATAACTACATATGAAGGCCAGCACAATCATCATTGTCCAGCAGCCCTTAGGGGAAATGCATCCTTCTTATCTTCACCACATTTTATGCCTAGTTTTCCTCCACAACTATTTTCCCAAATGCTAATTCCACCAACAAGCAACCAAAATCTCCTCATTACTTCTGAAGCCTATAATAATATTAATAATAACAATTATCATCAACAAAACCAAGGTTCAGAATACAACCTATTTGGTGGAGGCACAAATGATGCATCATGGATCCAGAAACAAGAGCCATCCTAG

> CaWRKY09-4

ATGATTTTGTATGCCCATACACCTGTACTTTATTCCGCTGCCAAAAGTGTGTATAATTTGCTACTCTTGATTCAAAGACTGTCTTTGCACAGGATAATGGGTACCCCTAAAGAAGAGACAACAGATGAAGTTTTCTCCGAGAATTTAGAGCAAAAGCCGGAGCCTGATCCTGCAACCAAATCAGAGTTAAAAGAGAAACGGAGCTTTGAATCGACTTCAGCTGATGTTGTTTCTGGTGAGCTGCAGAAGAGATTGAGTCCTGATGCTGACAAACAAGCATCAAAGAATAATGAAGAGGAAAGTACATATCCTCCAACAGGCCAAGAAGTGTCAAGGATTAGTCAATGTGATAAAGGGATTAATGTGTCACAATCCAATCAAGAAGATATTTCTCTTTCTAGAGTACTAGAGAACCCGTCCGAAAATGTGGGGCAGCTGCAGGTTCTTAAGAGTGAAGCCGGTGCATCTGGATCTAGTCAACTTTCTAGTTTACCGAAGGACTCAGATGCAAAATCATGTGGATCAGAATCTGGTGTAAAACGTTTATCTGGTAAGGCTTCAGATTCTTCAGATCAAATGCAAAGTTCGAACACGGAGATTTTATTATCACAATCTGATCAACAACGAGTAAATTATCCCATACAAAAGCGTGAGAAAGCCCTAGATAAGTTGCAACCAAGGCGGAACCCTGACACTAGTGTCCATGGGTTGACGTCTGATCAAGGAGTGACTCTCCTCAGGGTGCCTGAAAAACCATCCGAAGATGGATATAACTGGCGAAAGTATGGTCAGAAGCTTGTCAGAGGAAATGAGTATACTCGGAGTTATTACAAGTGCACATACCCTAATTGTCAAGCAAAAAAGCAAGTGGAGAGATCACATGACGGGCATATTACAGATATCCACTATATTGGGAAGCATGAACATCCGGAAACTCCAAGTGGTCCTCAGATGCTCCCCGAGTTGGTACTCCCTTTGCAAATGAAACAACCAGAGATTCCAATAATCTCTACATTAGAAGCTGAAGGCGAGAAATCTACTAGGCCCCAAGAAACATGTGAACCTAGTAAGCCATCAGAAGCTCCGCTTGTATTGGACATTGTATCAGCTTGTGGCGGTGTGAAGGGTACGCCTTTAAAGCGACATAAATCAGAAACTGAGGTCGATAAGGATGATGGATCAGACTCAAAAAGACAAAAAAAGGATATAGTAGCTACGGTTGATACTCCACCTATTAAGTCCCAAAGTGAACCACGACACATTGTTCAGACCGTGAGCGAAGTAGATATAATCAATGATGGTCAGCGCTGGCGCAAATATGGGCAAAAAATTGTAAAAGGCAATCCAAATCCGAGGAGTTACTACAGATGCTCAGTTGCTGGTTGCCCCGTGAAGAAGCATGTGGAGAGGGCATCCCATGATCCAAAAGTGGTCATTACAACATATGAAGGGCAGCATGTCCATAATTTCCCAACTCCTAGGGATATAAGCCAAATCTCACCAGTGCCTGATGTCGTTACAACAGCCATACGTACAGATTCCAGAATTGAATCAGGTCACAAACATGTCGTAGAGTCCAAATCTGAATCAGGTGAAAGAAAACATGTTGGAGATTCCAGAACTGAATTGGGTGAAAACAAACATATTGAAAAGTCCAAACCTGAATTGGGTGGAAACAAACATGTTGGAGACTCCAAATCTGAATCAGGTGAAAGCAGACATATCGGTAAGTCCAAAATTGAATCAGGTGAAAACAAACATGTTGGAGGGTCCAGAATTGAATCGGGTGGAAACAAACATGTTGAAGAGTCTAAGCTTGAATTGGGTGGAAACAAACATGTCGGAGAGTCCATACCTGAATCTGCTGAAAACAAACATGTTGGTCTTGACATGGCTGTTCATATTGGTGCAAATTGA

> CaWRKY10-1

ATGGGCGGGTTCGATGATCATGTTGCTATCTTCGGAGACTGGATAACACCTAGCCCGAGTCCAAGAGCCTTCGTGTCTTCACTGCTAGTCGATGATGTTGGAGGATGGTTACCTCTTATGGAGCACACTAATGAAAGTAACTGCAGAAACTTCAATGCCGAACCTCAACAGAATGTCACCGCCTTGTGCAGCACTGATGGAAAGGATGGGGCACGGGCTGGTGCTTCGACTGATCAAACAGTCAAGTCGAGTGCACCATCAGAGCAGAAACCGAGTACTCGTGGAGGAGGGCTCATGGAAAGAATGGTAGCTAGATCTGGATTTCATGCTCCAAGGCTGAATACCGATGGCCTTAGACCTCCTGTTCTTTCACAGAATCAAGAAGCTAGGTCTCCTTATTTGACTATTCCTCCGGGTCTCAGTCCATCAGTCCTATTATATTCACCTGTTTTGATCTATAATCCACTGGTTTGTCCATCTCCTACAACTGGACTATTACCATTAGCATCGGGCGATGAGAGTAAAAGTCTTATGTTGACGGCTGGAATTGCAGATAAGAGGAAAGAGACTGCTTTTGGCAGCAATACTTCATCTTCCTTCTCTTCCAATCCAGTAAATCCGTCCAGTGATCTTTCTCAGCAACTCTTACCTCAAATTGAGGTTTCAGCTCATCCAAATAACTCTCTTCAACCTCAAAGTATGGAAGTAACTCAAAGCGAACAGATACGTCATGGAATATCTAAGTTCCCTATGTTGTCTACTGAAGAGGATTTCCGGGGTAGTCATATCAAGCCAGAGGTAAGGCCCTTTAATATAGTTGGTGGTAGTATGCAACATTCTCAGACTCTTGATGAGCAGAAAGATGAGGACACTAAGCAAAGAGGAGGTGGAGACTCAAAAGATGTCAATCCTCCTGCCGAAGATGGTTATAACTGGAGAAAGTATGGACAAAACCAAGCTAATGGAAGAACGTATCCTCGGAGTTATTATAAGTGCGCGTATCCAAAGTGTCCTGTGAAGAAGAGAGTAGGGGGATATCATGACTGTCAAGTCATGGAAATTATATACAAGGGGATTCACAATCACCCTAAGCCACTCTCAAACCCGATATCAGCCCTCGGATCTTTAAACTCATTTGGTGACGTGCAACTAGACAATGTGGATCCAAGTGGAACAGGTTTTAACAGTGAGCTGGCTTTGGCAACTAGCCAACAAGGACCTACTGCTAAAGGCCTCATGTGGAGCAACAACAAACTTGAAGCAACATCATTAGCAGCTTTGCACTCTGAGTACTGCAGTGGATCTACCACTTTACAATCAAAGGGTGCTCAGCAGGGATCAGCAGATGCAGTTGAAGTATCATCAGTGTTTTCAAATGATGAAGATGATCATGGTACCCGTGGCAGTGTATTACTAGGCTATGATGGTGCAGAAGATGAGTTCGAGCCCAAAAGAAGGAAGTCTACTGTGTCGGATACAAGTGGCACCATCAGAGCAATCAGGGAGCCAAGAGTTGTGGTGCATACTATCAGTGAGGTAGACATCATTGATGATGGATATCGCTGGCGCAAGTACGGGCAAAAGGTGGTTAAAGGCAATCCAAATCCAAGGAGTTACTACAAGTGCACAAGTTCTGGCTGCAATGTCAGGAAGCACATCCAGAGGTCCCCATATGATCAGAAGTCTGTTATCACCACTTATGATGGGAAGCACTACCATGAAGTTCCTCCAGCACGCACCAGCAGCCAAGGTAGCTCGGGAGCGTCAAAGTCTCCTTCCAACCCGATAACTACTGATGCTCAAAGTCATGTAGGTAGGCCTGAGCCCACACAAGTTCAGAACACCAAGCCCGCACAAGTTCAGAACACCAAGTCCACACAAGTTCAGAACACCAACGAGCGTTATGGAAGAGCTCCACAAGTCCAGAACACCAACGAGCGTTATGGAAGAGCTCCACAAGTTCAGAACACCAACGAGCGTTATGGAAGAGTTCCCTCACTTGGATCAGCTGGTCCTATCTCAGGATTTGATAGTTTCGGAACTAACGAGCAGCAAGATCTATCCAGTCTTAATCAGCACCAGTTTTCAGTTCCTCTCAATCCATACATAGGATGGCAACGACCTGTGAATGATGCTGGTTTCGTGCTTCCAGAAGGAGAAGCAATGCCAGATCCTAATTTGAACTACTCCAATGGTTCATCAACTTATCAGAAAATTATGAATGGATTGCCTCCTCAGATGTAA

> CaWRKY10-2

ATGGAAGAAGATTGGGATCTACATGCCGTGGTCAGAAGTTGCACCCCCGCTAACACCGCCAGCACCACAACGAATGATTGTGATCTAGTTCATGGTATATTAAAAGTAGACGGTGCTCTGATGAAATCTTTACGAATAGAAAAAGATTATCGTCAATTTGATAATGATCAAATGACATTGCAGAGGGGCTCTACCACCACTTCTTGCAATCCCACAAACAATATTCATTATTTATGCAGCTTCCAACCAAGACCAAATGATAACAACAACAACTCTTTATTTTGCTTTAAAGATCTGCTAGAGCAAAGAATATTGACCACAAATAGTGCTACTGATTTCGAAGAGTCGCATGAATTGTGCAAGCCCTTCTTTACTGCATCAGAAAGTTTAACCATTTCATCACCACGGAGGGGATTACCTATATCACCTATCTCTGTTCTTGGACGACTACAAGATCTACCACCATCGTCGCAGCAGCAGCAACAACAACATCTTCATCAGCTTACTAATACAAAACCAATTCAGCCTAAGAGACCTCTGTCGTCTCTAAATGGTTCAATAACTAATTGTACTTTACATGCTCAAAGTTCAAGAACTAAAAGAAGGAAGAACCAATTGAAGAAGGTATGCCAAGTAGCTGCTGATGCTTTATCTTCTGATATGTGGTCTTGGAGAAAATATGGGCAAAAACCCATTAAAGGTTCCCCATACCCAAGGGGTTATTACAAATGTAGCACTTCAAAGGCTTGTTTGGCCCGCAAACAAGTGGAGCGAAATAGATCCGACCCGAATATGTTCATTGTCACTTATACAGCTGAGCACAATCATCCTATGCCTACGCACAGAAATTCCTTAGCCGGAATCAGCCGCCACAAAACGGCGAATCCCAACAAACCCACCAGCTTATCGCCGGCGACCAACTCTCCGGCACCGGAAAATCAAGAAAGCAGCAGGGATGACAAAGAGGATATTTTTGAAGATGACGACGATGAATTTGGTAAAACTGAACCGGACGATGATTTCTTCGATGGTTTGGATGAGCTTGTAATCCAGGCTACCGGAGATAGCTTGCCGGAGAAATTTTCGGGGACTTTGCAGTTCCCTTGGTTGGTGAATAATGCCGCTACCACGGCGGCCGGCGGTGGTTGA

> CaWRKY10-3

ATGGAAATCAATTCATCAGTAGTTGATATGAAAAGGTTGATAAAAGAGTTGAATTGTGGTCAGAAATTCACGAATGAGCTAAGAGAGTTGATGAAGAAACATAATATTATGTTGGCTGAGGATTTGTTGGGGAAAATTATGACTTCTTTCTCTAAGTCACTCTCACTGTTGTACTCCAGTGCTGAGTTACATCAATTTTCTCAAGTTCCGATGGTGGCCTTCTCGTATTGTTCAGAAGAATCAACTGATAGCTGCAAGCCTTCATCACTAAAAGATCAAGAAAGATACAACAAGAGAAGAAAAACTTCAGCTACAACCATAAAAGAAGCCTCGACTTTAGTAGATGATGGCCATGTTTGGAGAAAATATGGTCAGAAAGAAATACTCAATTTCCCACATCCAAGGAACTATTATAGATGTACCCATAAATTTGATCAAGGATGCGAAGCAACTAAACAAGTGCAAAGAATCCAAGAAAATCCACCAAAGTTTCGTACCACATACCAAGGTCATCACTCGTGCACAACTTATCCTTCGATTTCTCAAATACTCTTTGATTCTTCAACAAATGAGGATTGTTCGGTGTTACTAAGTTTCAACACCAATAAAATTAATTATCACCAACCATATATTCACTCATTTCATTCAACAAAACAAGAAACTAAAGGGGAGATCTCTTCAAATTGTTTTTCCCCTAATGTTGGCCAAAGTCAATCCGATGATCATCTGATTAGAGCAGCATTGTCACCAGGGTCGTCCGATCATGATGTCAACTACTCTTCTTGTACTACTAATTGTAGTTTGGAGATGGAGATGAAGATAGATATGATGGTGGACTCTGTTGATTTTGAGGATTTAATTCCTTTTGATTTTTGA

> CaWRKY10-4

ATGGAGTCGTTTTTATTACAAAATACAATATCTGATCTGGAGAAGGTAATGGAAGAGTTGAATCGCGGCAAGAAATTTACGCGCCGGCTAAGAGAGATAATAAAGAAACCTAAGATAAATGTGGGTAACGAAGATGCGTATATGTCTAGCGCTGAGGATTTGGTGGGGAAAATAATGAATTCATTTTGTGCGTCTCTATCGATATTAAGCTCTGAAGAATCTACTGAAGAAGTTTCTCAAAAGTCGATGGAAGACTCAAGTGGTAGTTGCAAGACTTCGTCACTTAAAGATCGACGAGGATGCTACAAGAGAAGGAGAACTTTAGAAACAAGCATAAAAGAAACCTCAACTTTGGTGGATGATGGCCATGGTTGGAGAAAATATGGTCAAAAACAGATCCTCAATGCCAAATTTCCAAGGAACTATTTTAGATGCACTCATAAATTTGATCAAGGATGCCAAGCAAGCAAACAGGTGCAAAGAATTCAAGAAAATCCACCACTGTTTCGTATAACATATTATGGTCATCACACTTGCAAAACTTTTCCTAAAGTTTCTCAAATGATATTTGATTCTCCAAATGATCATGAAGATTCTAATTCAGTCCTACTTAACTTTAATTCTGGCAATAATCATCACCAGTTTTTGGATATGACGGTGGAAACTCTTGATTTTGTGGATTTGTCTTTTGAATTTTGA

> CaWRKY10-5

ATGGAAGTCAATGAAGCAGTAAAAATACCTATAGCTAGACCAGTCGCTTCAAGGCCAAGATGTCCTGTTTACAAATCTTTCTCAGAGCTCCTGACTGGTACAGTAGATATATCATCGACAAATGTTCATTCTGAAATGGCTGTTACCGCCATAAGACCAAAGACTATCAGGTTGAAGCCTGCAACAAACCATGCTTTAGTTGGAGAGCCTTCTTCACAGGTTGGCGTGTCCAAGGCACCAGTTGGTTTTGGCTCTGATAACATCTTGCAATCGGTAGAGAAGCCCAAGGTCCTGTATAAGCCTATAGGTAAACTTGCACAAAGGAAAACAATTCCTCTACTTGAAAATAAGGGAAGCTCCGTATCTGATCAGCAACGAGTAATAGCTGACTCTGAGGCTCATGTTCAATCAGCAAATGAAGTTAAGCAACAACATGACCCTACGACAGAATCTAAACAAAGTCTCTCAGAAAAATCAGGACAGGACAAAAAAAAAGTGCGTTCAACAATTGTATCTGGGAGCACAGAGGAGGTTGCACAATCTTTGATCAACACAAGTAATGTTGATCGTCCTAGTTATGATGGATATAATTGGAGAAAATACGGACAAAAGAAAGTTAAAGGAAGCGAATACCCAAGAAGTTACTACAAGTGCACACATCTGAAGTGTCCTGTGAAAAAGAAGGTTGAAAGATCATATGACGGCCAGATAACTGAAATTGTTTACAGGGGTGAGCACAACCACCCAAAGCCTCAGCCTCCAAAGCGCAACTTGTCAGATGGCCATAGGCAAACAGCCATATGCAATGACACTTCTAAAGAAACAAATAACCCTGCATGGGGTAACCAACATCCTCAGATGAGTGAAGCTTACGTCTGTAGGATAGAAAATCAGAATGATCGCGGGTTAACTATACATTCCAGCAAAGTACCATGCTTTTATGATCCCATTGTAGCTGCAGGAATGCACACTGCAGTCAGAAATTCTGAAGATTCTGCTGTAGGAAGTAAAAAATTGAAGGCTACTTGTGATGAACAAAAAAGTAAAAGAAGGAAAATTAAAGGTCCATCCAGTGGAGCAGGTACATCAGGGGAAAGTACATTTCCTTATATACCAAACCAAAGTACTACTGACTCGGAAATTACCGAGGACGGTTTTCGCTGGAGAAAATATGGCCATAAGGTTGTGAAGGGAAGTTCATATCCCAGGAGCTATTACAGATGCACAAGTCCTAAATGCAGTGTGCGGAAGTTTGTTGAAAGAACCACGGATGATCCCAGAGCCTTTATTACTACATACGAGGGAAAACACAACCATGGTGTTCCAAACAGAAGACCAAATTCAGAGGCATCCAAAACAAGCTCAAAATCTTCAGCTATGAAAGAGAAATTATAG

> CaWRKY10-6

ATGGAAGTCAATGAAGCAGCGAAATTACCTATAGCTAGACCAGTCGCTTCAAGGCCAAGATGTCCTCTTTACAAATCTTTCTCAGAGCTCCTGACTGGTGCAGTAGATATATCATCGACAAATGTTCATTCTGAAATGGCTATTACAACCATAAGACCAAAGACTATCAGGTTGAAGCCTGCAACAAACCATGCTTTAGTTGGAGAGCCTTCTTCACAGGTTGGTGTGTCCGAGGCACCAGTTGGTTTTGGCGCTGATAACATCTTGCAATCGGTAAAGAAACCCAAGGTCCTGTATAAGCCTATAGCTAAACTTGCACCAAAGAAAACAATTCCTCTACTTGAAAATAAGGGAAGCTCCGTATCTGATCAGCAACGAGTAATAGCTGATGCTGAGGCTCATATTCAATCAGCAAATGAAGTTAAACAACAACATGACCCTACGACAGAATCTAAACAAAGTCTCTCGGCAAAATCAGGACAGGACAAAAAAAAAGTGCGCTCAACAATTGTATCAGGGAGCACAGAGGAGGTTGCACAATCTTTGATCAACACAAGTAATGTCGATCGTCCTAGTTATGATGGATATAATTGGAGAAAATATGGACAAAAGCAAGTTAAAGGAAGCGAATACCCAAGAAGTTACTACAAGTGCACACATCTGAAGTGTCTTGTGAAAAAGAAGGTTGAAAGATCATATGATGGCCAGATAACTGAAATTGTTTACAGGGGTGAGCACAACCACCCAAAGCCTCAGCCTCCAAAGCGCAACTTGTCAGATGGCCATAGGCGAACAGCCATATGCAATGACACTTCTAAAGAAACAAATAACCCTGCATGGAGTAACCAACATCCTCAGATGAGTGAAGCTTACGTCTGTAGGAGAGAAAATCAGAATGATGGCGGGTTAACTATACATTCCAGCAAAGTACCATGCTTTTATGATCCCATTGTAGCAGCAGGAATGCACACTGCAGTCAGAAATTCTGAAGATTCTGCTGAAGGAAGTAAAAAATTGAAGGCTACTTGTGATGAACAAAAAAGTAAAAGAAGGAAAATTAAATGTCCATCCAGTGGAGCAGGTACATCAGGGGAAAGTACATTTCCTTATATACCAAACCAAAGTACTACTGACTCTGAAATTACCGAGGACGGTTTTCGCTGGAGAAAATATGGCCAGAAGGTTGTGAAGGGAAGTTCATATCCCAGGAGATATTACAGATGCATAAGTCCTAAATGCAATGTGTGGAAGTTTGTTGAAAGAACCACGGATGATCCCAGAGCCTTTATTACTACATACGAGGGAAAACACAACCATGGTGTTCCAAACAGAAGACCAAATTCAGAGGCATCCAAAACAAGCTCAAAATCTTCAGCTATGAAAGAGAAATTATAG

> CaWRKY11-1

ATGGATGAAAAAGATAAGGTTGATGATCAATTACCTATTGAATCGAGCTGGTCACAGCTCAATCCTGATGATGATGCAGATCATGCTTACTTCTTCGAAAATAATATTAATACAAATAACGATACCAGTAGTATACTGAGTGAATTCGGTTGGAATTTTCAACCAGTTGAAGAGAACAGTAGTAGTAGATTTGACAAGATCGATGAGCATTTGGCGGGAAATAGTAGTATTACTACTATGAGTACGTCTCCAGCTTCTGCTACTGCTGCGACTGAACTGACAACGGCGAAAATTAGTACCGATGAACCTGTTTCTTCCAGCTGTTCTGATGATCCGCCGGAAAAATCTACTGCTTCCGGTGGCTCCTCCGCCTCTAAACCGCCGTCAGATACAGTAAGCAAGGTTAAAAAGAAGGGTCAGAAACGAATCAGGCAGCCTCGATTTGCATTTATGACAAAAAGTGAAGTTGATCATCTTGAAGATGGCTATAGATGGAGAAAATATGGCCAAAAAGCTGTTAAAAATAGTCCATTTCCAAGGAGTTACTATCGTTGTACAAATACAAAGTGCACAGTAAAGAAGAGAGTGGAACGATCCTCTGAAGATTCCTCAATTGTAATCACGACATATGAAGGACAGCATTGTCATCATACAGTTGGATTTCCTAGAGGTGGACTTATCAATCATGAAGCTGCATTTACATCTCAATTATCACCTTTACCCTCACAATACTATCATCCCTCCGGTGTTCAGTACCCGCATGAATTAGTTCCTATGACTGCGGCTGCACCAGTAGAATCGCGTACAATGCCAGGTGAAACTGGATCATCAGAAGCTCGTAGATTGCCAGAAACAAGTCAACCTGCTGCAACTGATGAGGGATTGCTTGGAGATATTGTACCTCCTGGGATGCGAAGCAAATAA

> CaWRKY11-2

ATGTCTCATCAGCAAGTGCTTGCTCAACTTACAGCTCAGGCATCCCAGCCTCAGTCGCAAATGCACATTCAGCCTGATTACTCATCTTCTTCAGCAGCAACTGCACTGTCAATGTCACCATTCCAATCCTTAACATCAAATACAGCAGCAAACCAACAGATACCTCCTGCATTGGATCCTAACACAATAAAAGAGTCTTCTGATGTTTCCCTGTCCGACCAGAGGTCTGAACCTGCTTCCTTTGTTGTTGATAAACCTGCTGATGATGGCTACAACTGGCGGAAGTATGGGCAGAAGCAGGTCAAGGGAAGCGAATATCCTCGTAGCTATTACAAGTGCACGCAGCCAAATTGTCCAGTCAAGAAGAAGGTTGAGCGCTCCCTAGATGGACAGGTGACTGAGATTATATATAAGGGCCAACACAACCATCAGCCACCTCAAGCTAGTAAGCGTTCAAAAGAAAGTGGAAATCCAAATGGAAACTATAATCTTCAGGGGACCTATGAGCCCAAGGAGGGCGAACCTTCTTATTCCTTAAGAATGAAGGATCAAGAATCTAGCCTAGCAAATGACCAAATCTCCGGCTCAAGTGACAGTGAGGAAGTAGGTAATGCAGAGACCAGAGTGGATGGGAGGGACATTGACGAACGAGAATCAAAGCGGAGGGCGGTAGAAGTACAAACTTCTGAGGCCGTTTGTTCTCACCGGACTGTTGCAGAACCTAGGATCATTGTTCAAACAACCAGTGAAGTTGATCTGTTGGACGATGGTTATAGATGGCGTAAGTATGGCCAGAAGGTTGTTAAAGGAAACCCTTATCCAAGAAGCTATTACAAATGTACCAGCCAGGGATGTAACGTAAGAAAGCATGTCGAAAGGGCTGCAAGTGACCCTAAAGCAGTCATAACAACTTATGAAGGAAAACATAATCATGATGTGCCTGCAGCCAGGAATAGTAGCCACAATACAGCCAACAATTCCACGTCACAATTGAGGCCACACAACCCTGTATTCGATAAACCAACTGCAATGCGAAGATCAGACTTTCCAAGCAATGAACAACAACCTATAGCACTTCTACGTTTCAAAGAAGAACAAATTACATGA

> CaWRKY11-3

ATGAATAAGCTAGAGAAGGTTCTTCCACCTGGATTTTGGGATGTGATGGACCATCTTTCTGTGCATCTTGTGCATGAAGCTCGTCTTGGAGATCCGGTTCAATATCGATGGATGTATCCATTTGAGAGGACTATTGGCAAGAGTAAACGGAGTATCAAGCAAGAATATAGAATTGAAGGCTCAATGAGTGAAGCATATCTTGCTAAGAAAACGACCAATTTCTGTTCGTATTACTTTGCAAGTGATGTTCCATGTTCACGAAATAGGCCCAATCGTGTTATCAATTTTCAATCAACCCGGCGAAGCTACTTTGTGCAGTTGTATGACAATGATAAAATGTTTGACTATTTTTTAACATGGTTTAAAGATTATGCTTATGATACAAGAAATGGTCAATTTGATCAATTTTTGAAGGATTTATCTTGGGGACCCATTAAGATGGCAGGCGGCGATAAGGATAAGGGTACTGCTAATGCTCCTAAAAAGAAATTAAAGAAGGCTAGTATAATTAATAGGCGATTGACAGTATCTACTTCACATATTGGGATATCAAGTCTTCCGCTTCGAGGCAACATCTCTGAACCTGAAATTCCTACCATATACCAGTCTATTACACATGGCCAGGTTTATCCACAAGCTGATATGAGGGACATGCGCAACAGACTCATCATAGAGCCTGACGACTATGGGGATGCTGTCACGAGTTGGGGTAAGATGCCAGACAATCTCAGGAACCAGATTCTTTTGGAATTCAGGGAAAAGGAACTTGGGAGGCCAATAACTCAGGCTGAGGCATTCAAGACTACACACACAAGGAAGAAGAAAATTTCTGAAGATCCTGATGTTTGGGTTGAGCCACGAGCTCAACTGACCTATGAGGATCAACTAATAAACTCAACAAAATGTCAGATTGTTGAAGCCAAAGAAGAAAATGAGAGGTTAAAGTGGATGCTATCTAAAACCATGAAGGATTACAAATCCTTGCAAATGCATTTTCAAGGCATAATTACTCAAAATTCCAACAATATTATTGCAAAAAGTCCTTCTAATATTATTGAAAAAGAAGAAGTGGAACATCTTTCTCTAAGCCTAGGGAGATCATCAAGTTGTAGTACTTCAAAAGAGTTCAAGAAGGAATTATATGATCAAAATGATGAGTGTAAAAGTCAAAAAAATTGTAAATTATATGATGGTCTTGATTTGGGATTAGATTGTAAATTTAGTCAAGATCATTTAACAGATGATCATAGCCCAGAAAATAGTTTTGAAGAATCAAACAAGGAGAATATAAAAATGGCCCGAATTTCGAGGAATCATGATGAAGATGAAGATGAAGATGAAGANGCTAGGGTGTCTATAAGAGCTGTGTGTGGCACTACTACGATGAATGATGGATGTCAATGGAGAAAATATGGACAGAAAATAGCTAAAGGGAATCCATGTCCTAGGGCTTACTATCGTTGCACTGTCTCCCCAACATGCCCAGTAAGAAAACAGGCCCATTTTAATCTTCAAGACCGCGAGGAGTATGTCGCATTCGCAAAGGTTCAAGACATTGACCATCGCGTTCGCGATGAAGGGCTGGTCCAACCCAGTCCACGTACCTCCTTGTGTTTGCAACCACTTTGTGTCACGATCGTGGTTTACTGTTCTGGCCTACAAATTGACTTACCTACTGGTGAGTTACAGTCAGAGGCGAATCTAGGATCCAAACTCTTCAGGTTCGGGTGCTCAGTCTCAGCCGCGACCGTGATCTACGAGTACCTTGTCTATATTCCGCGGTTGGCGTTAGCCCAGGCATTCACCATAAATGTTCAGGACAATAACCAGGCTGCAGTTCTATATCAGCAAGGAGAGGATTTAGTTGTATCCAGGATTCAAGATTTCATGAGGATGAACCCACCAGAGTTCTATAGGTAG

> CaWRKY11-4

ATGGAGAATTACCCACCACTATTTCCATCATCATTATCGTCTCATGAGTTTTCATTAATGAATAAGAAGAGGAGTAATACTCATGCAAAAGAGGTTTTATTATTCCAAGGAAAGAACAACGGGTTCTTGGGGCTAATGGCAAGCATGGAAACTCCGAGCGGTGTAACAAATAGTTTTGAGGACGATGTGATGAAATCGTGTAAGAAGAAGGGAGAGAAGAAGATTAAGAAACCAAGGTACGCTTTCCAAACAAGGAGCCAAGTGGATATTCTTGATGACGGTTATAGATGGAGGAAATATGGACAGAAGGCTGTCAAGAACAACAAATTCCCAAGGAGCTACTACCGATGCACGCATCAAGGATGTAACGTGAAGAAACAAGTACAAAGGTTGTCCAAGGATGAAGGAGTAGTGGTAACTACTTATGAAGGCATGCATTCACATCCCATTGACAAGTCTACCGATAACTTTGAGCAGATTTTGAGTCAGATGCAAGTCTATGCTTCCTTCTAA

> CaWRKY12-1

ATGGAAAATTTTTCCTACAATTACTCAAACCCTAACCCTAATAATGGAGATATTTATAGCTCGAATTTTATCGATACACCGGAGAATTTTGAGCTCTCCGATACACCGGAGAATTTTGAGCTCTCCGGTTATTATCTCTTCCCTGAAGATGGATTGAGTGATGAGTTTTTGTCACAAAATGAATTTGTTCAAAGTGCTTCCGATAGTAGAGGATCATATTCCAATATTAAACCCGCTCCAACAACTACTCATCATAACATGCAAGTAAAATGTACAAAAGGTGTAATGAAGAAGGTGGATGCAAAGTCTAGGGTTGCATTTAGATTTAGATCAGAGTTGGAGGTGTTGGATGATGGATATAAATGGAGGAAATATGGCAAGAAGATGGTCAAGAATAGTCCAAATCCAAGGAATTACTACAAATGTTCAAATGGAGGATGCAATGTGAAAAAGAGAGTAGAAAGGGACAATGAAGATTCAAGCTATGTCATTACTACCTATGAAGGAATTCACAACCATGAGAGTCCCCATGTGATTCACTACACACAGTTTCCTCCCAATAATATTGCCCTTCATAACCTTCACCTATAG

> CaWRKY12-2

ATGTTTAGCCAGAGCTTGCTTGAAGATCATCAAGATATGTCATCACAACTTGGATTTTTCTCTTTTCCTCCAAATTACAACAACGTGGGCATGATTAGTACTACTACTACTGCTACTCTACCATTTATTGGATACAACCAAAATACTCTAAAGACCCTCACTATGAATATCCCTCCCTCTTTTGATCATTCCTTAAATATTCAAGAATCTACACATGATCCAAGGCGCAAAGAGGACCTTAGTCCTATATTTGGGGGACCCCATCTTCATTCCTTGCAAAAATCCACTCCAAATACATGGGCATGGGGAGAAGTGAATGAGAGCAGCAATATTATTAAGAGAAGGGAGTTTGATCATGATCATAATTTAGGGGTTTCATCAATCAAGATGAAGAAGATCAAATCATCAAGAAGGAAAGTAAGAGAACCAAGATTTTGTTTCAAGACTATGAGTGATGTGGATGTGTTGGATGATGGTTATAAATGGAGAAAATATGGCCAGAAAGTTGTTAAAAATACCCAACATCCCAGGAGCTATTATCGATGTACACAAGATAATTGTAGAGTTAAGAAACGAGTGGAGAGATTAGCAGAAGATCCGAGAATGGTGATCACAACATATGAAGGGAGACATGTTCACTCTCCATCACATGATGAAGAGGATTCACAGGCTTCATCACAACTTAATAATCTCTTATGGTAG

> CaWRKY12-3

ATGGTTATGTTTTCAAAAATGAATGAGGAATTAGCCCTTCAAGAAGCTGCTTCGACAGGGTTGAAAACAATGGAGCATTTGATCAAGTTGGCGGCTAATGAATCGGTGGTTAAGGTGGATTGTCGTGAGATAACTGATTTTGCTGTTGCAAAGTTGAAGAAGGCTAATGCTATGGTGGGACGAACCGGTCATGCTAGGTTCCGTCGTGGTCCTATTCAGGTTCAGGCTCAGAATGAGGCTGAATCTCAGGCTCAGACGCAGGCTAAAGTTCATGATTCCCTGACTACATTGTCTCTTTCTCCGTATGGTTATATGGAGAAAGAAAGAGTATTGACACCAACACAGTTGTCTGCAGTGGCGCCAGTGCATTCAGGATTGACGCTTGACTTCAGGAAGCCAATTGTTAATACTGGTGTTGGTACTAATGTTGGTGGTGGTGCTGGTGTTATGAAATGGAAGGAGTTTTTTGGTAGCATGGAAAATGCGATTGGCTCTTCTTCCTTCCTTTCGTCTATTATAGGCGAAGGGAGTGGATGTATCGCGGATGTTGCTTCTCTTTCTTCTTCCATGTCGTTGCTCCCGACGGCACAGGTTGTTTCCGCTGGGGAACAACCATCTGCCGCCGAAAAAAGATGTCGTGAGCAAGAACAATCTGGAGATGGTTCTGGCAAAAAGAGGAAAGTTTTTCCTAGAAAGGTGATACGAACTCCAATAATAAGTTCAAAATTTGCTGATATACCAAGTGATGAGTGTTCATGGAGGAAGTATGGTCAGAAGTCGATTAAGGGTTCACCATACCCACGGGCTTATTACAAGTGTACCAATTTTCCTGGATGCCCAGCAAAGAAGCATGTGGAAAGGGCTATGGATGATCCTATGATGTTAATTGTGACCTATGAAGAGGAACATCGTCATACTCAAGCCGCGATGCAAAAGTACAATTCTCAAATGATGGCTTTTGGGTCAATGGAAGGGAAGAAGGAGTGA

> CaWRKY12-4

ATGAATAAGGAATTAGCCCTTCAAGAAGCTGCTTCAACCGGGTTGAAAATAATGGAGCATTTGATCAAGTTGACAACTAATGAATCGGTGGTTCAGGTGGATTATCATGAGATAACTGATTTTGCTGTTGCAAAGTTGAAGAAGGCTAACGCTATGGTGGGACGGACTGGTCATGCTAGGTTCCGTCGTGGTCCTGTTCAGGTTCAGGCTCAAAATAAGGCTGAATCTCAGGCTCAGGTTCAGAATTCACTTACTTCGTTATCTCTTTCTCCGTATGGTTATATGGAGAAAGAGAGAGTACTGGCATCAATGACGCTGTCTGTAGTGGCGTCAGTAGCGGCGCCGGTGCAGACAGAGTTAACACTTGGCTTCAAGATGCCAATTGTTAATGATGATGTTGGTGGTGCTGGTGTTGGTAATATGAAATGGAAAGATGATTTTGGTGGCATGAAGAATTGGATAAACTCTTCTTCCTTCCTTTCGTTTATTACAGGCGAAGGGAGTGAAAGTATCGTGAATGTTGTTCCTCCTCCTTCTTCTTCCATGCTATTGCTTCCAATCGCACAGGTTGTTTCTGCAGAAAAACAGTCATCTGCCACTGGAAAAAGATGTCGTGAGCAAGAACAATCTGGTGATGGTTCTGGCAAAAAAAGGAAAGTTTTTCCTAGGAAAGTAATAAGAACTCCAATGATAAGTTCAAAAATTACTGCTATACCAACAGATGAGTGTTCATGGAGGAAGTGTGGTCAGAAGTTGATTAAGGGTTCACCATACCCATGGGCCTATTACAAGTGTACCAGTTTTCCTGGATGCCCAGCAAAGAAGCATGTGGAGAGGGCTATGGATGATCCTACGATGTTAATTGTGACCTATGAAGAAGAACATTGTCACACGCAAGTCGCGATACAAGAGTACAATTCTCTAATGATGGCTTTTGGGTCAGTAAAAGAGAAGAAGGAGTGA

> CaWRKY12-5

ATGTTTTCCAAAATGAATGAGGAATTAGCCCTTCAAAAGGCTACTGCGACCGAGTTGAAAATAATGGAGCATTTGATCAAGTTGGTAGCTAATGAACCAGCAGTTCAGGTGGATTGTCGTAAGATAACTGATTTTGCTATTGCAAAGTTGAAGAAGGCGAATTCTATGGTGGGACAGACAAGTCATGCTAGGTTCTGTCTTGGTCCAGTTCAGGCTCAGAATGAGGTTAAATCTCAGGCTCAGGCTATGGTTCAGGTTCATGATTCCCTGACTTCATTATCTCTTTCTTTGTATGGTTATATGGAGAAAGAGAGAGTGCTTGCACCAGCGCCGCTGTCTGTAGTGACGCCTGTGGCAGCGTCAGTGCAGACAGGCTTAATGCTTGGGTTCAGGAAGTCAAGAGTTAATGTTGTTGCTATTGCTGGTTCTAGTGGTGGTTCTAGTGTTGGTGATATGAAATGGAAGGATGCTTTTGGTAGCATGAAGAATGGGATAAGTTCTTATTCCTTCCTTTCATCTACTACAGGCGAAGGGCTTCCTTGTTCTTCTTCCATGTTACTGCGTCTGATGGCACATGCTATGTTCGCAGGAAAACAACCATCTGCCATCGGGAAAAGGTGTCGTGAGCAAGAACAATCTACTGATGGTTCTAGCAAAAAGAGGAAAGTTTTTCAAGAATTACTGATAAGAACTCCGGTGATAAGTTCAAAAATTACTGATATACCAACAGATAAGTGTTCATGGAGGAAGTATGTAAAGAAGCATGTGGAGAGGGCTATAGATGATCCTGTGATGTTAATTGTAACATATGAAGAGGAGCATCGCCATACACAAGTCGCGATGCAAGAGTACAATTCTCAAATGGTGGCTTTGGGCTCAATAGAAGAGAAGAAGGAGTGA

> CaWRKY12-6

ATGGTGGGACGGACAGGTCATGCTAGGTTGCGTCGTGCTCCAGTTCAGGCTGTTCAGGCTCAGGCTCAAGCTAAGGTTCAGGTTCATGATTCCCTAACTTCTCCATCTCTTTCTCCGCATGGTCATATGGAGAAAGAAAGAGTGCTTGCACCAGCGCCGCTATTTGTAGTGGCGCATGTGGTGGCGTTAGTACAGACAGGCTTAATGCTTGGGTTCAGGAAGCCAAGATTTAATGTTGTTGTTGCTGTTGGTGCTAGTGTTGGTGATATGAAATGGAAGGATGCTTTTGGTAGCATGAAGAATGGGATAAGTTCTTCCTTCCTTTCGTTTGCGACAGGCGAAGGGAGTAGAAGTATCTCGAGTGTTGCTCTTCCGTCTTCTTCCATGTTGTTGCGTCTGATGGTACAAGCTATTTCCACAGGAAAACAACCATCTGCCACCGGGGAAAGATGTCATGAGCAAGAACAATCTAGTGATGGTTCTGGCAAAAAGAGGAAAGTTTTTCCTAAGAAAGTGATAAGAACTCCGGTGATAAGTTCAAAAATTACTGATATACCAGAAGATGAGTGTTCATGGAGGAAGTATGGTCAGAAGCCGATTAAGGGTTCACCATACCCACGGCTTATTACAAGTTTACCTGTTTTTTCAAATGTCCAGCATAAAAAGCACGTGGAGAAGGCTATGGATGATCCTATGATGTTAATTATGACATATGAAGAGGAGCATCGTCATACACAAGTCGCGATGTAA

>CaWRKY01-1

MDCGFNWESNSLINELTQGLEHAKQLRASFSYDKIQELDFHLQMIFSSFEKSLSILKWNDTVTQSPLLIAPLVSTGAPESSISVDDNNNNNVSSEIPHVGYGEGTVYADVTPKMDDQDFKYVSKKRKQMPTWSEQVRVNAENGYERPTDDGFSWRKYGQKDILGAKYPRSYYRCTYRLMQNCWATKQVQRSDDDPALFEITYKGSHTCNQTFHSATQLKSPEKHKLKKQANNPRTIQSNQMLANFQANLRVNINDLDKKEATSSFQFSPTFSGFVSENLHFQRSQDDDNLVGGYSLSFVSPTTPESSYFSLPSSHMNDSRRIHNVHHSESDLPDLFSANTSSTSSPIIGLEFPLERVELDPNFPFHNSEFFR

>CaWRKY01-2

MQQPNDQMPMQNNYHSHVGLDCDNIDWAGLLSAGPSINNESIATTSNVSINHRNMNTNMMEGGEQHQQQLLPQQQQHEVCRRDKGRIIKKRKYVPPRIAFHTRSTEDILDDGFKWRKYGQKAVKNSTHPRSYYRCTHHTCNVKKQIQRHSKDTSIVVTTYEGIHNHPCEKLMETLSPILKQLQFLSRF

>CaWRKY01-3

MMIMDWGLQAVVIGSSSTSCSDIHGSNYVDFSPNLDFQESEYLSFSHHHHQHHEMKKEVYSDELEQLYKPFYHVGGQNMLMGSSISLVPKEVIKEEKREEEQQQVVAASNTYVPKYKKRKNEQKRVVLQLKADDLSSDKWAWRKYGQKPIKGSPYPRSYYRCSSSKGCLARKQVEQSCTEHGIFIVTYTAEHNHSQPTRRNSLAGTTKSKFPNSKNSINSPKKIVKEEKITSPHCSTSNLGLSPEAARMIDEFPEINQENNIIIGGDNNYYDEDGMRNIFEGSDENECVSIEEMFDGDFFAGLEDIHDGFNSSFGCNNSAFPFSST

>CaWRKY01-4

MFVYELFSSLQKVAHTSFCDFHLPTSNIFQVKCISRSFKFQLQNLSANGAYICSLVIVFCFHTQRYVSMKRGLFSVFSDSSECPDIRKIGQKVVFKVQMEGKAIKQKNEGPPSDCWSWRKYGQKPIKGSPYPRGYYRCSSSKGCSAKKQVERCSKDASLFIITYTSSHNHPGPNLPNNKDTVTHDSTATLPQQDREPTVDKDVPLKDNGDSTTTTTTATSISQGIPEENLFTDSFLGTISYDDFLPLSYPQLMEFPKSELSEENDFYDELGELQLPPSSTSFAGIFEEAILVDPS

>CaWRKY01-5

MESYKEIKIEDHPMYYLDNNFAVTNSHSFTGLISDYYGVEGRNIMNTSSSLGFMELLGFQDLMCSSSASFFELPKEENSCPAVCVSEEVKPTAGESQNKLISTVAAANVFNTPSTPNCSSISSETNEGHTNTTHEDAEAGEVLDHQDQQHTNTKQQLKAKKTVSQKKQREPRFAFMTKSEVDFLEDGYRWRKYGQKAVKNSPFPRNYYRCTSATCNVKKRVERCFSDPSIVVTTYEGKHTHLSPMNTIMPRPSCYPITPVPASPGAFPLPMQFNINQSFNNLTSSLAMNNQLDHAAFVAQGRRFCTSEMLGDEGLLQDLMPSTLIKENYR

>CaWRKY01-6

MERGGAERDHQLNNYNLQVSFSSSSVAANNIHELGFVHFADHNLSFLAPSSQSSQISQPLQAASVSVTPPTTINTNVAAGGSNNVTGGLGFSHNELVINRSSWNSDQVETLDPKAVNDENCGGNANEGNNSWWKTSSSDKGKVKIRRKLREPRFCFQTRSDIDVLDDGYKWRKYGQKVVKNSLHPRSYYRCTHSNCRVKKRVERLSEDCRMVITTYEGRHNHSPCDDSNSSDHDCFTSF

>CaWRKY01-7

MALDLFAIEQTASAGLKSMDHLIQFVSSNPTAKPDCREITEYTVSNFRNVISMLNRPTCHARVRRVGPVQPVKVAPPVVSSPVVAPPMVAAPVEKEKEKEKMFRSTPALTFDFTKRKVAVPAAPSPAAGVGVVSKDVAMANSTNSSSSSFVSTITAEGSVSNGRVFPSMDLPPRPPVTAPAAFSGKPPIAGKRCRDHDVSDEFSGRTSSAGKCPCKKSKPKVKKVIRVPAISSKTSDIPADEFTWRKYGQKPIKGSPYPRGYYRCSSLKGCPARKHVERATDDPRMLIVTYENDHEHHHNIQTAFSGAAIGSRDGSSGQRMMVFESMGQK

>CaWRKY01-8

MDCSFNWQYKTLINELTRGIEHAKQLKAYLSSVASTSENQELLLQKILSSYEQSLVILKRTGSTVHSSKPLPPMCGAIESSVSVDGSPKSDDKKRCFKEHQELLDISKKRKSQLTRTEQVKVSAESGFEGPTDDGYSWRKYGQKHILGAKYPRSYYRCTYRHMQNCWATKQVQRSDDDATVYEITYRGSHNCRQATNRASLEKQELKKQAVYQTGQQYSNQALMNSRANLKVDTDDLEKNETACPFSFPPTFSGLTDENQHFQISDVDDNRTTSQSQAAAR

>CaWRKY01-9

MDCAVNWEYKTLINELTQGIEHTKQLRAHFSSVDSTIQNQELLLQKILSSYEQSLLILKCSVGGSMVQSSSAMMPTCGVIESSVVSVYGSPKSDDKKRSFQDHHEVIDISKKRKLQPTWTEQVKVSPKSGFEGPTDDGYSWRKYGQKDILGAKYPRSYYRCTYRHMQNCWATKQVQRSDDDPTVFDVTYRGSHSCHHATYYVQQSTSPEKREFKKEAVYQNRQNYSTQALMSLRANLRVDTNDLDKNEQAACHFSFPPTFSSGLTDENHRRFQISHVDENLIGSGYSASFVSPTTPESNYFSVSSSSQMNGYGMIHNLNHSESDLTDIFSANTSTTSSPIVGDFSLDNLELDTNFPFNNPNFFS

>CaWRKY1-10

MDCVFTWDYNSLINELTQGMEHTKQLKTYLSSVPSTSESTPNSLLQKILSSYEKSLLILKWTGSTVQSLQPLPPTGGAIEPPPAASDDRSHNCDDKKRSFNDHTELIDSSNKRKSQPRWTKQVNVRTGRGFEGPLDDGYSWRKYGQTKILGAYYPRSYYRCRYWLLKGCAATKQVQRSNDDITIFEITYKNSHTCREVTNSALQPKSPEKQANHQTPTVDGYSPSFASPTATESNYLSVSGSQMNSFGRVHNSNHSESYLSDIGIIQNLYHSDSDRTYTLSANTSTTSSSIEGMDVQWAEFRQVKVG

>CaWRKY02-1

MCSPQKDMTNNYQGDLADIFRGGNSTTSGDQSSTSVVPVPDGWQFPSINYSASVIEEPTASLVQDFGDPFCNLRDPLLFHDLDMIPQASSSLFNPTQENNNHFGPNIDSPSMKIRRPNIFSTMLQISPTTKLAMSPCDIACSSPNSNVNVIGALVPTQDAIISPNSSKTCLVENSGLQISSPRNTGIKRRKSQAKKVVCIPAPAPANSRQGGEVVPSDLWAWRKYGQKPIKGSPYPRGYYRCSSSKGCSARKQVERSRTDPNMLVITYTSEHNHPWPTQRNALAGSTRSQPNNSKHTTTSKNNTNIMPNNSQYQGDTSFNEDEQNERNINHDNNVAQANISTYPKVKEEVAEEDLQQQLGEMRNVEFSKGSYQPILPDSSNQCHEDFFADLVELEADPLNFLFANTLSGDINEVGQKKAIDAFNLYNWSKDRNTNINNKGTQADT

>CaWRKY02-2

MEIEIAADAAITKFKKVNSLLDRFRTGHARFRRAPIDNLKKDYVDPEVYCLTPIQQLPPSAYDLNNNQIFQNPKQELVTKSINFSHAPEIWCANSFNMSTLTGETESEQISNLSQVSSAVKPPLSSSSSFKIRKCTSSENGFSGKCSGSSGRCHCSKRRKLRPKRVVRVPAISMKLSDIPPDDYSWRKYGQKPIKGSPHPRAYYKCSSVRGCPARKHVERALDEPTMLVVTYESEHNHSLSVAETSSLILESS

>CaWRKY02-3

MRKGDDESVKKRAGDDEVSQPNVKRARVSVRTKCDYPTINDGCQWRKYGQKISRGNPCPRSYYRCSVAPLCPVRKQVQRCLEDMSILITTYEGTHNHSLPIEATAMASTTAAAASMLLSGSSTSSQSPKNFTNLANYSKTTPLYLSNSSSNPFPTITLDFTAFPTTSSFTSFNFPSNFQPGSGLLSNSLSFSSPESSTIPKILGSGCLNYDSTSTLPYHKNLINIGSSQKQFDQPFIGKNNTSTSDKLKEDSSQQALTETLTKAITSDPSFQSVLAAAISSMVGATKT

>CaWRKY02-4

MGDELRDLYYHQPFQEDSSSSVVQNIHMLDPSFMSYTNEYLYGSSSDYVNNNSLGKPFGFSSSSPSPFSSSKDDMIKQDLHHVDANININNISETPVTPNSSVSNSSSNEAAGDHDDSNKKDKQVKDESLEDGEDASKKENKGKKKGEKKQRPPKFAFMTKSEVDHLEDGYRWRKYGQKAVKNSPYPRSYYRCTSQKCQVKKRVERSYQDPSVVITTYEGQHNHHLPATLRGSVARMLNPSMLAQPSPLMAPQAAAFHQELIMAQMPQLFGHGNAFGSPPMYRQNLTHPLQNHQQMQLPPHHDYGLLQDMVPSMFNLKQEP

>CaWRKY02-5

MEGRFNNFFVSEQDDSENSPENSSDSPRSAMFNDNKMITSTSSPKRSRRSIEKRVVSVPIKEVEGSKMKGEISMPPSDSWAWRKYGQKPIKGSPYPRGYYRCSSSKGCPARKQVERSRADPNMLIVTYSCEHNHPWPASRSNQHNHRTITPTSCTNNNTKTKTKTIASLTASPATTTIITTSDIPILHFEQQKATTDFAVRPSEPNSDEKFVNLGESSLINAEFGWFSDLVECNSTTILESPILTQVEVNDFDMSSTLTMQEEDVSLFADLGELPECSRVFGRGMMERDEERDRHSLTPWCGTTG

>CaWRKY02-6

MAVDLMMDYRNTSNSSSNNCINFVTKLEEKAVVQEAASGLESVEKLIRMLSRNKSPQIQQQNKSPMEIELVADAAVTKFKKVISLLDRNRTGHARFRRAPLAATTSPSPTNCNKDIVDTKVYSPTPIQQVPLVSYEHYNPLVPPKTISFSYSPEMSRTNSFNISSLTGDTESKQHSSSSAAFQITNLSSQVTNSAGKPPLSSSSLKRKCSLSENAVSGKCSGPSGRCHCSKRRKLRLKRVIRVPAISMKLADIPPDDYSWRKYGQKPIKGSPHPRGYYKCSSVRGCPARKHVERASDDPTMLIVTYEGEHNHSLSVAETSSLILESS

>CaWRKY02-7

MDKGWGLTLESSSSSDKVGFFMNKPVFGFNLSPRLNPAEMFPSSDDKRAIVNEVDFFSEKKPIVKKENSQGDRTDQCVVNTGLQLVIANAGSDQSTVDDGISSELVLEDKRAKIQLAQLQVELQRMNSENQRLKGMLTQVNNSYSALQMHLVTLMQQQQQQQQQQQMISRTESTHAHEVVEAKFNDEKKQEKEGTIVPRQFMELGPSGSKADPLDEPSNSHTSSEERTLSGSPRNNMELLSRDKAIGREESPESESWAPNKVPKLMNSSKPVEQPTEATMRKARVSVRARSEAPMISDGCQWRKYGQKMAKGNPCPRAYYRCTMAVGCPVRKQVQRCAEDRTILITTYEGTHNHPLPPAAMAMASTTSAAANMLLSGSMPSADGLMNTNFLARAMLPCSSNMATISASAPFPTVTLDLTAQNSNAALPNYHQRVNHANNAQFQFPLPAGLNHPNFIASMSAPQMPQVLGQAMYNQSKFSGLQVSQDNIHHPSISHDTLSAATAAITADPNFTAALAAAISSIIGCGSHPNNNGNSTMSGPSSNNNNTSSFPGN

>CaWRKY02-8

MNLALAQSPLFMIPSGFSPSGFLNSPGFLSPLQSPFGMSHQQALAHVTAQAECSSSYMQMQAEDQCSAQVASAEAALGNELLTDPKESSLQIKECLQPRLDKKPSDKQGKQFELTEVPQFENKTSFGAFDKSACDGYNWRKYGQKKVKATECPRSYYKCTHLKCPAKKKVEKSVDGHITEITYNGRHNHAQPTKQRKDGSALDSTDGSGVQPDISTHDWTVMNSSDGSSPSHSEQVPNQMASELVKKECDETKSNLIEVDEGHDEPDAKRTKMAVETLASSHGTVAESKIILQTRSEVDILDDGYRWRKYGQKAVKGTQHPRSYYRCTYAGCNVRKQVERASTDPKAVITTYEGKHNHDIPTVIRNRGTRNTAKDTWR

>CaWRKY02-9

MENNQPMLLLGSASSYYNSMNGGLKTSFTQISRDQMEVDTSENHNKYISSLSVKKKGDNKKIKKPRFAFQTRSQVDILDDGYRWRKYGQKAVKNNNYPRSYYRCTHEGCNVKKQVQRLSKDEGVVVTTYEGMHTHPIDKPNDNFEQILHQMQIFPNHPLN

>CaWRKY03-1

MEDSHSHSHYPRPYSNSAPLSSINETSEQVKFSSSDAALFSSSDAAFVYSSAFGSNSSSSAKYKLMSPAKLPISRSPCITIPPGLSPSSFLESPVLLSNIKAEPSPTTGSFSKFQLMQGSSGSAAFSLMRSCSSGNAYGETTGEFEFEFPIGSSSTSGSLAKEAVICAGFNQQQSEPLIQVQNRCPSQSLAPPALVKSEMPNSKELSLPTPVCLDASLISTAAAATDNEEVNQRGQSNPSSHRSSADNKNVSSVTADRSSEDGYNWRKYGQKLVKGSEFPRSYYKCTYPNCEVKKIFERPPDGQITEIVYKGSHDHPKPQPNHRFTPGALTSVQEDRGEREACLTGQEVIPLSGLSFLEDKFNTNAQTSNTEPSGTPLSPQQADDDGLEGTVSQLHSSNDQMDEDDSFAKRSRKMDGVMDIIPVVKPIREPRVVVQTVSEVDILDDGYRWRKYGQKVVRGNPNPRSYYKCTNARCPVRKHVERASHDPKAVITTYEGKHNHDVPTARTNSHEMAGSAPVTGSSRVRVEENGAISLDLGVGIGHGMENRRNGQLHTLPAETVRSQGQVSSSIVMVVQPAAVAACYSIVNGGMNRFGTIENRVQGTGFETLPLQSSAQYPQNYGMILLGP

>CaWRKY03-2

MEELVDETPRKRLIKELVEGKSFAKQLQSLLQQPNIEHYDGSVLADELVLKIWRSFTQAITELNTLVDSNSILVQTQMEVEKTEEVDQADTGDRSNSELKKKGKQGGKDRRGCYKRRNNSGSWMRESETMNDGCAWRKYGQKSILNSKYPRCYYRCTHKYDQDCRATKQVHIMQENPKLMYHTTYFGNHTCNPAKIRKHINNAQFNHSMLECPPFEVKPKIPSSVTHDSTEEEEESLKGQSDNVSSTMDSYLWEDFMPSSPSAHDSTLASHNSSYFQGLISSEMGDLVKFSDFEAIEFF

>CaWRKY03-3

MDDNNWDLGAVIRNCGINRPSNDITPNLGSESLNFDDDDLNFLDRIFGVDNNNFDYIAPTNFSISRQEKSYDQFDDIINPTTPVSIIANPFKITNQNDNQQIYLPPIQPAQVSQQVFLSSPSGVEARECVPTTTTTTISHECINLQQQLLMWDSTLTMRNPPIQIRKSKNQSIWTTYELFQEELTDDIWSWRKYGQKFIKGSPFPRNYFKCNTSELCQARKQIEKSSKNDCFFLVAYSGMHNHDPPIIRRSFSDWNHSSKYKLPKGINIIPKALKLNASPFSSKSGKRYRASSTLETESTSRKKNKMIVETMKNNVDDKEEENINEDVPKGFEELN

>CaWRKY03-4

MVQQLTFKSSLHRFCLVFHVNTHFPIELISVMDNYGADNTNVEFNRIINELTQGRDLVQQLQLHLNAPNYNSSASFENTREILLHNIQSKFDKALSILQYNSTTGDNSNSPLTHSTSPAIPVFGVSDSPRSSPPHSEDSDRDLESKDPHATRKRKSTTPRWTKQVQIHPGAPLEGTLDDGFSWRKYGQKDILGAKHPRGYYRCTLRHVQGCLATKQVQRSDEDPTIFEVTYRGRHTCSQGGGGGASASNVHPAPLPLVVTIPQNQEPNLGNHEQYQLIPAPHQNSPEILLDFQKNLSISKDDFNFNTHHDHPNNVPYIPPYSNFPSSSSSHVNTDHQDYTFVANFSTIPINNFVENFPPSSNNMSAGTSQMNNADYQFNSMGFESNFPYNYQGFSS

>CaWRKY03-5

MAENQNDWDLWAIVRSCCNMNNSVHDDVISFDNVNSTSVLVDHGVHEDPTHGNSANNARTSTSFQEQSGCAGDFSDSFATENKHYFGLDEVLGLSKNVNTNSRIEPHNPENQTESITDTPLVEHEKKKNKNKKTRYSLSSSSIEAGKAFPYCRKSTERYEVLAEKLSEADQWRWRKYGMKRTGGSPFLKSYYRCNQGEDCPARRHVQQSSTDSNKVIVTYRGQHSHPPPNQHIATVQGNHNAAAPVEDPPFPSSPSTLLFN

>CaWRKY03-6

MEASFKKSNLHGHVFKVEKINADDKGFVEDTKVLKFSKKRELHEDHKSKSSQLQKDYLTSDKEDDQLESAKADMEEVMEENQRLKKHLDRVMKDYQNLQMQFQEISQRGVEKSNNVKHDEAELVSLSLGRTSSDTKIELSKILNKKENVEEEDNLTLGLDCKFQSSANAPTKSSPSNLSPENSLGEVKDEKGTETWPAHKGLKTIRDEEDDVVQQNPTKRAKVSVRIRCDTPTMNDGCQWRKYGQKIAKGNPCPRAYYRCTVAPSCPVRKQVQRCIQDMSILIITYEGTHNHPLPLSATSMAFTTSAAASMLLSGSSTSESGSTSTSTSAISNALNYYLSNTSKPNPFYLPNSSISSSLHSQHPTITLDLTSNSSTSSFPNNHRMPRYNNNSSTNILNFSSFESNPVLPMSWSNANNQAYNNKNQEIPSQSYLQNTISAAPTQTLLQQDTISAATKAITSDPKFQSALAVALTSIIGSRGANRHIDEKSGKDLKVNEPFPVLCSFPSTATSPIKCSPNNTQPEKSLFMRESTSSVQFTASKTKCHKDYTL

>CaWRKY03-7

MPDKDDQAGDLLEELNRVSAENKKLTEMLTVMCQNYNALRNQLTEYLNKQNSTTSTAADNNHDHHSDGSKKRKVENNNNEIVKSVQGLHSESSSSDEDSSNKKPREQHIKTNTCRVYVKTEASDTSLIVKDGYQWRKYGQKVTRDNPSPRAYFKCSFAPTCPVKKKVQRSVEDQSILVATYEGEHNHSKMDGSGPVTTSPSSRLNPKNTLVGANTTTVMPCSSTSIINTPSGPTLTLDLTQPKKLQNDQKKVNSNTSTSNASGQKSKSPGGHDHHQQNRPEFQQLFIDQMASSLTKDPSFQAALAAAISGKFLQNNHTDK

>CaWRKY04-1

MSTGYRPNNFSSKMEENSVQEAAAAGLQSVEKLIRLLSQSQQQQQQNHQQQTNFQNSSSNDYQVVADVAVNKFKKFISLLDKNRTGHARFRRAKPQQNKQQMEESEKQQSSATKIYCPTPIQRLPPLPHNHHQQILIKNGSIERKEAASSTTINFASPSPATSFMSSLTGETESLQNSLSSGFQITNLSQRKCSSMDDIALKCNSAGGSSGRCHCPKKRKSRVKRVVRVPAISMKMADIPPDDYSWRKYGQKPIKGSPHPRGYYKCSSVRGCPARKHVERALDDPAMLIVTYEGEHNHSHSITETPATHVLESS

>CaWRKY04-2

MGEKLKVPAVSALPALTIPPRETFFGGGNMSYFSPGPITLVSSYFSESEHPSFSQLLAGAMASPLAKPLLTKEEEANCKEGNLGYKQNRPMSLMVAHSPFFTPFSPSGLLNSPAFLSPLQSPFGMSHQQALAHVTAQAALSQSYLQGTSAQVLGTSDLDESSLQPQLDTMPSDQQIKKFELPQISQSEEKPYLNSVDKPASDGYNWRKYGQKMVKASECPRSYYKCTHVKCPVRKKVERSVDGHVTEITYKGHHNHELPQPNKRRRDSGAQDGSDCSKANPEIETHTEIETSGLNGAHLAHSEQVSTERASEPPVLKDYDEIVDTATATGKEQDDESNVKRMKTTVETPILFSSHKAESESKIVVQTRSEVDILDDGFKWRKYGQKVVKGNHHPRSYYRCTYPGCNVRKHVERASTDPKAVITTYEGKHNHESPIARNRSHSAAQDSTCQLNEQEIATWRPSLHEKVALHANEIPVCRQLKDEHMAA

>CaWRKY04-3

MFGSSTFQETSNVTSHHYQTINPNFAFHDPLINMNQDHGHNNNKYQDFDTSFLDMLLDGGDQEYYSNYLNNSYSNNVSFYSENPFTQQEISSSTYSTSGNSSTASSFDATLTNIHMNHENSSMGIEKEKKGEKHAIAFRTKTELEILDDGYKWRKYGKKKVKSNTNLRNYYKCSSGDCKVKKRVERDGNDSSYLITTYEGRHNHESPFVIYCHDEMPTSKYLS

>CaWRKY05-1

MIEPWSRRCWHERQEYIQARSGEEVPPEDGYSWRKYGQKLIVGAKYPREHYRCDCRRLSYREATKMVQRSEAEPLSFEVTYGGSNSCGQENKNQNGEHVVLTKETQRDEVGRAAGETPECYTPEMVSTPNTSFNNSSAGVVFSNSNPVFNITNSDLIPTPTSSPYPDTDISLEDDSLTVLFDDVPENARSTYKCNAN

>CaWRKY05-2

MEKESNLQSEYPIEDGNGNLSTFVMNNPNMMSNNFDIEKDYSLTFLLENMFGGTHVDHQDYDFITTTTSNSIFDLLMLPPHQPIITTSTVQDSTISDQLINAPVTPNVSSISSTSTELPADDYQQEKKVNQQDGEQDQDKYKKQLKPKRKNQKGKREPRFAFMTKSEIDHLDDGYKWRKYGQKAVKNSPFPRSYYRCTTTSCGVKKRVERSIQDTSIVVTTYEGTHTHSCPVMPRGYAGVHPVTINYGVSIGAGDSGGRTYYGYYTIESLSSAHKVLTTECSPEGRRLRQIL

>CaWRKY05-3

MNNPNMMSNNFDIEKDYSLTFLLENMFGGTHVDHQDYDFITTTTSNSIFDLLMLPPHQPIITTSTVQDSTISDQLINAPVTPNVSSISSTSTELPADDYQQEKKVNQQDGEQDQDKYKKQLKPKRKNQKGKREPRFAFMTKSEIDHLDDGYKWRKYGQKAVKNSPFPRSYYRCTTTSCGVKKRVERSIQDTSIVVTTYEGTHTHSCPVMPRGYAGVHPVTINYGVSIGAGDSGGRTYYGDSSLFSTNNLLQERRF

>CaWRKY05-4

MSSLPPCRTDFLTIPPGISPAALLDSTFILPSSMTPTDNVAQILHCIESTNNQIPQQQQEFTKKQNQFEQRGIFSSSISPNNSSDDGYTWRKYGQKHVKGSNFPRSYYKCTQQTCPVRKKVECAPNGQVIEIVYNGPHNHPKTQHLRRKAMDADSYVVGQENGSSSSSLIWRNDQQLEYNKDVNSCCNELERKPSASVLSDVSSDPMLSNNLKSMNVFESDATHELSSTLNSFDDEDEDLATQEGNFLGDGINEFEFEPKRRKKESYSVEPSLLSRTVREPKVVLQVESETDILEDGYRWRKYGQKVVKGNPNPRSYYKCTSAGCLVRKHVERASDDLKSVITTYEGKHNHEVPSANKTNGVAGHFRSASMLNNGQQQPACTASRKSLKDSNIRVQFQDLPIPFERKFFMGSEYLRPNFGASYLSDLSFGGGSLQLPDFAIPLPLPSRMSFPARQNEPRLGDFQMNNHLLLPNGASTFLAAGNTRHINDDNNNSRLLKAKDEVQYWT

>CaWRKY06-1

MEEIEEANNAAVENCHRVMSLLSSGTHDRNQYMNLVRETGEAVNKFKKVVTLLNSTLGHARVRKSNKFKTPLPHNILVENPNCKIDDQAKALRSRPIDENRVLEMGGTNVKCNLTLGSPSLELSSNSRNPLNFGQQTHLPSYNYLQQQQQQQRLFLLQQQSFISSLSVDGSVANGSNFHLVGASQSLDQSSFQHKRRCSERGDEGSVKCGSSGKCHCSKKRKHRVKRSIKVPAVSNKLADIPSDEYSWRKYGQKPIKGSPHPRGYYKCSSMRGCPARKHVERCLEDPSMLIVTYEGEHNHPRMASQSANT

>CaWRKY06-2

MTNLGAAKKILGMKIMRDREKCKLYLSQKNYIEKVLHKFNMQNAKPIYLMPPVQLADIWQILAKNIGKQFRDHDKRRSLTAMFSPLVVVLLVGKLPYRLRLLCQLLRMGDTDRRTVRVAAPRMGNLELPPEDGYTWRKYGQKEILGSRFPRCTHQKLYHCPAKKQVQRLDNDPYVFEVTYRSQHICYMSATAPTVPPPSVEEITHQTTTTPPPLLPLPPPTSASLSGHWLSMDIKPQVEAGTSYSTAQFDIQRDFGHASGGSLTSICNVVTACVDGGAGPSGSRFGREVDYQPVVDMADAMFNSGSSSNTSMDIIFSSIDDKWDTTQKKE

>CaWRKY06-3

MEEIEEANRVAVESCHRVITMLSQPHDQKQFGNVARETGEAVHKFKKVATLLNSNLGHARVRKAKKIITPLPQNLLLESPSCKTYDQPKSLQLLPITEIGSNVKSTLTLANPSLELSSHSKNPLQLAQQTPLSSYHFLQQQQQRRYQLQQQQLKQQTDMMYRRSNSGISLNFDSSTCTPTMSSTRSFISSLSIDGSVANLDGNANAFHFIGASRSADQSSFQHRKRCSGRGEEGSVKCGSSGRCHCSKKRKHRVKRSIKVPAISNKLADIPPDEYSWRKYGQKPIKGSPHPRGYYKCSSMRGCPARKHVERCLEEPSMLIVTYEGEHNHSRLPSQSANA

>CaWRKY06-4

MAASSFSFPTSSSFMTTSFTDLLASSDDYPITKGLGDRIAERTGSGVPKFKSLPPPSLPLSPPPFSPSSYFAIPPGLSPTELLDSPVLLSSSNVLPSPTTGSFPAQAFNWKSSSNNQDVKQEEKNCSDFSFQTQVGTAASISQSQTSHVSLGQQAWNYQEPTKQDGLSSDQNANGRSEFNTMQSFMQNNDHSNSGNGYNQSIREQKRSDDGYNWRKYGQKQVKGSENPRSYYKCTYPNCPTKKKVERSLDGQITEIVYKGNHNHPKPQATRRSSSSTASSAIQSYNTQTNEIPDHQSYGSNGTGQIDSVATPENSSISFGDDDHEHTSQKSRSRGDDLDEEEPDSKRWKRESESEGLSALGSRTVREPRVVVQTTSDIDILDDGYRWRKYGQKVVKGNPNPRSYYKCTSPGCPVRKHVERASQDIKSVITTYEGKHNHDVPAARGSGNHSINRPIAPTITNNNSAMAIRPSVTSHQSNYQVPMQSIRPQQFEMRAPFTLEMLQKPNNYGFSGYANSEDSYENQLQDNNGFSRAKNEPRDDMFMESLLC

>CaWRKY06-5

MEFTSLVDTSLDLSFRPRPVLDKLPKQEVQSDFTGLRGDNMGVKNETVDLLEELNRVSSENKKLTEMLTVVCENYNVLRNQMMEYMSTQNGVADDSAGSRKRKAESISNPNNSNSNVNINNNNNNLDVVPGRSSESSSSDEESSCKKLREEHIKAKVTVVSMKTDASDTSLIVKDGYQWRKYGQKVTRDNPCPRAYFRCSFAPTCPVKKKVQRSIEDQSIVVATYEGEHNHPMTSKPEAGGANTTSTSTGSRLNVTTIAGTTASVPCSTTLNPSGPTITLDLTAPKTVEKRDMKMNQSASPTGGNSIHTSTGVEYQNRPEFQQFLIEQMATSLTKDPSFKAALAAAISGKILQHNNQTGRW

>CaWRKY06-6

MENKNKAEYYSPDDEDQENIIHKFGKGRKEREDDKSKPSSPHHKDFMAIDNNIKGVAVNVMVKRERSPPELNSMASSSAHKEKDDQLALAKVEMREVMEENQRLRFHLDRIMKEYRNLQNQFHDIVQREVDQKSSSTVNTTQHESDHETNELVSLSLGRATSDMKKEELSKILKKDKGRDDEDVNKSLDLGLDCKFEECSPVKNRSPENSLDDHQANKDENGETSTTTWPPNKNLKTMRNDGDNGDDVSQQNPTKRARVSVRVRCDAPTMNDGCQWRKYGQKIAKGNPCPRAYYRCTVAPNCPVRKQVQRCAEDMSILITTYEGTHNHTLPLSATAMASTTSAAANMLLSGSSSSSDPSPQITATTTNTATATTSANINGLNFYISDTSKHKSPFYFPNSSISASTLNNSHPTITLDLTSTSSSSSSSLSHLNRMSNNLHPRYNYNNSSTNLNFSSVLESNSLPISWTNYQNQTCNKNNQNFGSLNFSSRPNQENIFQSYLQKNNNIIPTQSSFPPDTIAAATKAITSDPNFHSALAAALTSIIGNTGIENKPGHNFNVSEPFPVLSSLPSSSNPNKCSSSFLNKPTSSSANNSSQQPGNNNNLVFFAQSSSSSLPFSTSNKGKSTSPSDS

>CaWRKY07-1

MFSIGFTTNHDPLVLDKLKSEVNFNRRDMDRISVTRPGTMNAQARTKHQQRVPDESSTLELSSTSVAQSISSVPSPTLAESRLSAVVNCGTGEVAKQSSDAKVQPLVPVKTSNRDGYNWRKYGQKQVKSPQGTRSYYRCTHFECCAKKIECSGHTNRVMEIIYRSEHNHDPSPSVTCSRESKSAILSASTNGKSLIDHPNRNSNETVASSFKENLQESLPIAETANLDSGGSDTDTEINIKEEHCDEPEQKKRSRKSDASCYESVSKPGKKPKLVVHAACDVGISSDGYRWRKYGQKMVKGNPHPRNYYRCSSAGCPVRKHIERAVDSTIALTITYKGVHDHDMPVPKRRHGPPSAPLIAAAAAPASITDMKKPEPLQHQKSTTQWSVDKQGELTSEKLDLGGGKAMGSARTLLSIGFEIKPC

>CaWRKY07-2

MGISEDDMTDEISSRKLKQKQDPDTVINGSESKEKGTCESMSAEVVSNELHKRPNPDALAKVSQSNRDESAHPTTCQGVLNEGQPRRNVDKEIDVSQSNQKDSSLSNVPEENSENVHQEKGPESEGGASESSRVSVLPKEEPYIKSCKSDSPVKEGNISLVVGTASDCSPQIQSKKMEEVVSQSHQERVTYSTMAENAMYKLRPRRNPDTSVQDLPSDEGVRDLPSDQGVTPFSEPEKPFEDGYNWRKYGQKLVRGNMFTRSYYKCTHSNCLAKKQVERSHDGHITNIQYITNHEHPKPLNSPQISPEVVVPSEMRRPDMLMGTPQAEGEKSTALGQACESIEPLESLISAAVESAGGSARDTVPKSLKSGDEGDSNGGRNSKRRKKEVPRSDDMTPPMKSHSEPRHIVQTRSEVDILNDGYRWRKYGQKFVKGNPNPRSYYRCSSAGCPAKKHVERASHDPKLVITTYEGQHEHDIPLSRTVAQNSDSNTTRISGESTAESGGNKHVDN

>CaWRKY07-3

MAKGSGLSFDPDPIKHFLPIPTVLNSFLEPHHQQQQEFPHNKFLFKIEPLSSSMESSTFKNRSPPSTIQFPVNLNCSTTAIHHDHQEEEEEHNNRPVIDEMDFFADKKNGNNSEEADVTTTTNNTINHSDRKDSNTPPPELDFNINTGLHLLTANTYSDQSIVDDGLSPNSEDKRTKSELAVLRAELERMNGENRRLRDMLNQVTSNYSTLQMHMMTMMQQQQQQNQENGQRDGKSTREEVKQQHHSHNSHGGGGGQMVPRQFMDLGLAAAGATGSEAEEASQSSSEGRSGREKSRSPMNNMESRSTCGIGREDSPEKGSPGWGPNKIPRLGNASTNKPADQATEATMRKARVSVRARSEAPMITDGCQWRKYGQKMAKGNPCPRAYYRCTMAAGCPVRKQVQRCAEDRTILITTYEGTHNHPLPPAAMAMASTTSSAARMLLSGSMPSADGLMNSNFFARTLLPCSSSMATISASAPFPTVTLDLTQSPNPLQFPRPPNQFQVPFSNPPHTNILANPAALLPQIFGQALYNQSKFSGLQLSQDLEGQQHPSTMSSSIHPSNHNPLADTVNALTNDPNFTAALAAAITSLIGNPGQSNNTPATTTTANNNNGSVTSNGNNSNNGNNKVANSGFPAN

>CaWRKY07-4

MEDRLYKSSFFHKQEDSTGTPPDNAADSCFSGDEAAEVNMPSPRKSRRGAKRKVISVPIIEADGSRSKGEVYPPPDSWSWRKYGQKPIKGSPYPRGYYRCSSSKGCPARKQVERSCLDPTMLLITYCSDHNHQLPAAAATATKHHHTAAAGATSPSTSTGTAVDINPSAASDTAKKSSPEEQETNIFAGFSEFAGELGWFSDMGTSTLMESASTSATSIVGSTWNDSDVALMLAIREEDQSLYGDLGELPECSFVFRRYSVETTCCGGTG

>CaWRKY07-5

MSDNNPFHHDYAFPFFGENPSIYDHQVENTQNPHQDFDYPSSYMSSLTECLHGGSMDHYNSLSSAFGMNHRTSSSEVVCPPPIDHHHHQELSRKNSVDHHHQIPLTPNSLISSSSNSEPGCHEEDSSKIKKDDQCEDGGDDDKSKKVNKAKKKGEKKQKEPRFAFMTKSEIDNLEDGYRWRKYGQKAVKNSPFPRSYYRCTSQKCSVKKRVERSYQDPSIVITTYEGQHNHHCPATLRGNAAAALLSPASFLSSSQQQLFHNPSEQQLFYNPNLPINNSFYNNYHQHQHQMQPQLGPDNYQYGVFQDMVASLIHKREP

>CaWRKY07-6

MANSHAEQLTNGRTSASSAAAPGGSNGGGAVAKYKLMTPAMLPISRSTCITIPPGLSPSSFLESPLLLSNIKAEPSPTTGSFSKFQTVQGSGGAAAFLLTRGYSSSNSYIERKSSCFEFKNASGSSSTSGSFATEHVISTGFNQQQNDPLKEVQDQSHRQLLVPSSLAKLEMESSKELSISAPVNVDASSKEESLCQPINVDAMNPGGPSNASMQGSHADHKDVSSVTSERSSDDGYNWRKYGQKLVKGSEFPRSYYKCTYPNCEVKKIFERSPEGQITEIVYKGSHDHPKPQLCRRFSPGSLVSIQEDKCEKEACFRGQEVYVEDKFNTNVQTNKIEPGSTPVSPQTDTDALEGAASQMQGTNDDMDEDDQFAKRRKMDGGMDVTPVIKPIREPRVVVQTVSEVDILDDGYKWRKYGQKVVRGNPNPRSYYKCTNAGCPVRKHVERASHDPKAVITTYEGKHNHDVPTARNNNHEMTGSTPVTGGSRIRAEQTNSLSLDLGVGTGYHLDNGNNGQLHTLHNQVQVSRSGMMLVQPGAVVARYGIVHNGMSRFGAIDNRVQGPSFETLPLQPSTQSLQSYGKILLGP

>CaWRKY07-7

MGGFDDHVAIMGDWMPPSPSPRTFFSSLLGDDVGSRSTFQCTNETKSGNLASGPQENVGTFDGNDEAQAAVSEQQPVSDQKMNPRGGLLERMAARAGFNAPKLNTESLRPADMRQNQGVQNQGVRSPYLTIPPGLSPTTLLESPVFLSNSLVQPSPTTGKFPFSSGIESRNSTLMMEDPDNRKENAFESINASSFSFKPVPETAPSLFPGTNSRSWLQVNPPNFSQQGFPNIEVSVHSQNSLQSHRMEATQNLVQNGTLNQASDFPRFSAEMDVKGSNVTPESRTFQTVGSTVEHSPPLDEPQDEDIDQRGGGDPNVVGAPAEDGYNWRKYGQKQVKGSEYPRSYYKCTHPNCPVKKKVERSQEGHITEIIYKGAHNHPKPPPNRRSALGSTNSLGDLRLDGVEQGASGVNGDLGRANIQKAPGSGGGFDWRSNNLDATSSVNLGSEYCNRSAPFPAQNDSQLESGDAVDVSSTFSNDEDEDDRGTHGSISQGYDGEGDESESKRRKLETYSADMTGATRAIREPRVVVQTTSEVDILDDGYRWRKYGQKVVKGNPNPRSYYKCTSAGCNVRKHVERASHDLKSVITTYEGKHNHDVPAARNSSHVNSGASNTHPTAVTAPAQNHLHRPEPAQLQNAMARFDRQPSLGSFGLAGRPQLGPTPGFSYGMNPQGLSSLSMAGFHPNQNKPGEVPIHPYLGQPRPMHDMGFMFPKEEPKVEPMSDPGLNLSNGSNVYQQFMNRLPLGPQM

>CaWRKY08-1

MENNNKSESDNEMEIDLRLKLDAREEENEENKIGEPSQLTEKTQAKDQEIPKNDQELSMLEKEMKRMKEENKVLRMAVEQTMKDYYDLQAKFSAIHQNNHKDHKNFLSLSGNDDSTTSEGLTTRVPKILDIINTTNRTSSPTSHEDDTMDGDQLGLSLTLVSSNSTTSSKLLEMLEEDQRKEKKEDHPTITHQIQNNKSQNLGGLTSHHVTTASPPNRKSRVSVRARCESATMNDGCQWRKYGQKIAKGSPNCPRAYYRCTVAPGCPVRKQVQRCLEDMSILITTYEGTHNHPLPVGATAMASTASAAASFMLVDSSISPLLNNPNSSLNQPLNFPNYHHNLAPNYHHNIPNSSSSSSLIPYNLSMIRNNILNSSDPNSQGNIVLDLTKNISNNHQFPFASSSSNSHEMGHSNWMPKLPNYEGNSLLAGPKLQGEHHYSHNNNNIPPTLAHDENMSAIAADPKFRVAVAAAISSLINKDQSHSTGESNGGSINRHTDS

>CaWRKY08-2

MAANNPSANMLDGSFRSLDSPDSDDFSNHLINFELSDILEIDNWPIQQDPTLIPQYSNYAANQVVNTSSYQEEPSNNIGSSSSKRKEVKDKVAFRMLSQIEILDDGYKWRKYGKKMVKNSPNPRNYYRCSVEGCPVKKRVERDKEDSRYVITTYEGVHNHQGLSPF

>CaWRKY08-3

MDTNLGDKTFSIDLNTNPSLHNTSRSPHDTLDEKLVRMREENKKLVTMLTTLCENYNSLHSHLIELLQKYFSHNEEDNFKFFLRKRKAEGECCENNSDIHFEEASPKRPREITTNVSTVCVKTNPSDQTSLVKDGYNWRKYGQKVTRDNPYPRAYYKCSFAPTCPVKKKVQRSIEDPSILVAVYEGEHNHPHPSQAEITVPLLNQGVTTDPTFLNKLMEEIDTNSLQQHLVEQMASSLTSSPSFTAAVAAAISGKIFEYDLPFK

>CaWRKY08-4

MKEENKKLATMLTTLGENYNSLRTNLIELQQKHSTHEEDNNSKLLSRKRKAEDVCCVNNSDINFEEASPKRPREIITTSISTVSVKTTLSDQTSLVKDGYNWRKYGQKVTRDNPSPRAYYKCSFAPTCPVKKKVQRSVKDPSVLVATYEGEHNHPHPSQAETTAPLVNQGVITNPTFFNKFMEDINTSSLQKDLVAKMVPSLSKNPSFAATVAQPSLEYFLEYDLQLLKWVLRGNISKVMPPGVRIYKQKTLTGETISATQSMDETNKSTPTTITDNTIHARLIKLSYSEKFEINLDEHYIKDSCEDILKNRSRQWRHKLKKIFESARSKEAARKIEVPELTPKNWNKLCDMWSDLEHKKRCQAKKINRSKLKCNHIMDSKAFVVAHAEIGEEHEGWNQTKLTSTKALITRLKKAGHLNKLKLTIESTMTIDEIVDAVLGKKSRYIKGLDYGSKPDITRATQRRAAELEDSIKKVKEKAATVQHDPQKH

>CaWRKY08-5

MEEDWDLHAVVRGCTASSTTTTTSTTTTATSCCSFQPRQDGNFFSFQDPFVPRFDNPTSDFEELHNLYKPFFPKSQVQPQQQVPRSPQNNIIPISPLSVLGGLQDLSAPQPTLKQQQQQQQQQQQHIHQFFNSTRLTQPKQSLSVNGSTNSTITASSSLGVSHTQSPRPKRRKNQLKKVCQVPAEGLSSDMWSWRKYGQKPIKGSPYPRGYYRCSTSKGCLARKQVERNRSDPNMFIVTYTAEHNHPMPTHRNSLAGSTRQKPANSEAGTTASDSNKPTSSSPVSSPACHSTATEKQESSREEKEDIFEDEDEEFGSSNMGLDNMEPADDDFFEGLDELAAQATGDCFSDNFQGSMQLPWLSNNATTTAAGGV

>CaWRKY08-6

MEKVKGLEKKKLISELTQGKEFVKQLKKQIGPLASPEECDLLLGKILSSLEKSLSILNLKALLLEGGINANNSTSSCSSISFLGNNNSPMSEVFDSPSHHLDKNMVSKKRKKSQETNQITISGTGLEGSHEDGFSWRKYGQKDILGANHPRAYYRCTHRHTQGCLATKQVQRSDGNSTIFEVTYKGRHSCKVAQSDIFSLNNQKRQKHNKKQEQEMVIFNSTPNHDAENFNITTKEEVFTPFSFPPTPLNLENIEETKFFCDSMVPLLTSQNQEFGMDYMTLHCSNSDLTELISTPTPTSISNSSFVGDWDLSEDFEPNVIFDIEEFFS

>CaWRKY09-1

MLIMEEEGLIKNSWSYEDELIKELLDDESPFLLAPHEEYYSTSSSETSYSLDVTKSSISSLSKGSFIDDIESDLSMTRNGVQSHDVSHDARNIGLERGLNLMMNKQEALENKYTLRIKTCGNAMADDGYKWRKYGQKSIKNSPYPRSYYKCTNPRCGAKKQVERSSNEPDTFIITYEGLHLHFAYPFITLNPPQFLDQPTKKPKLTNPKAQNNEENASEIDESPKFVNPSPIVDLEDGLGFGEMGSQGLLEDMVPLMIRNPFIKPTNSYSSSCSYSSPPTSPSFSWSNN

>CaWRKY09-2

MASSGGNTNTFMNSFNSNYSFSSSQFMTSSFSDLLSDNNDDNNNNRNWGFSYQRIMNSINKDEVPKFKSFPPSSLPMISSSSPASPSSYLAFPHSLSPSVLLDSPVLFNNSNTLPSPTTGSFGSLNSKEDNSRTSDFSFHSRPATSSSIFHSSAPRNSLDDLITRQQQTTEFSTAKIGVKSEVAPIQSFSQENMQNNPAPMHYCQPSQYVREQKAEDGYNWRKYGQKQVKGSENPRSYYKCTFPNCPTKKKVERNLDGHVTEIVYKGSHNHPKPQSTRRSSAQSIQNLAYSNLDITNQPNAFLENAQRDSLAVTDNSSASFGDEDVDQGSPISKSGENDENEPEAKRWKGDNENEVISSASRTVREPRIVVQTTSDIDILDDGYRWRKYGQKVVKGNPNPRSYYKCTFIGCPVRKHVERASHDLRAVITTYEGKHNHDVPAARGSGSYSMNKPPSGSNNNMPVVPRPSLLANNSNQGMNVSNTLFNTAQVEPPITLQMLQSSGSSSYSGFGTSSGSYMNQMQPTNNSKLISKEEPKDDLFFSSFLN

>CaWRKY09-3

MSDNPFYHDYMGTSGGINTFPFFGENPSNYHDQPIIPNIQNPNHEHQFVPSSYMTLTECLHGSMDYNTLSSVFGMSCSSSSEVVCPHIDNQGSTRKSSVSATAEPILDSPMGDQTSVEVPPTPNSSISSTSNEAGGQEDSSKIKKHMQNKDGQEGRDDKSKKECKATKKGEKKVKEPRFAFMTKSEIDNLEDGYRWRKYGQKAVKNSPFPRNYYRCTTQKCSVKKRVERSYEDASIVITTYEGQHNHHCPAALRGNASFLSSPHFMPSFPPQLFSQMLIPPTSNQNLLITSEAYNNINNNNYHQQNQGSEYNLFGGGTNDASWIQKQEPS

>CaWRKY09-4

MILYAHTPVLYSAAKSVYNLLLLIQRLSLHRIMGTPKEETTDEVFSENLEQKPEPDPATKSELKEKRSFESTSADVVSGELQKRLSPDADKQASKNNEEESTYPPTGQEVSRISQCDKGINVSQSNQEDISLSRVLENPSENVGQLQVLKSEAGASGSSQLSSLPKDSDAKSCGSESGVKRLSGKASDSSDQMQSSNTEILLSQSDQQRVNYPIQKREKALDKLQPRRNPDTSVHGLTSDQGVTLLRVPEKPSEDGYNWRKYGQKLVRGNEYTRSYYKCTYPNCQAKKQVERSHDGHITDIHYIGKHEHPETPSGPQMLPELVLPLQMKQPEIPIISTLEAEGEKSTRPQETCEPSKPSEAPLVLDIVSACGGVKGTPLKRHKSETEVDKDDGSDSKRQKKDIVATVDTPPIKSQSEPRHIVQTVSEVDIINDGQRWRKYGQKIVKGNPNPRSYYRCSVAGCPVKKHVERASHDPKVVITTYEGQHVHNFPTPRDISQISPVPDVVTTAIRTDSRIESGHKHVVESKSESGERKHVGDSRTELGENKHIEKSKPELGGNKHVGDSKSESGESRHIGKSKIESGENKHVGGSRIESGGNKHVEESKLELGGNKHVGESIPESAENKHVGLDMAVHIGAN

>CaWRKY10-1

MGGFDDHVAIFGDWITPSPSPRAFVSSLLVDDVGGWLPLMEHTNESNCRNFNAEPQQNVTALCSTDGKDGARAGASTDQTVKSSAPSEQKPSTRGGGLMERMVARSGFHAPRLNTDGLRPPVLSQNQEARSPYLTIPPGLSPSVLLYSPVLIYNPLVCPSPTTGLLPLASGDESKSLMLTAGIADKRKETAFGSNTSSSFSSNPVNPSSDLSQQLLPQIEVSAHPNNSLQPQSMEVTQSEQIRHGISKFPMLSTEEDFRGSHIKPEVRPFNIVGGSMQHSQTLDEQKDEDTKQRGGGDSKDVNPPAEDGYNWRKYGQNQANGRTYPRSYYKCAYPKCPVKKRVGGYHDCQVMEIIYKGIHNHPKPLSNPISALGSLNSFGDVQLDNVDPSGTGFNSELALATSQQGPTAKGLMWSNNKLEATSLAALHSEYCSGSTTLQSKGAQQGSADAVEVSSVFSNDEDDHGTRGSVLLGYDGAEDEFEPKRRKSTVSDTSGTIRAIREPRVVVHTISEVDIIDDGYRWRKYGQKVVKGNPNPRSYYKCTSSGCNVRKHIQRSPYDQKSVITTYDGKHYHEVPPARTSSQGSSGASKSPSNPITTDAQSHVGRPEPTQVQNTKPAQVQNTKSTQVQNTNERYGRAPQVQNTNERYGRAPQVQNTNERYGRVPSLGSAGPISGFDSFGTNEQQDLSSLNQHQFSVPLNPYIGWQRPVNDAGFVLPEGEAMPDPNLNYSNGSSTYQKIMNGLPPQM

>CaWRKY10-2

MEEDWDLHAVVRSCTPANTASTTTNDCDLVHGILKVDGALMKSLRIEKDYRQFDNDQMTLQRGSTTTSCNPTNNIHYLCSFQPRPNDNNNNSLFCFKDLLEQRILTTNSATDFEESHELCKPFFTASESLTISSPRRGLPISPISVLGRLQDLPPSSQQQQQQHLHQLTNTKPIQPKRPLSSLNGSITNCTLHAQSSRTKRRKNQLKKVCQVAADALSSDMWSWRKYGQKPIKGSPYPRGYYKCSTSKACLARKQVERNRSDPNMFIVTYTAEHNHPMPTHRNSLAGISRHKTANPNKPTSLSPATNSPAPENQESSRDDKEDIFEDDDDEFGKTEPDDDFFDGLDELVIQATGDSLPEKFSGTLQFPWLVNNAATTAAGGG

>CaWRKY10-3

MEINSSVVDMKRLIKELNCGQKFTNELRELMKKHNIMLAEDLLGKIMTSFSKSLSLLYSSAELHQFSQVPMVAFSYCSEESTDSCKPSSLKDQERYNKRRKTSATTIKEASTLVDDGHVWRKYGQKEILNFPHPRNYYRCTHKFDQGCEATKQVQRIQENPPKFRTTYQGHHSCTTYPSISQILFDSSTNEDCSVLLSFNTNKINYHQPYIHSFHSTKQETKGEISSNCFSPNVGQSQSDDHLIRAALSPGSSDHDVNYSSCTTNCSLEMEMKIDMMVDSVDFEDLIPFDF

>CaWRKY10-4

MESFLLQNTISDLEKVMEELNRGKKFTRRLREIIKKPKINVGNEDAYMSSAEDLVGKIMNSFCASLSILSSEESTEEVSQKSMEDSSGSCKTSSLKDRRGCYKRRRTLETSIKETSTLVDDGHGWRKYGQKQILNAKFPRNYFRCTHKFDQGCQASKQVQRIQENPPLFRITYYGHHTCKTFPKVSQMIFDSPNDHEDSNSVLLNFNSGNNHHQFLDMTVETLDFVDLSFEF

>CaWRKY10-5

MEVNEAVKIPIARPVASRPRCPVYKSFSELLTGTVDISSTNVHSEMAVTAIRPKTIRLKPATNHALVGEPSSQVGVSKAPVGFGSDNILQSVEKPKVLYKPIGKLAQRKTIPLLENKGSSVSDQQRVIADSEAHVQSANEVKQQHDPTTESKQSLSEKSGQDKKKVRSTIVSGSTEEVAQSLINTSNVDRPSYDGYNWRKYGQKKVKGSEYPRSYYKCTHLKCPVKKKVERSYDGQITEIVYRGEHNHPKPQPPKRNLSDGHRQTAICNDTSKETNNPAWGNQHPQMSEAYVCRIENQNDRGLTIHSSKVPCFYDPIVAAGMHTAVRNSEDSAVGSKKLKATCDEQKSKRRKIKGPSSGAGTSGESTFPYIPNQSTTDSEITEDGFRWRKYGHKVVKGSSYPRSYYRCTSPKCSVRKFVERTTDDPRAFITTYEGKHNHGVPNRRPNSEASKTSSKSSAMKEKL

>CaWRKY10-6

MEVNEAAKLPIARPVASRPRCPLYKSFSELLTGAVDISSTNVHSEMAITTIRPKTIRLKPATNHALVGEPSSQVGVSEAPVGFGADNILQSVKKPKVLYKPIAKLAPKKTIPLLENKGSSVSDQQRVIADAEAHIQSANEVKQQHDPTTESKQSLSAKSGQDKKKVRSTIVSGSTEEVAQSLINTSNVDRPSYDGYNWRKYGQKQVKGSEYPRSYYKCTHLKCLVKKKVERSYDGQITEIVYRGEHNHPKPQPPKRNLSDGHRRTAICNDTSKETNNPAWSNQHPQMSEAYVCRRENQNDGGLTIHSSKVPCFYDPIVAAGMHTAVRNSEDSAEGSKKLKATCDEQKSKRRKIKCPSSGAGTSGESTFPYIPNQSTTDSEITEDGFRWRKYGQKVVKGSSYPRRYYRCISPKCNVWKFVERTTDDPRAFITTYEGKHNHGVPNRRPNSEASKTSSKSSAMKEKL

>CaWRKY11-1

MDEKDKVDDQLPIESSWSQLNPDDDADHAYFFENNINTNNDTSSILSEFGWNFQPVEENSSSRFDKIDEHLAGNSSITTMSTSPASATAATELTTAKISTDEPVSSSCSDDPPEKSTASGGSSASKPPSDTVSKVKKKGQKRIRQPRFAFMTKSEVDHLEDGYRWRKYGQKAVKNSPFPRSYYRCTNTKCTVKKRVERSSEDSSIVITTYEGQHCHHTVGFPRGGLINHEAAFTSQLSPLPSQYYHPSGVQYPHELVPMTAAAPVESRTMPGETGSSEARRLPETSQPAATDEGLLGDIVPPGMRSK

>CaWRKY11-2

MSHQQVLAQLTAQASQPQSQMHIQPDYSSSSAATALSMSPFQSLTSNTAANQQIPPALDPNTIKESSDVSLSDQRSEPASFVVDKPADDGYNWRKYGQKQVKGSEYPRSYYKCTQPNCPVKKKVERSLDGQVTEIIYKGQHNHQPPQASKRSKESGNPNGNYNLQGTYEPKEGEPSYSLRMKDQESSLANDQISGSSDSEEVGNAETRVDGRDIDERESKRRAVEVQTSEAVCSHRTVAEPRIIVQTTSEVDLLDDGYRWRKYGQKVVKGNPYPRSYYKCTSQGCNVRKHVERAASDPKAVITTYEGKHNHDVPAARNSSHNTANNSTSQLRPHNPVFDKPTAMRRSDFPSNEQQPIALLRFKEEQIT

>CaWRKY11-3

MNKLEKVLPPGFWDVMDHLSVHLVHEARLGDPVQYRWMYPFERTIGKSKRSIKQEYRIEGSMSEAYLAKKTTNFCSYYFASDVPCSRNRPNRVINFQSTRRSYFVQLYDNDKMFDYFLTWFKDYAYDTRNGQFDQFLKDLSWGPIKMAGGDKDKGTANAPKKKLKKASIINRRLTVSTSHIGISSLPLRGNISEPEIPTIYQSITHGQVYPQADMRDMRNRLIIEPDDYGDAVTSWGKMPDNLRNQILLEFREKELGRPITQAEAFKTTHTRKKKISEDPDVWVEPRAQLTYEDQLINSTKCQIVEAKEENERLKWMLSKTMKDYKSLQMHFQGIITQNSNNIIAKSPSNIIEKEEVEHLSLSLGRSSSCSTSKEFKKELYDQNDECKSQKNCKLYDGLDLGLDCKFSQDHLTDDHSPENSFEESNKENIKMARISRNHDEDEDEDEXARVSIRAVCGTTTMNDGCQWRKYGQKIAKGNPCPRAYYRCTVSPTCPVRKQAHFNLQDREEYVAFAKVQDIDHRVRDEGLVQPSPRTSLCLQPLCVTIVVYCSGLQIDLPTGELQSEANLGSKLFRFGCSVSAATVIYEYLVYIPRLALAQAFTINVQDNNQAAVLYQQGEDLVVSRIQDFMRMNPPEFYR

>CaWRKY11-4

MENYPPLFPSSLSSHEFSLMNKKRSNTHAKEVLLFQGKNNGFLGLMASMETPSGVTNSFEDDVMKSCKKKGEKKIKKPRYAFQTRSQVDILDDGYRWRKYGQKAVKNNKFPRSYYRCTHQGCNVKKQVQRLSKDEGVVVTTYEGMHSHPIDKSTDNFEQILSQMQVYASF

>CaWRKY12-1

MENFSYNYSNPNPNNGDIYSSNFIDTPENFELSDTPENFELSGYYLFPEDGLSDEFLSQNEFVQSASDSRGSYSNIKPAPTTTHHNMQVKCTKGVMKKVDAKSRVAFRFRSELEVLDDGYKWRKYGKKMVKNSPNPRNYYKCSNGGCNVKKRVERDNEDSSYVITTYEGIHNHESPHVIHYTQFPPNNIALHNLHL

>CaWRKY12-2

MFSQSLLEDHQDMSSQLGFFSFPPNYNNVGMISTTTTATLPFIGYNQNTLKTLTMNIPPSFDHSLNIQESTHDPRRKEDLSPIFGGPHLHSLQKSTPNTWAWGEVNESSNIIKRREFDHDHNLGVSSIKMKKIKSSRRKVREPRFCFKTMSDVDVLDDGYKWRKYGQKVVKNTQHPRSYYRCTQDNCRVKKRVERLAEDPRMVITTYEGRHVHSPSHDEEDSQASSQLNNLLW

>CaWRKY12-3

MVMFSKMNEELALQEAASTGLKTMEHLIKLAANESVVKVDCREITDFAVAKLKKANAMVGRTGHARFRRGPIQVQAQNEAESQAQTQAKVHDSLTTLSLSPYGYMEKERVLTPTQLSAVAPVHSGLTLDFRKPIVNTGVGTNVGGGAGVMKWKEFFGSMENAIGSSSFLSSIIGEGSGCIADVASLSSSMSLLPTAQVVSAGEQPSAAEKRCREQEQSGDGSGKKRKVFPRKVIRTPIISSKFADIPSDECSWRKYGQKSIKGSPYPRAYYKCTNFPGCPAKKHVERAMDDPMMLIVTYEEEHRHTQAAMQKYNSQMMAFGSMEGKKE

>CaWRKY12-4

MNKELALQEAASTGLKIMEHLIKLTTNESVVQVDYHEITDFAVAKLKKANAMVGRTGHARFRRGPVQVQAQNKAESQAQVQNSLTSLSLSPYGYMEKERVLASMTLSVVASVAAPVQTELTLGFKMPIVNDDVGGAGVGNMKWKDDFGGMKNWINSSSFLSFITGEGSESIVNVVPPPSSSMLLLPIAQVVSAEKQSSATGKRCREQEQSGDGSGKKRKVFPRKVIRTPMISSKITAIPTDECSWRKCGQKLIKGSPYPWAYYKCTSFPGCPAKKHVERAMDDPTMLIVTYEEEHCHTQVAIQEYNSLMMAFGSVKEKKE

>CaWRKY12-5

MFSKMNEELALQKATATELKIMEHLIKLVANEPAVQVDCRKITDFAIAKLKKANSMVGQTSHARFCLGPVQAQNEVKSQAQAMVQVHDSLTSLSLSLYGYMEKERVLAPAPLSVVTPVAASVQTGLMLGFRKSRVNVVAIAGSSGGSSVGDMKWKDAFGSMKNGISSYSFLSSTTGEGLPCSSSMLLRLMAHAMFAGKQPSAIGKRCREQEQSTDGSSKKRKVFQELLIRTPVISSKITDIPTDKCSWRKYVKKHVERAIDDPVMLIVTYEEEHRHTQVAMQEYNSQMVALGSIEEKKE

>CaWRKY12-6

MVGRTGHARLRRAPVQAVQAQAQAKVQVHDSLTSPSLSPHGHMEKERVLAPAPLFVVAHVVALVQTGLMLGFRKPRFNVVVAVGASVGDMKWKDAFGSMKNGISSSFLSFATGEGSRSISSVALPSSSMLLRLMVQAISTGKQPSATGERCHEQEQSSDGSGKKRKVFPKKVIRTPVISSKITDIPEDECSWRKYGQKPIKGSPYPRLITSLPVFSNVQHKKHVEKAMDDPMMLIMTYEEEHRHTQVAM
