# Supplementary material for: The transcriptional reprograming and functional identification of WRKY family members in pepper’s response to Phytophthora capsici infection
Source: BMC Plant Biol. 2020 Jun 3;20:256. doi: 10.1186/s12870-020-02464-7 (PMC7271409; doi:10.1186/s12870-020-02464-7)
Supplement: Supplementary file 8 — Additional file 8: Table S1. Primers used in this study. [file 12870_2020_2464_MOESM8_ESM.doc]

**Table S1** Primers used in this study.

| Gene | Forward primers (5’→3’) | Reverse primers(5’→3’) |
| --- | --- | --- |
| Primers used for construction of VIGS vectors ^a^ | | |
| CaWRKY02-8 | GGGGACAAGTTTGTACAAAAAAGCAGGCTTCCACGCATCTCAAATGTCCAG | GGGGACCACTTTGTACAAGAAAGCTGGGTCTGCCCCTCATCTACTTCTAT |
| CaWRKY06-4 | GGGGACAAGTTTGTACAAAAAAGCAGGCTTCAACCACTCAATTAATCGACC | GGGGACCACTTTGTACAAGAAAGCTGGGTCAAGCAATGACTCCATAAACA |
| CaWRKY03-7 | GGGGACAAGTTTGTACAAAAAAGCAGGCTTCCGTTACAACTTCCCCGTCTA | GGGGACCACTTTGTACAAGAAAGCTGGGTCGCTAAGGCTGCTTGAAAACT |
| CaWRKY06-5 | GGGGACAAGTTTGTACAAAAAAGCAGGCTTCTGCTTCAGTACCTTGCTCTACA | GGGGACCACTTTGTACAAGAAAGCTGGGTCTACCATCTGCCCGTCTGATT |
| CaWRKY02-6 | GGGGACAAGTTTGTACAAAAAAGCAGGCTTCTCACCTGAGATGTCTCGTACA | GGGGACCACTTTGTACAAGAAAGCTGGGTCTCAGGTGGAATATCTGCCAGT |
| CaWRKY08-5 | GGGGACAAGTTTGTACAAAAAAGCAGGCTTCCCGGAAGTACCCGTCAGAAG | GGGGACCACTTTGTACAAGAAAGCTGGGTCGCAGCGAGTTCATCTAACCC |
| CaWRKY01-9 | GGGGACAAGTTTGTACAAAAAAGCAGGCTTCTCTTCTGGTTTGACAGACGAA | GGGGACCACTTTGTACAAGAAAGCTGGGTCTGGATTGTTGAATGGAAAGTTTGT |
| CaWRKY09-2 | GGGGACAAGTTTGTACAAAAAAGCAGGCTTCAAGGCCTTCACTGTTGGCTA | GGGGACCACTTTGTACAAGAAAGCTGGGTCTCAGTTAAGGAAAGAGCTGAAGA |
| CaWRKY02-4 | GGGGACAAGTTTGTACAAAAAAGCAGGCTTCATGGGTGATGAACTAA | GGGGACCACTTTGTACAAGAAAGCTGGGTCATCCACATGATGAAGA |
| CaWRKY03-4 | GGGGACAAGTTTGTACAAAAAAGCAGGCTTCCACTTGCAGTCAAGGTGGTG | GGGGACCACTTTGTACAAGAAAGCTGGGTCTCAGTGTTAACGTGGGACGA |
| CaWRKY10-4 | GGGGACAAGTTTGTACAAAAAAGCAGGCTTCAATCGCGGCAAGAAATTTAC | GGGGACCACTTTGTACAAGAAAGCTGGGTCACCACTTGAGTCTTCCATCG |
| CaWRKY08-3 | GGGGACAAGTTTGTACAAAAAAGCAGGCTTCCAAGCTGAAATAACAGTG | GGGGACCACTTTGTACAAGAAAGCTGGGTCTTTGAAAGGTAAATCATA |
| CaWRKY10-3 | GGGGACAAGTTTGTACAAAAAAGCAGGCTTCATACCAAGGTCATCACTCG | GGGGACCACTTTGTACAAGAAAGCTGGGTCTCATCTCCATCTCCAAACT |
| CaWRKY08-4 | GGGGACAAGTTTGTACAAAAAAGCAGGCTTCCATTAGTTAACCAAGGTG | GGGGACCACTTTGTACAAGAAAGCTGGGTCATTCCAGAGATGGCTGAGCA |
| CaWRKY03-2 | GGGGACAAGTTTGTACAAAAAAGCAGGCTTCCCAGCAAAGATTCGGAAGCA | GGGGACCACTTTGTACAAGAAAGCTGGGTCACTCTATGGCCTCAAAGTCAC |
| CaWRKY08-2 | GGGGACAAGTTTGTACAAAAAAGCAGGCTTC GCAGCAGCAAGAGGAAAGAA | GGGGACCACTTTGTACAAGAAAGCTGGGTC GGGATAGACCTTGATGGTTGTG |
| CaWRKY12-1 | GGGGACAAGTTTGTACAAAAAAGCAGGCTTCACAATTACTCAAACCCTAACCCT | GGGGACCACTTTGTACAAGAAAGCTGGGTCTCCATCATCCAACACCTCCA |
| CaWRKY03-6 | GGGGACAAGTTTGTACAAAAAAGCAGGCTTCGTCCAATTAAATGTTCTCC | GGGGACCACTTTGTACAAGAAAGCTGGGTCATCAGCAAAGCGAAGTGTC |
| CaWRKY01-10 | GGGGACAAGTTTGTACAAAAAAGCAGGCTTCAACAAGCCAACCACCAAACG | GGGGACCACTTTGTACAAGAAAGCTGGGTCTCCCTTCAATCGAAGAACTTGT |
| Primers used for qRT-PCR analyses | | |
| CaWRKY02-8 | GCAAGCCGAAGATCAGTGTT | TGCTTACCCTGCTTGTCTGA |
| CaWRKY06-4 | ACCCAAATTGTCCCACCAAG | CGACGATCTTCTGGTAGCCT |
| CaWRKY03-7 | AGCTTCAGACCTCTTCGAGT | CCAACAAATCACCTGCCTGA |
| CaWRKY06-5 | TTTGCACCTACCTGTCCTGT | GTAGTACCCGCGATAGTCGT |
| CaWRKY02-6 | TCTCGTTCCACCAAAGACGA | CCACTGCACTTGCCAGATAC |
| CaWRKY08-5 | AACCCATCAAAGGCTCTCCA | GTGGTTGTGCTCAGCTGTAT |
| CaWRKY01-9 | TGAGCAAGCAGCATGTCATT | CGCTGAATAGCCGCTACCTA |
| CaWRKY09-2 | CCAAAGCCTCAATCCACCAG | TGTCTGTTACGGCCAAGGAA |
| CaWRKY02-4 | CACCAAACTCTTCAGTCTCA | AGCATCTTCACCATCTTCTA |
| CaWRKY03-4 | ACGGTGGACAAAGCAAGTTC | GGCCAAACAACCTTGGACAT |
| CaWRKY10-4 | TCGACGAGGATGCTACAAGA | CTTTGCACCTGTTTGCTTGC |
| CaWRKY08-3 | CCTCATTGCACAACACCAGT | CACAACATTCTCCTTCAGCCT |
| CaWRKY10-3 | TCCACCAAAGTTTCGTACCAC | TGACTTTGGCCAACATTAGGG |
| CaWRKY12-1 | GTGAAAAAGAGAGTAGAAAGGGA | TATTGGGAGGAAACTGTGTGTAG |
| CaWRKY01-10 | AGTTAATGTCCGCACAGGGA | ACTTCGCGACAAGTATGGGA |
| CaWRKY08-4 | TCCCAAGCTGAAACAACAGC | ATTCCAGAGATGGCTGAGCA |
| CaWRKY08-2 | GTGCAAACATGCTTGATGGG | AACCACTTGGTTTGCTGCAT |
| CaWRKY03-2 | AGAAGAAGGGCAAACAAGGG | TGTGGGTGCACCTGTAGTAG |
| CaWRKY03-6 | CAACGATGAACGATGGATGC | TGATGTGGCTGAAAGAGGAA |
| *PcActin* | ACTGCACGTTCCAGACGATC | CCACCACCTTGATCTTCATG |
| *CaActin* | AGGGATGGGTCAAAAGGATGC | GAGACAACACCGCCTGAATAGC |

^a^ The underlined sequences represent attB adapters fused to the gene-specific primers used for gateway cloning.
